# What's Normal? Microbiomes in Human Milk and Infant Feces Are Related to Each Other but Vary Geographically: The INSPIRE Study

**DOI:** 10.3389/fnut.2019.00045

**Published:** 2019-04-17

**Authors:** Kimberly A. Lackey, Janet E. Williams, Courtney L. Meehan, Jessica A. Zachek, Elizabeth D. Benda, William J. Price, James A. Foster, Daniel W. Sellen, Elizabeth W. Kamau-Mbuthia, Egidioh W. Kamundia, Samwel Mbugua, Sophie E. Moore, Andrew M. Prentice, Debela Gindola K., Linda J. Kvist, Gloria E. Otoo, Cristina García-Carral, Esther Jiménez, Lorena Ruiz, Juan M. Rodríguez, Rossina G. Pareja, Lars Bode, Mark A. McGuire, Michelle K. McGuire

**Affiliations:** ^1^Margaret Ritchie School of Family and Consumer Sciences, University of Idaho, Moscow, ID, United States; ^2^Department of Animal and Veterinary Science, University of Idaho, Moscow, ID, United States; ^3^Department of Anthropology, Washington State University, Pullman, WA, United States; ^4^Statistical Programs, College of Agricultural and Life Sciences, University of Idaho, Moscow, ID, United States; ^5^Department of Biological Sciences, University of Idaho, Moscow, ID, United States; ^6^Dalla Lana School of Public Health, University of Toronto, Toronto, ON, Canada; ^7^Department of Human Nutrition, Egerton University, Nakuru, Kenya; ^8^Department of Women and Children's Health, King's College London, London, United Kingdom; ^9^MRC Unit The Gambia at the London School of Hygiene and Tropical Medicine, Fajara, Gambia; ^10^MRC International Nutrition Group, London School of Hygiene and Tropical Medicine, London, United Kingdom; ^11^Department of Anthropology, Hawassa University, Hawassa, Ethiopia; ^12^Faculty of Medicine, Lund University, Lund, Sweden; ^13^Department of Nutrition and Food Science, University of Ghana, Accra, Ghana; ^14^Probisearch, Tres Cantos, Spain; ^15^Department of Microbiology and Biochemistry of Dairy Products, Instituto de Productos Lácteos de Asturias (IPLA-CSIC), Villaviciosa, Spain; ^16^Department of Nutrition, Food Science, and Food Technology, Complutense University of Madrid, Madrid, Spain; ^17^Nutrition Research Institute, Lima, Peru; ^18^Larsson-Rosenquist Foundation Mother-Milk-Infant Center of Research Excellence, University of California, San Diego, La Jolla, CA, United States; ^19^Department of Pediatrics, University of California, San Diego, La Jolla, CA, United States

**Keywords:** human milk, breastmilk, feces, microbiome, international, infant, breastfeeding, maternal

## Abstract

**Background:** Microbial communities in human milk and those in feces from breastfed infants vary within and across populations. However, few researchers have conducted cross-cultural comparisons between populations, and little is known about whether certain “core” taxa occur normally within or between populations and whether variation in milk microbiome is related to variation in infant fecal microbiome. The purpose of this study was to describe microbiomes of milk produced by relatively healthy women living at diverse international sites and compare these to the fecal microbiomes of their relatively healthy infants.

**Methods:** We analyzed milk (*n* = 394) and infant feces (*n* = 377) collected from mother/infant dyads living in 11 international sites (2 each in Ethiopia, The Gambia, and the US; 1 each in Ghana, Kenya, Peru, Spain, and Sweden). The V1-V3 region of the bacterial 16S rRNA gene was sequenced to characterize and compare microbial communities within and among cohorts.

**Results:** Core genera in feces were *Streptococcus, Escherichia/Shigella*, and *Veillonella*, and in milk were *Streptococcus* and *Staphylococcus*, although substantial variability existed within and across cohorts. For instance, relative abundance of *Lactobacillus* was highest in feces from rural Ethiopia and The Gambia, and lowest in feces from Peru, Spain, Sweden, and the US; *Rhizobium* was relatively more abundant in milk produced by women in rural Ethiopia than all other cohorts. Bacterial diversity also varied among cohorts. For example, Shannon diversity was higher in feces from Kenya than Ghana and US-California, and higher in rural Ethiopian than Ghana, Peru, Spain, Sweden, and US-California. There were limited associations between individual genera in milk and feces, but community-level analyses suggest strong, positive associations between the complex communities in these sample types.

**Conclusions:** Our data provide additional evidence of within- and among-population differences in milk and infant fecal bacterial community membership and diversity and support for a relationship between the bacterial communities in milk and those of the recipient infant's feces. Additional research is needed to understand environmental, behavioral, and genetic factors driving this variation and association, as well as its significance for acute and chronic maternal and infant health.

## Introduction

Although long thought to be sterile, human milk is now known to contain myriad bacteria, and growing evidence suggests that the composition and profiles of these microbiomes differ among geographically distinct populations of women. The microbiome of milk produced by healthy women is of scientific and public health interest because these microbes may, at least in part, determine which microbial communities are in the gastrointestinal (GI) tracts of their infants (1–5). The infant GI microbiome (often assessed through the analysis of feces) is of substantial interest because its variation has been associated with a variety of human diseases, both in early and later life [reviewed in (6)].

In the first report of a complex bacterial community in human milk using high-throughput methodology, the milk microbiome of healthy women (*n* = 16) in the Moscow, ID/Pullman, WA region of the United States was found to be dominated by *Streptococcus, Staphylococcus, Serratia*, and *Corynebacterium* (7). Bacterial communities appeared to be somewhat unique for each woman, although 9 “core” genera were common all samples. Since the publication of this paper, additional studies have suggested that the primary bacterial taxa in milk vary across populations (Supplementary Table 1). For example, Cabrera-Rubio et al. (8) found that *Leuconostoc, Weisella*, and *Lactococcus* were the most predominant genera in milk produced by women living in Finland (*n* = 18), while Davé et al. (9) reported *Streptococcus, Staphylococcus, Xanthomonadaceae*, and *Sediminibacterium* were the most abundant taxa in milk produced by Mexican-American mothers (*n* = 10). In Chinese and Taiwanese women (*n* = 133), family-level analysis revealed *Streptococcaceae, Pseudomonadaceae, Staphylococcaceae, Lactobacillaceae*, and *Oxalobacteraceae* as the most abundant taxa (10). Other reports suggest additional differences among populations (11–16), although some similarities are notable, such as the dominance of members of the *Streptococcaceae* and *Staphylococcaceae* families. It is unknown, however, if this variation is due to genuine differences among populations, differences in location of milk collection (e.g., hospital vs. home), or differences in sample collection methods, storage, processing, and analyses. This is a persistent problem in microbiome research and can only be solved with rigorously controlled studies of representative cohorts of women from diverse populations, including standardized milk collection protocols.

To this end and to help address other potential confounders, Kumar et al. (17) investigated the influence of geographic location on the milk microbiome by collecting and analyzing milk produced by 80 healthy women (20 each from Spain, Finland, South Africa, and China) at 1 mo postpartum. Substantial differences were found among cohorts, and variation was related to a variety of factors, such as delivery mode. For example, milk produced by Chinese women contained relatively more *Streptococcus* than milk produced by women in all other cohorts, while milk produced by Spanish women had relatively more *Propionibacterium* and *Pseudomonas* than that produced in other locations. This study also demonstrated that milk produced by Spanish and South African women was characterized by relatively higher proportion of bacterial genes involved in lipid, amino acid, and carbohydrate metabolism than that of Finnish women. As such, not only do there appear to be genuine compositional differences in the milk microbiome around the world, but there may also be differences in microbial function.

Similarly, a substantial and growing literature exists regarding the human fecal microbiome during infancy [for example, (2, 18–22)]. These studies also report differences across global populations. For instance, in a study of 6-mo-old Malawian and Finnish infants, *Bifidobacterium* was the most common genus despite other distinct population differences such as a higher relative abundance of *Bacteroidetes-Prevotella* and *Clostridium histolyticum* in the Malawian infants (23). Murphy et al. (12) found that *Staphylococcus, Escherichia-Shigella*, and *Veillonella* were the most abundant microbial genera in infant feces in Ireland. This contrasts with some earlier work. For example, Backhëd et al. (20) reported that at 4 mo, *Bifidobacterium, Lactobacillus, Collinsella, Granulicatella*, and *Veillonella* dominated microbial communities in feces of vaginally delivered infants in Sweden, while *Bifidobacterium, Ruminococcus*, and *Bacteroidetes* dominated feces of infants born via cesarean section in the same location. (24) also reported a high relative abundance of *Bifidobacterium* in feces of Gambian infants over the first 6 mo of life, followed by *Streptococcus* and members of the *Enterobacteriaceae* family. The common taxa in many of these results suggest that there may be some shared patterns in the bacterial community in infant feces (e.g., *Bifidobacterium* and *Bacteroidetes* as the most abundant genera across cohorts). However, it remains unclear whether these commonalities and differences are genuine or are simply due to methodological differences. True differences, as opposed to biases introduced by varying methodology, might indicate that microbial communities are shaped (at least in part) by some combination of genetics, environment, and behavior, and that there may not be a universal or “normal” infant fecal microbiome representative of health or disease.

The primary purpose of the study described here was to, using standardized collection and analysis procedures, characterize and compare human milk and infant fecal microbiomes in selected global regions. Our hypotheses were that: (1) human milk and infant fecal microbiomes vary among cohorts representing populations from selected geographical regions; (2) there exists a “core” group of bacteria common to milk across cohorts; (3) there exists a “core” group of bacteria common to infant feces across cohorts; (4) variation in the milk microbiome is related to variation in the infant fecal microbiome; and (5) milk and fecal microbiomes of mothers and their own infants are more similar to each other than to maternal/infant dyads in other cohorts. Relationships of milk and fecal microbiomes with other important factors such as delivery mode, other milk components, maternal and infant diets, and household composition and childcare parameters will be addressed in subsequent publications.

## Materials and Methods

### Aim, Design, and Setting

**Figure 1:**
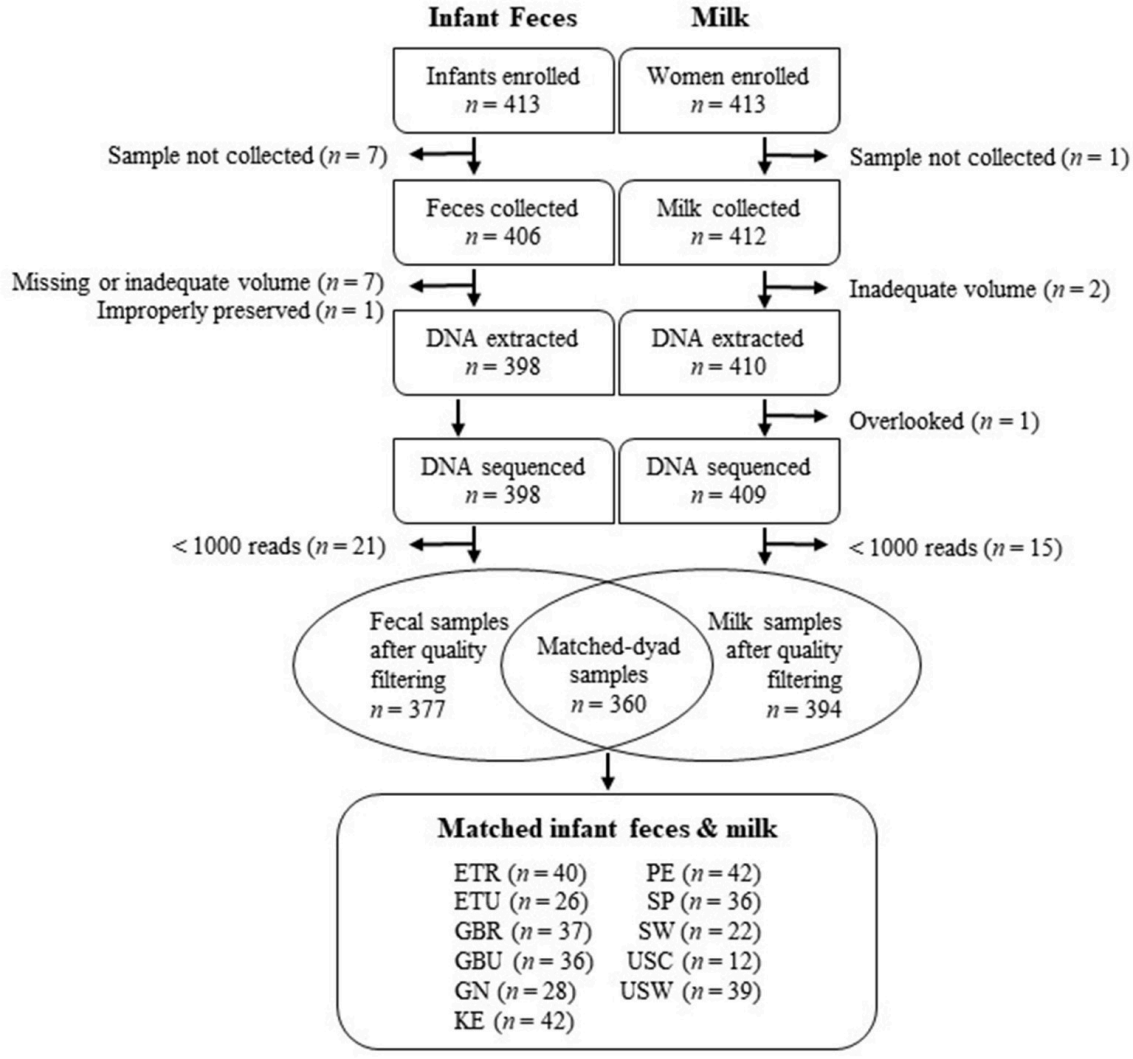
Flowchart depicting the disposition of infant feces and milk included in this study. ETR, rural Ethiopia; ETU, urban Ethiopia; GBR, rural Gambia; GBU, urban Gambia; GN, Ghana; KE, Kenya; SP, Spain; SW, Sweden; PE, Peru; USC, California (United States); USW, Washington (United States).

All study procedures were approved by the Washington State University Institutional Review Board (#13264) and at each study location. Sample collection took place between May 2014 and April 2016 and was carried out as a cross-sectional, epidemiological, multi-cohort study. Informed written or verbal consent was obtained in the local language from each participating woman in her primary language. Informed verbal consent was obtained when a subject's literacy level prevented traditional written consent. Verbal consent, approved and required by the overarching and site-specific IRB boards, was also obtained by team members fluent in the local language. Samples were collected from 11 populations (cohorts), including Ethiopia (a rural population denoted ETR; and an urban population denoted ETU); Kenya (KE), Ghana (GN), The Gambia (a rural population denoted GBR; and an urban population denoted GBU), Peru (PE), Spain (SP), Sweden (SW), and the United States (including a self-identified Hispanic population recruited from California and denoted USC; and an ethnically heterogenous population living in Washington/Idaho, primarily composed of women of northern European descent, denoted USW). Details about these populations have been published previously (25, 26), and subject/sample disposition is summarized in Figure 1. A total of 413 mothers and their infants were enrolled.

For inclusion, women had to be breastfeeding or pumping ≥ 5 times/d and be ≥ 18 y of age. Our goal was to enroll women between 1 and 3 mo (± 7 d) postpartum, although 17 women were outside this target, resulting in a range from 20 to 161 d postpartum. Exclusion criteria for women included (1) current indication of a breast infection or breast pain that the woman did not consider normal for lactation, (2) illness (including fever, vomiting, severe cough, or diarrhea) in the last 7 d, and/or (3) antibiotic use in the previous 30 d. Women did not need to be exclusively breastfeeding to participate. To be included, infants had to be described as healthy by their mothers, have no signs and/or symptoms of acute illness (fever, vomiting, severe cough, diarrhea, or rapid breathing) in the previous 7 d, and have not taken antibiotics in the previous 30 d.

### Anthropometric, Demographic, and Anthropologic Information

Women's height and weight were measured, and body mass index (BMI) calculated. Infants were weighed, and their length measured. Infant weight-for-length z-scores were calculated using the restricted analysis function in the World Health Organization's Anthro igrowup macro (27) using R (version 3.4.1). Weight-for-length z-scores flagged as biologically implausible (< −5 or > 5) were removed from the analysis (*n* = 3). Extensive in-person survey data were collected on aspects of delivery, maternal, and infant characteristics; household composition; maternal and infant diet; and other lifestyle variables. For this study, “exclusively breastfed” was defined as never having received liquids (e.g., water, formula) or semi-solid or solid foods. If an infant received oral medications or non-nutritive dietary supplements (e.g., gripe water, vitamin drops) at any point in his/her lifetime, but was not fed other liquids or foods, he/she was still considered exclusively breastfed. Selected anthropometric and demographic data of the women and infants for whom milk and/or fecal samples were successfully collected and characterized are provided in Table 1.

**Table 1:** Description of the subjects for whom milk and/or fecal samples were successfully collected and characterized.

	**Ethiopia rural**	**Ethiopia urban**	**The Gambia rural**	**The Gambia urban**	**Ghana**	**Kenya**	**Peru**	**Spain**	**Sweden**	**US California**	**US Washington**
Women	*n* = 40	*n* = 34	*n* = 39	*n* = 38	*n* = 37	*n* = 42	*n* = 43	*n* = 40	*n* = 23	*n* = 19	*n* = 39
Age (y)^1^, ^2^	24 ±1^cd^	22 ± 1^d^	26 ±1^bc^	26 ± 1^bc^	28 ±1^bc^	25 ± 1^cd^	26 ±1^bc^	34 ± 1^a^	30 ±1^ab^	29 ± 1^abc^	28 ±1^bc^
	(23–26)	(20–23)	(25–28)	(25–28)	(26–30)	(23–26)	(25–28)	(32–36)	(28–33)	(26–31)	(27–30)
Time postpartum (d)^1^, ^3^	71 ±3^a^	59 ± 4^ab^	65 ±3^ab^	61 ± 3^ab^	58 ±3^ab^	73 ± 3^a^	60 ±3^ab^	71 ± 3^a^	49 ±4^b^	62 ± 5^ab^	68 ±3^a^
	(64–77)	(52–66)	(59–72)	(54–68)	(52–66)	(67–79)	(54–67)	(64–77)	(40–57)	(52–71)	(61–75)
Parity^1^	2.9 ±1.1^ab^	1.4 ± 1.1^de^	3.1 ±1.1^a^	2.7 ± 1.1^abc^	1.9 ±1.1^bcd^	2.2 ±	1.8 ±1.1^cd^	1.2 ± 1.1^e^	1.4 ±1.1^de^	1.6 ± 1.1^cde^	1.6 ±1.1^de^
	(2.5–3.5)	(1.2–1.7)	(2.6–3.7)	(2.3–3.2)	(1.6–2.3)	1.1^abcd^(1.8–2.6)	(1.5–2.1)	(1.0–1.4)	(1.1–1.8)	(1.3–2.1)	(1.4–1.9)
Delivery mode (% vaginal)^1^, ^4^	100^a^	100^a^	100^a^	97^a^	92^a^	78^ab^	51^b^	90^a^	79^ab^	63^ab^	79^ab^
BMI (kg/m^2^)^1^, ^5^	21.1 ±1.0^e^	21.8 ± 1.0^de^	21.2 ±1.0^e^	22.8 ± 1.0^cde^	24.4 ±1.0^bcd^	23.4 ± 1.0^bcde^	27.6 ±1.0^a^	23.2 ± 1.0^cde^	25.5 ±1.0^abc^	28.8 ± 1.0^a^	26.3 ±1.0^ab^
	(20.1–22.2)	(20.7–22.9)	(20.2–22.2)	(21.7–24.0)	(23.3–25.7)	(22.3–24.5)	(26.4–29.0)	(22.1–24.4)	(23.9–27.1)	(26.8–30.9)	(25.0–27.7)
Height (cm)^1^, ^6^	155 ±1^ef^	159 ± 1^de^	162 ±1^cd^	167 ± 1^abc^	159 ±1^de^	159 ± 1^d^	153 ±1^f^	165 ± 1^abc^	169 ±1^a^	162 ± 1^bcd^	167 ±1^ab^
	(153–157)	(157–161)	(160–164)	(165–169)	(157–161)	(158–161)	(151–154)	(163–167)	(166–171)	(159–165)	(165–169)
Infants	*n* = 40	*n* = 32	*n* = 38	*n* = 38	*n* = 32	*n* = 42	*n* = 42	*n* = 37	*n* = 23	*n* = 12	*n* = 41
Sex (% male)^7^	56	50	53	42	55	45	47	48	54	61	44
Exclusively breastfed (%)^1^, ^8^	95^a^	87^abc^	97^a^	53^bcd^	34^d^	36^d^	31^d^	62^abcd^	48^cd^	50^abcd^	57^abcd^
Weight (kg)^1^, ^9^	5.6 ±1.0^ab^	5.4 ± 1.0^ab^	5.2 ±1.0^b^	4.9 ± 1.0^b^	5.2 ±1.0^b^	6.0 ± 1.0^a^	5.5 ±1.0^ab^	5.3 ± 1.0^b^	5.0 ±1.0^b^	5.1 ± 1.1^ab^	5.2 ±1.0^b^
	(5.2–5.9)	(5.1–5.8)	(4.9–5.5)	(4.6–5.1)	(4.9–5.5)	(5.7–6.4)	(5.2–5.8)	(5.0–5.6)	(4.7–5.4)	(4.6–5.7)	(4.9–5.5)
Weight-for-length z-score^1^, ^10^	−0.27 ±0.20^bd^	−0.52 ± 0.22^d^	0.51 ±0.21^abc^	−0.01 ± 0.21^bd^	−0.13 ±0.22^bd^	1.03 ± 0.20^a^	0.60 ±0.19^ab^	−0.29 ± 0.20^bd^	−0.53 ±0.26^cd^	1.37 ± 0.47^ab^	−0.91 ±0.20^d^
	(−0.56–(0.11)	(−0.94–0.09)	(0.11–0.91)	(−0.41–0.39)	(−0.56–0.30)	(0.65–1.42)	(0.22–0.97)	(−0.69–0.11)	(−1.03–0.02)	(0.46–2.29)	(−1.30 to−0.52)

*Values are model-based estimates and represent means ± SEM (95% confidence interval)*.

1 *Effect of cohort (P ≤ 0.05) as determined by one-way ANOVA and a distribution appropriate for the data. Values sharing a letter are not different from each other (P > 0.05)*.

2 Missing values: 2 in The Gambia rural, 1 in The Gambia urban

3 Missing value: 1 in The Gambia rural

4 Missing values: 1 in Ethiopia rural, 1 in Ghana, 1 in Kenya

5 Missing values: 2 in Kenya, 4 in US Washington

6 Missing values: 2 in Kenya, 4 in US Washington

7 Missing values: 1 in Ethiopia rural, 1 in The Gambia rural, 1 in US California

8 Missing value: 1 in US Washington

9 Missing values: 1 in The Gambia rural, 1 in Kenya, 2 in US Washington

10 Missing values: 1 in Ethiopia rural, 2 in The Gambia rural, 2 in The Gambia urban, 3 in Kenya, 5 in US California, 2 in US Washington

### Infant Fecal Sample Collection

Fecal samples (~1 g) were collected from 406 infants. When possible, fecal samples were collected by study personnel at the same time the milk was collected; when this was not possible, mothers collected the next fecal sample available. Samples were collected from provided diapers (Parent's Choice; Walmart, Bentonville, AR) or directly from the infant's skin using a sterile scoop (Sarstedt AG & Co., Nümbrecht, Germany); the sample was then placed in the sterile polypropylene container accompanying the scoop and frozen within 30 min of collection (except ETR) at −20°C. In ETR, because of unreliable electricity, RNAlater® (Ambion) was added to each fecal sample in a ~1:4 ratio (feces: preservative) and frozen within 6 d. All samples were shipped on dry ice to the University of Idaho, Moscow, ID, USA, where they were immediately frozen at −20°C.

### Extraction of DNA From Feces

After thawing feces at room temperature, 0.2 g of each sample was transferred into a sterile tube, 0.5 mL TE50 (10 mM Tris-HCl, 50 mM EDTA, pH 8) added, the mixture vortexed until homogeneous, and then frozen at −80°C until DNA extraction. If < 0.2 g of sample was available, 0.5 mL TE50 was added to the collection tube, the mixture vortexed, and the entire volume transferred to a sterile tube and frozen at −80°C until DNA extraction. Frozen, homogenized fecal samples were quick-thawed on a dry heat block at 37°C and vortexed to re-homogenize. DNA was extracted using the QIAamp® Fast DNA Stool Mini Kit (Qiagen, Germantown, MD) with an additional bead beating step at the beginning using 0.1 mm diameter zirconia/silica beads (BioSpec Products, Inc., Bartlesville, OK) and a FastPrep FP120A-115 (Qbiogene, Carlsbad, CA). For all rounds of DNA extractions, 500 μL TE50 taken from the same aliquot used previously to prepare the samples and 500 μL nuclease-free water (Ambion, Waltham, MA) were extracted as negative controls. Samples were eluted in 200 μL ATE buffer supplied in the kit and stored at −80°C until amplified.

### Milk Sample Collection

Milk was collected from 412 of the women using methods described previously (26). Briefly, both participant and researcher wore nitrile gloves, and milk (~30 mL) was expressed using an electric pump (USW, USC, PE, and SW) and sterile collection kits (Medela, Baar, Switzerland), or hand-expressed into a sterile collection container (all other sites). Except for those collected in ETR where there was unreliable electricity for cold storage, samples were immediately placed on ice, aliquoted into polypropylene cryotubes (Simport Scientific, Saint-Mathieu-de-Beloeil, Quebec) within 30 min, and frozen at −20°C. Milk collected in ETR was preserved in a 1:1 ratio with Milk Preservation Solution (Norgen Biotek, Thorold, Ontario) and frozen within 6 d. We have shown previously that this method can maintain bacterial DNA integrity in human milk held at 37°C for at least 2 wk (28). All samples were shipped on dry ice to the University of Idaho, where they were immediately frozen at −20°C.

### Extraction of DNA From Milk

For all sites except ETR, 1 mL milk was thawed on ice and centrifuged (13,000 x g) for 10 min at 4°C. After removing the lipid and supernatant layers, the cell pellet was resuspended in 500 μL TE50. Samples were subjected to enzymatic lysis by adding 100 μL of a mixture containing 50 μL lysozyme (10 mg/mL in nuclease-free water) (Sigma-Aldrich), 6 μL mutanolysin (25 KU/mL in nuclease-free water) (Sigma-Aldrich), 3 μL lysostaphin (4,000 U/mL in 20 mM sodium acetate) (Sigma-Aldrich), and 41 μL TE50 for 1 h at 37°C. Following the enzymatic lysis, samples were subjected to physical disruption by bead beating with 0.1 mm zirconia/silica beads (BioSpec Products) for 1 min on setting 5 using a FastPrep FP120 (Qbiogene). DNA was extracted using a modified protocol of the DNA Mini Kit (Qiagen), whereby 100 μL 3 M sodium acetate, pH 5.5, was added to the lysate prior to addition to the spin column. DNA was eluted in 50 μL nuclease-free water (Ambion). For each set of milk samples processed in this way, 500 μL TE50 was extracted as a negative control.

DNA in 0.25 mL of each milk sample collected in ETR was extracted using the kit accompanying the Milk Preservation Solution (Norgen Biotek, Thorold, Ontario) as per manufacturer's instructions, including the 2 h enzymatic lysis (20 mg/mL lysozyme). DNA was eluted in 100 μL elution buffer (included with the kit) and stored at −20°C until amplification. Nuclease-free water (500 μL; Ambion) was extracted as a negative control.

### Amplification of Bacterial DNA

For both milk and feces, a dual-barcoded, two-step 30-cycle polymerase chain reaction (PCR) was conducted to amplify the V1-V3 hypervariable region of the 16S rRNA bacterial gene. For the first step, a 7-fold degenerate forward primer targeting position 27 [modified from Frank et al. (29)] and a reverse primer targeting position 534 (positions numbered according to the *Escherichia coli* rRNA gene) were used as described previously (30). The reaction mixture included 12.5 μL Q5® Hot Start High-Fidelity 2X Master Mix (New England Biolabs®, Inc., Ipswich, MA); 0.25 μL each forward and reverse primers (10 μM each); 2 μL template DNA; and 8 μL nuclease-free, sterile water (Ambion) to bring the reaction volume for the first PCR to 23 μL. Nuclease-free water (2 μL) and *Escherichia coli* DNA (2 μL; 221 ng/mL) were used as PCR negatives and positives, respectively. The first PCR was conducted in 96-well plates (USA Scientific, Ocala, FL), using the Veriti model thermal cycler (Applied Biosystems, Foster City, CA) under the following conditions: initial denaturation at 98°C for 30 s; followed by 15 cycles of denaturation at 98°C for 10 s, annealing at 51°C for 20 s, and extension at 72°C for 20 s. After the 15th extension cycle, the thermal cycler was paused at 72°C and the samples removed. For the second step, 2 μL of a unique barcoded primer pair with Illumina adaptors attached [2 μM; obtained from the University of Idaho's Institute for Bioinformatics and Evolutionary Studies (IBEST) Genomics Core facility] were then added to each sample. The plate was vortexed, briefly centrifuged, placed in the thermal cycler, heated to 98°C for 30 s, amplified for an additional 15 cycles as described above (except with an annealing temperature of 60°C), subjected to a 2-min final extension step at 72°C, and held at 4°C until the plate was removed from the thermal cycler.

### DNA Quality, Quantification, and Pooling

PCR products for all negative and positive controls were electrophoresed at 80 V on a 1% agarose gel for 30 min in tris-acetate-ethylenediamine tetraacetic acid (EDTA) buffer (TAE; 40 mM Tris, 20 mM acetic acid, 1 mM EDTA); stained with GelRed^TM^ (10X, Biotium, Fremont, CA); and run alongside a 1-kb ladder (Thermo Fisher Scientific, Grand Island, NY). Gels were visualized using an UltraCam Digital Imaging System (BioRad, Hercules, CA). Amplicon quality was assessed using the QIAxcel DNA Screening cartridge (Qiagen). Briefly, 2 μL PCR product generated in the second amplification was added to 8 μL QX DNA dilution buffer and visualized using high-resolution capillary electrophoresis on a QIAxcel Advanced System (Qiagen). Samples with a peak at the appropriate amplicon size and minimum levels of primer dimers were deemed as having adequate quality. DNA (2 μL amplicon combined with 198 μL of a solution Qubit dsDNA HS reagent and Qubit HS buffer in a 1:200 dilution) was quantified using the Qubit® 2.0 Fluorometer and the Qubit^TM^ dsDNA High Sensitivity Assay (Thermo Fisher Scientific, Waltham, MA). Samples were pooled to contain 50 ng DNA from each sample. If samples amplified poorly, an attempt at re-amplification was made. Subsequently, amplicons were combined if necessary to obtain sufficient amounts of DNA, or the entire volume from the PCR was used.

### Sequencing and Identification of Microbial DNA

Amplicon pools were size-selected using AMPure beads (Beckman Coulter, Indianapolis, IN); quality checked on a Fragment Analyzer (Advanced Analytical Technologies, Inc., Ankeny, IA); and quantified using the KAPA Biosciences Illumina library quantification kit and Applied Biosystems StepOne Plus real-time PCR system. Pools of PCR amplicons for milk and feces were sequenced in two separate sequencing runs by sample type. Sequences were obtained using an Illumina MiSeq (San Diego, CA) v3 paired-end 300-bp protocol for 600 cycles at the IBEST Genomics Core.

Sequence reads were demultiplexed using dbcAmplicons (a custom python application; https://github.com/msettles/dbcAmplicons). During preprocessing, barcodes were allowed ≤ 1 mismatch (hamming distance), and primers were allowed ≤ 4 mismatches (Levenshtein distance) as long as the final 4 bases of the primer perfectly matched the target sequence. Sequence reads without a corresponding barcode and primer sequence were discarded. Sequence reads were also trimmed of their primer sequence. Reads were split into separate sample R1 and R2 files using a custom python script (splitReadsBySample.py; https://github.com/msettles/dbcAmplicons/blob/master/scripts/python/splitReadsBySample.py). Sequence reads were evaluated for quality, trimmed, and filtered using the DADA2 sequence process pipeline (version 1.2.2; (31)). The output from DADA2 infers amplicon sequence variants (ASV) after modeling the errors from a subset of reads (1 × 10^6^) from the sequencing run. Each MiSeq run was analyzed separately for error estimation. Briefly, sequence reads were truncated to 270 bases with a maxEE setting of 4. Reads were also truncated if the base call reached Q2. Reads with < 270 bases were discarded. Because of loss of reads and little overlap between forward and reverse reads following quality filtering and trimming, only forward reads were used in subsequent analyses. ASV were then assigned taxonomies using the Ribosomal Database Project (RDP) Bayesian classifier (32) and the SILVA 16S rRNA database version 123 formatted for DADA2 (33–35). Relative abundances of bacterial taxa at various taxonomic levels were calculated from sequence read count data in R (version 3.4.1) using the phyloseq package (36).

### Designation of Core, Unique, and Aggregated Taxa

A set of “core” genera were characterized for each sample type both in the overall dataset and within each cohort. To be included in the core taxa, a genus must have been present in ≥ 90% of the samples and represent ≥ 0.1% of all identified taxa. We also characterized the relatively “unique” taxa across all taxa identified in the sequencing run for each sample type, defined as genera present in only one cohort and in ≥ 10% of samples within that cohort.

The 10 most-abundant genera from each cohort and for each sample type (based on relative prevalence) were identified and combined to create a set of 28 genera for infant fecal data and a set of 29 genera for the milk data. An “other” category was created at both the phylum and genus levels in these datasets, which is a sum of all other identified taxa within each dataset. These datasets are hereafter referred to as “aggregations” in subsequent text and figures.

### Calculation of Diversity Indices

Diversity indices (richness, Shannon diversity, inverse Simpson diversity, and Fisher diversity) were calculated using phyloseq (36) and sequence read count data. Richness measures the absolute number of taxa present in a population (37), whereas, Shannon diversity is a compound measure of richness and evenness (38, 39). Inverse Simpson diversity is the inverse of the probability that two randomly chosen taxa belong to different genera (38, 40), and Fisher diversity describes the mathematical relationship between the number of genera and the number of individuals within each genus (41). Because some diversity indices (specifically, richness) are linked to the number of reads per sample, we rarefied the infant fecal and milk data to 1,000 reads prior to calculation of all diversity indices.

### Statistical Analyses

All analyses using SAS software were conducted in version 9.3 (SAS Institute Inc., Cary, NC); all other analyses were performed in R [version 3.4.1; (42)]. Significance for all statistical tests were declared at *P* ≤ 0.05. Prior to performing inferential statistical tests, all relative abundance data were rounded to the tenth decimal place. Any taxa denoted as having a relative abundance of zero for all sites after rounding were excluded from further analyses and were not included in tables. For the remaining taxa, all zero values in the dataset were replaced with 1 × 10^−6^. One-way analysis of variance (ANOVAs) were carried out using a generalized linear mixed model (GLIMMIX; SAS) assuming distributions appropriate for the response types. In the case of continuous descriptive variables presented in Table 1, all data except for time postpartum, maternal height, and weight-for-length infant z-scores (which were all normally distributed) were assumed to be log-normally distributed; for binary variables, we assumed a binomial distribution, and for relative abundance (proportional data) we utilized a beta distribution. For infant fecal diversity indices, data were assumed a log-normal transformation; for milk, richness, inverse Simpson, and Fisher diversity values were assumed a log-normal transformation; Shannon diversity values were untransformed. For all ANOVA and associated pairwise comparisons, a Bonferroni correction for multiple comparisons was applied. In the case of multiple comparisons, *P*-values presented are Bonferroni's adjusted *P*.

Hierarchical clustering was performed on relative abundance data using the vegan package (43), specifically the vegdist function and hclust function, in R using a Bray-Curtis dissimilarity matrix and average linkage hierarchical clustering. Non-metric multidimensional scaling (NMDS) plots were created in R using the vegan and ggplot2 (44) packages using rounded data and the aggregate taxa lists for milk and infant feces.

For analyses exploring associations between microbial communities of milk and infant fecal samples, only matched-dyad samples were included (*n* = 360). Heatmaps based upon Spearman rank correlations were constructed in R using the stats (42) and gplots (45) packages to evaluate relationships between the 28 aggregated genera for infant fecal samples and the 29 aggregated genera in milk. Canonical correlation analysis was performed in SAS to explore communities in the data set as a whole (all cohorts combined) using the aggregated infant fecal and aggregated milk genera. For canonical correlations, relative abundance data within each observation were first transformed using a logit transformation. To compare the within- and between-group similarity of bacterial communities, an analysis of similarity (ANOSIM) was performed in R with the vegan package using Bray-Curtis distance and 999 permutations. To evaluate maternal/infant dyad similarity, Bray-Curtis dissimilarity and Jaccard indices were calculated for all matched dyads and all combinations of non-matched dyads, and Wilcoxon test was used to determine differences between these values (14). Associations between diversity indices in milk and infant feces were evaluated using Spearman rank correlations.

## Results

### Description of Subjects

There were many notable differences among the cohorts, as summarized in Table 1. For example, Spanish women were older than women in ETR, ETU, GBR, GBU, GN, KE, PE, and USW (*P* ≤ 0.0017); women in SW were breastfeeding younger infants than women in ETR, KE, SP, and USW (*P* ≤ 0.0328); and there were fewer vaginally-delivered infants in PE than in ETR, ETU, GBR, GBU, GN, and SP (*P* < 0.0001). Women in GBR and ETR had a higher parity than women in all cohorts except GBU and KE (*P* = 0.0411). Maternal BMI was higher in women from PE and USC than women from all African cohorts and SP (*P* ≤ 0.0113), and women in SW were taller than women in ETR, ETU, GBR, GN, KE, PE, and USC (*P* ≤ 0.0174). In addition, exclusive breastfeeding was more common in ETR, ETU, and GBR than in GN, KE, and PE (*P* ≤ 0.0052). Kenyan infants were heavier than infants in GBR, GBU, GN, SP, SW, and USW (*P* ≤ 0.0231); accordingly, infants in KE had a higher weight-for-length z-score than infants in ETR, ETU, GBU, GN, SP, SW, and USW (*P* ≤ 0.0157), though notably, the average z-score for all cohorts was within the normal range (-2 < z < 2).

### Sequencing Summary

The sequencing run for the 398 infant fecal samples generated 4,385,982 reads, with a mean (± standard deviation, SD) of 11,020 ± 6,632 reads and a range of 10 to 40,267 reads following initial processing using the DADA2 workflow. After additional filtering of any read that could not be classified to the genus level, and omitting any sample with < 1,000 reads, the infant fecal dataset analyzed here contained 4,314,551 reads across 377 samples, with a mean (± SD) of 11,444 ± 6,198; and a range of 1,662–40,255 reads. For the 409 milk samples, sequencing generated 7,528,193 reads, with a mean (± SD) of 18,406 ± 18,389 reads and a range of 12−141,620 reads following initial processing using the DADA2 workflow. Using the same filtering criteria as the infant fecal dataset, the milk dataset used here contained 6,709,277 reads across 394 samples, with mean (± SD) of 17,029 ± 16,783, and a range of 1,302–130,700 reads. These curated datasets were used for all further analyses.

### Infant Fecal Microbiome: Individual Phyla Analysis

**Figure 2:**
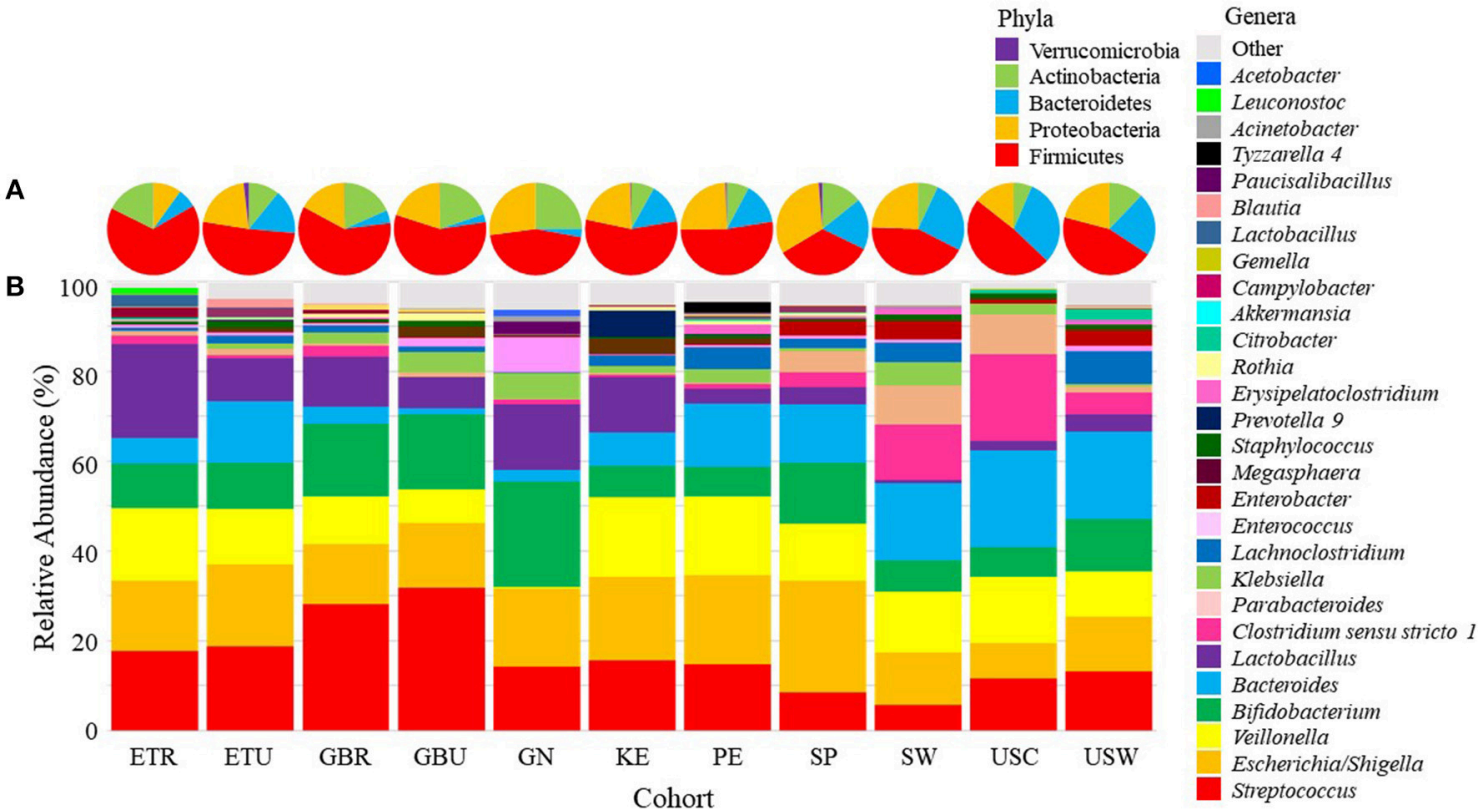
Mean relative abundances of the bacterial **(A)** phyla and **(B)** an aggregation of the 10 most-abundant bacterial genera in each cohort in infant feces. ETR, rural Ethiopia; ETU, urban Ethiopia; GBR, rural Gambia; GBU, urban Gambia; GN, Ghana; KE, Kenya; SP, Spain; SW, Sweden; PE, Peru; USC, California (United States); USW, Washington (United States).

Pie charts illustrating the mean relative abundances of bacterial phyla in infant feces are provided in Figure 2A (mean values available in Supplementary Table 2). Overall, Firmicutes, Proteobacteria, Bacteroidetes, and Actinobacteria together composed > 99.5% of the bacteria identified, though notably only 5 phyla were identified in infant feces; the “other” category, defined as all identified phyla besides the top 4 described above, was entirely composed of Verrucomicrobia (~0.5%). To analyze differences in the relative abundances of these phyla among cohorts, ANOVA was performed and indicated an effect of cohort existed for Firmicutes and Actinobacteria. The relative abundance of Firmicutes was higher in feces from ETR than feces from GN, SP, and USW (*P* ≤ 0.0374) and Actinobacteria was lower in feces from ETR, KE, PE, and USC than feces from GN (*P* ≤ 0.0360).

### Infant Fecal Microbiome: Individual Genera Analysis

There was also variation in the relative abundance of bacterial genera both among (Figure 2B; Table 2) and within (Supplementary Figures 1–11) cohorts. There was a statistical effect of cohort on 8 of the 28 aggregate genera, 7 of which were among the most abundant taxa (Table 2). Differences between cohorts varied by genera. For example, feces from GN, SP, SW, USC, and USW had lower relative abundance of *Streptococcus* than feces from GBR and GBU (*P* ≤ 0.0012). Feces from infants in USW had lower relative abundance of *Escherichia/Shigella* than those in ETU and PE (*P* ≤ 0.0399). Feces from infants in GN had a lower relative abundance of *Veillonella* than infants in ETR, GBR, KE, PE, and SP (*P* ≤ 0.0105). Conversely, GN infants' feces contained relatively more *Bifidobacterium* than feces from ETR, PE, and USC infants (*P* ≤ 0.0310). Relative abundance of *Bacteroides* was higher in feces from infants in USW than GBR, GBU, and KE (*P* ≤ 0.0403). Feces from infants in the two rural populations (ETR and GBR) had higher relative abundance of *Lactobacillus* than feces from PE, SP, SW, USC, and USW (*P* ≤ 0.0122). Relative abundance of *Clostridium sensu stricto 1* was higher in SW than GBU (*P* = 0.0350), and feces from infants in GN had a higher relative abundance of *Enterococcus* than feces from infants in ETR, ETU, GBR, KE, PE, USC, and USW (*P* ≤ 0.0347). For more details, see Table 2.

**Table 2:** Effect of cohort on relative abundances (%) of the aggregate 28 bacterial genera in infant feces.

**Genus**	**Ethiopia rural**	**Ethiopia urban**	**The Gambia rural**	**The Gambia urban**	**Ghana**	**Kenya**	**Peru**	**Spain**	**Sweden**	**US California**	**US Washington**
	***n* =40**	***n* = 32**	***n* =38**	***n* = 38**	***n* =32**	***n* = 42**	***n* =42**	***n* = 37**	***n* =23**	***n* = 12**	***n* =41**
*Streptococcus*^1^	18.2 ± 2.4^bc^	16.0 ± 2.4^bc^	27.7 ± 3.1^ab^	33.6 ± 3.4^a^	12.3 ± 2.0^c^	16.5 ± 2.2^bc^	15.7 ± 2.1^bc^	12.5 ± 1.9^c^	9.0 ± 1.8^c^	7.1 ± 2.0^c^	12.1 ± 1.8^c^
*Escherichia/Shigella*^1^	19.0 ± 2.7^ab^	22.0 ± 3.3^a^	13.5 ± 2.1^ab^	13.6 ± 2.1^ab^	17.5 ± 2.8^ab^	19.0 ± 2.7^ab^	19.9 ± 2.7^a^	16.7 ± 2.6^ab^	12.0 ± 2.4^ab^	9.2 ± 2.6^ab^	10.0 ± 1.6^b^
*Veillonella*^1^	13.7 ± 2.0^a^	9.8 ± 1.7^ab^	13.2 ± 2.1^a^	11.9 ± 1.9^ab^	5.6 ± 1.0^b^	14.3 ± 2.0^a^	14.3 ± 2.1^a^	13.6 ± 2.1^a^	10.9 ± 2.2^ab^	12.4 ± 3.3^ab^	9.6 ± 1.5^ab^
*Bifidobacterium*^1^	8.7 ± 1.4^b^	10.5 ± 1.9^ab^	15.1 ± 2.4^ab^	16.0 ± 2.5^ab^	19.9 ± 3.1^a^	9.9 ± 1.6^ab^	9.3 ± 1.5^b^	11.7 ± 2.0^ab^	9.5 ± 2.0^ab^	5.6 ± 1.7^b^	10.2 ± 1.6^ab^
*Bacteroides*^1^	9.6 ± 1.7^ab^	9.4 ± 1.8^ab^	7.7 ± 1.4^b^	7.3 ± 1.3^b^	9.6 ± 1.8^ab^	8.8 ± 1.5^b^	10.8 ± 1.8^ab^	12.0 ± 2.1^ab^	15.2 ± 3.2^ab^	13.4 ± 3.8^ab^	18.1 ± 2.8^a^
*Lactobacillus*^1^	15.2 ± 2.3^a^	10.6 ± 1.9^ab^	14.7 ± 2.3^a^	9.3 ± 1.6^ab^	12.8 ± 2.2^ac^	12.5 ± 1.9^ac^	5.8 ± 1.0^b^	5.1 ± 0.9^b^	4.6 ± 1.0^b^	4.5 ± 1.3^bc^	5.6 ± 1.0^b^
*Clostridium sensu stricto 1*^1^	3.2 ± 0.6^ab^	3.0 ± 0.6^ab^	2.8 ± 0.6^ab^	2.5 ± 0.5^b^	3.0 ± 0.6^ab^	2.8 ± 0.5^ab^	3.0 ± 0.6^ab^	3.7 ± 0.7^ab^	6.1 ± 1.4^a^	5.9 ± 1.8^ab^	4.1 ± 0.8^ab^
*Lachnoclostridium*	2.4 ± 0.5	2.7 ± 0.6	2.2 ± 0.4	2.4 ± 0.5	2.3 ± 0.5	2.9 ± 0.6	3.3 ± 0.6	2.7 ± 0.6	2.8 ± 0.7	2.1 ± 0.7	3.8 ± 0.7
*Klebsiella*	1.8 ± 0.4	2.6 ± 0.5	2.3 ± 0.5	3.4 ± 0.7	2.7 ± 0.6	2.5 ± 0.5	3.3 ± 0.6	2.2 ± 0.4	2.7 ± 0.6	2.8 ± 0.9	1.9 ± 0.4
*Parabacteroides*	2.1 ± 0.4	2.3 ± 0.5	2.1 ± 0.4	2.1 ± 0.4	2.1 ± 0.5	2.0 ± 0.4	2.0 ± 0.4	2.4 ± 0.5	3.6 ± 0.9	2.4 ± 0.7	2.1 ± 0.4
*Enterococcus*^1^	1.4 ± 0.3^b^	1.2 ± 0.3^b^	1.2 ± 0.2^b^	1.5 ± 0.3^ab^	3.1 ± 0.6^a^	1.1 ± 0.2^b^	1.3 ± 0.2^b^	1.5 ± 0.3^ab^	1.3 ± 0.3^ab^	1.0 ± 0.3^b^	1.4 ± 0.3^b^
*Enterobacter*	1.1 ± 0.2	1.1 ± 0.2	1.1 ± 0.2	1.1 ± 0.2	1.1 ± 0.2	1.2 ± 0.2	1.3 ± 0.3	1.7 ± 0.4	1.6 ± 0.4	1.8 ± 0.5	1.8 ± 0.4
*Megasphaera*	1.0 ± 0.2	1.0 ± 0.2	1.0 ± 0.2	1.3 ± 0.3	0.9 ± 0.2	1.5 ± 0.3	1.1 ± 0.2	0.9 ± 0.2	1.0 ± 0.3	0.9 ± 0.3	1.0 ± 0.2
*Staphylococcus*	0.7 ± 0.1	0.7 ± 0.1	0.9 ± 0.2	1.1 ± 0.2	0.7 ± 0.1	0.6 ± 0.1	0.8 ± 0.2	0.7 ± 0.1	0.9 ± 0.2	0.8 ± 0.2	0.8 ± 0.1
*Prevotella 9*	0.8 ± 0.2	0.9 ± 0.2	0.9 ± 0.2	0.9 ± 0.2	0.8 ± 0.2	1.1 ± 0.2	0.9 ± 0.2	0.8 ± 0.2	0.8 ± 0.2	0.8 ± 0.3	0.8 ± 0.2
*Erysipelatoclostridium*	0.6 ± 0.1	0.7 ± 0.2	0.6 ± 0.1	0.6 ± 0.1	0.7 ± 0.1	0.6 ± 0.1	0.8 ± 0.2	0.6 ± 0.1	0.7 ± 0.2	0.6 ± 0.2	0.8 ± 0.2
*Rothia*	0.4 ± 0.1	0.5 ± 0.1	0.8 ± 0.2	0.8 ± 0.2	0.4 ± 0.1	0.6 ± 0.1	0.5 ± 0.1	0.6 ± 0.1	0.4 ± 0.1	0.4 ± 0.1	0.4 ± 0.1
*Citrobacter*	0.5 ± 0.1	0.5 ± 0.1	0.5 ± 0.1	0.5 ± 0.1	0.5 ± 0.1	0.5 ± 0.1	0.5 ± 0.1	0.5 ± 0.1	0.5 ± 0.1	0.5 ± 0.1	0.6 ± 0.1
*Akkermansia*	0.5 ± 0.1	0.6 ± 0.1	0.5 ± 0.1	0.5 ± 0.1	0.5 ± 0.1	0.5 ± 0.1	0.5 ± 0.1	0.6 ± 0.1	0.5 ± 0.1	0.5 ± 0.1	0.5 ± 0.1
*Campylobacter*	0.8 ± 0.2	0.8 ± 0.2	0.8 ± 0.2	0.8 ± 0.2	0.8 ± 0.2	0.8 ± 0.2	0.7 ± 0.2	0.7 ± 0.2	0.7 ± 0.2	0.7 ± 0.2	0.9 ± 0.2
*Lactococcus*	0.5 ± 0.1	0.4 ± 0.1	0.4 ± 0.1	0.4 ± 0.1	0.4 ± 0.1	0.4 ± 0.1	0.4 ± 0.1	0.4 ± 0.1	0.4 ± 0.1	0.4 ± 0.1	0.4 ± 0.1
*Gemella*	0.3 ± 0.1	0.3 ± 0.1	0.5 ± 0.1	0.5 ± 0.1	0.2 ± 0.0	0.3 ± 0.1	0.3 ± 0.1	0.3 ± 0.1	0.3 ± 0.1	0.3 ± 0.1	0.3 ± 0.1
*Tyzzerella 4*	0.4 ± 0.1	0.4 ± 0.1	0.4 ± 0.1	0.4 ± 0.1	0.4 ± 0.1	0.4 ± 0.1	0.4 ± 0.1	0.4 ± 0.1	0.4 ± 0.1	0.4 ± 0.1	0.4 ± 0.1
*Blautia*	0.3 ± 0.1	0.4 ± 0.1	0.3 ± 0.1	0.3 ± 0.1	0.3 ± 0.1	0.3 ± 0.1	0.4 ± 0.1	0.3 ± 0.1	0.4 ± 0.1	0.3 ± 0.1	0.4 ± 0.1
*Paucisalibacillus*	0.5 ± 0.1	0.5 ± 0.1	0.5 ± 0.1	0.5 ± 0.1	0.5 ± 0.1	0.5 ± 0.1	0.5 ± 0.1	0.5 ± 0.1	0.5 ± 0.1	0.5 ± 0.2	0.5 ± 0.1
*Leuconostoc*	0.2 ± 0.0	0.2 ± 0.0	0.2 ± 0.0	0.2 ± 0.0	0.2 ± 0.0	0.2 ± 0.0	0.2 ± 0.0	0.2 ± 0.0	0.2 ± 0.0	0.2 ± 0.1	0.2 ± 0.0
*Acinetobacter*	0.2 ± 0.0	0.2 ± 0.0	0.2 ± 0.0	0.2 ± 0.0	0.2 ± 0.0	0.2 ± 0.0	0.2 ± 0.0	0.2 ± 0.0	0.2 ± 0.0	0.2 ± 0.0	0.2 ± 0.0
*Acetobacter*	0.1 ± 0.0	0.1 ± 0.0	0.1 ± 0.0	0.1 ± 0.0	0.1 ± 0.0	0.1 ± 0.0	0.1 ± 0.0	0.1 ± 0.0	0.1 ± 0.0	0.1 ± 0.0	0.1 ± 0.0

*Values are model estimates based on a beta distribution and represent means ± SEM*.

1 *Effect of cohort (P ≤ 0.05) as determined by one-way ANOVA; values sharing a letter are not different from each other (P > 0.05)*.

### Infant Fecal Microbiome: Community Analysis

**Figure 3:**
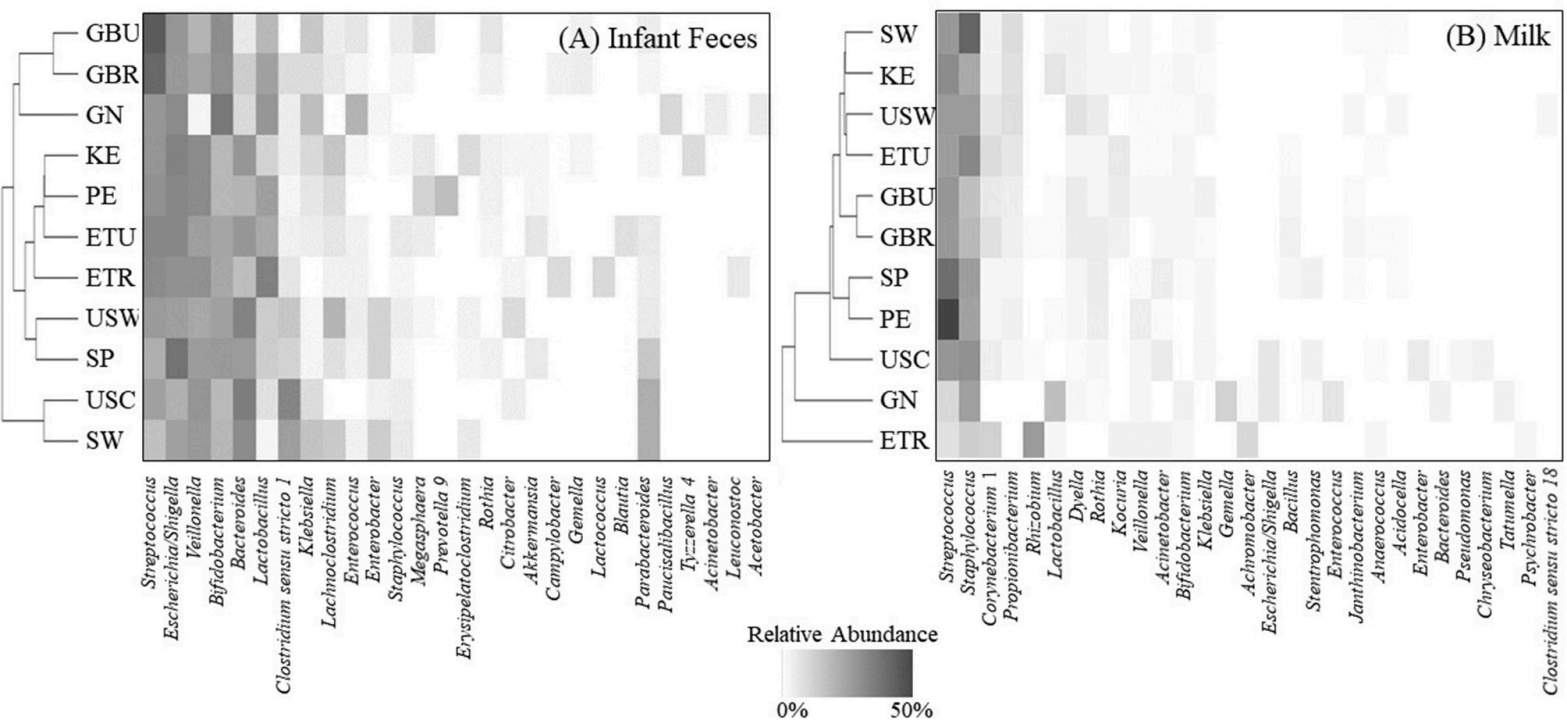
Hierarchical clustering (vertical axis) of the mean relative abundances for an aggregation of the 10 most-abundant bacterial genera from each cohort in **(A)** infant feces and **(B)** milk. ETR, rural Ethiopia; ETU, urban Ethiopia; GBR, rural Gambia; GBU, urban Gambia; GN, Ghana; KE, Kenya; SP, Spain; SW, Sweden; PE, Peru; USC, California (United States); USW, Washington (United States).

Considering similarities and differences among the collective relative abundances of the aggregate genera, hierarchical clustering patterns (Figure 3A) suggest that fecal bacterial community structure in USC and SW, as compared to African cohorts, clustered together and were characterized by relatively high amounts of *Bacteroides, Clostridium sensu stricto 1*, and *Parabacteroides*, but relatively low amounts of *Lactobacillus*. Fecal microbial communities in GBU were closely related to those in GBR and GN, a clade characterized by high relative abundance of *Streptococcus, Bifidobacterium*, and *Lactobacillus*, and low abundance of *Bacteroides*. GN differed from GBU and GBR in that *Veillonella* was low in GN.

NMDS plots suggested no clear clustering by cohort (Supplementary Figure 12A). Although there was considerable variation in fecal bacterial composition among infants within a cohort (Supplementary Figures 1–11), there was more similarity within a cohort than among a random subsampling across cohorts (ANOSIM R = 0.1318, *P* < 0.001). In other words, samples within a cohort were more similar to each other than would be expected by random chance.

### Infant Fecal “Core” and “Unique” Bacteria

Overall, *Streptococcus, Escherichia/Shigella*, and *Veillonella* were identified as core taxa in infant feces, being present in 98.4, 91.7, and 90.2% (respectively) of all samples (Table 3; Supplementary Figure 13). When considered within each cohort, there were sometimes different sets of core taxa. For example, *Lactobacillus* was part of the core taxa for ETR, GBR, GN, and KE, and *Bacteroides* was part of the core taxa for USW. There were no unique bacterial genera identified within any cohort.

**Table 3:** Percent of infant feces and milk samples with quantifiable amounts of niche-specific “core” genera, defined as being present in ≥ 90% of the samples in a single cohort (cohort core) or in the overall datasets (overall core).

**Genus**	**Ethiopia rural**	**Ethiopia urban**	**The Gambia rural**	**The Gambia urban**	**Ghana**	**Kenya**	**Peru**	**Spain**	**Sweden**	**US California**	**US Washington**	**Overall**
**Infant Feces**	*n* = 40	*n* = 32	*n* = 38	*n* = 38	*n* = 32	*n* = 42	*n* = 42	*n* = 37	*n* = 23	*n* = 12	*n* = 41	*N* = 377
*Streptococcus*	**100**	**96.9**	**100**	**100**	**100**	**100**	**100**	**97.3**	**95.7**	83.3	**97.6**	**98.4**
*Escherichia/Shigella*	**100**	**96.9**	89.5	86.8	**96.9**	**95.2**	**97.6**	86.5	**91.3**	75.0	80.5	**91.7**
*Veillonella*	**95.0**	84.4	**94.7**	**94.7**	75.0	**90.5**	**92.9**	**94.6**	87.0	**91.7**	87.8	**90.2**
*Bifidobacterium*	80.0	81.3	**92.1**	**97.4**	**100**	85.7	85.7	89.2	82.6	50.0	87.8	87.0
*Lactobacillus*	**92.5**	87.5	**97.4**	86.8	**90.6**	**92.9**	64.3	48.6	47.8	41.7	61.0	76.7
*Bacteroides*	70.0	53.1	47.4	42.1	75.0	54.8	69.0	70.3	87.0	75.0	**95.1**	66.0
**Milk**	*n* = 40	*n* = 34	*n* = 39	*n* = 38	*n* = 37	*n* = 42	*n* = 43	*n* = 40	*n* = 23	*n* = 19	*n* = 39	*N* = 394
*Staphylococcus*	**100**	**100**	**92.3**	**97.4**	**100**	**100**	**100**	**100**	**100**	**100**	**100**	**98.7**
*Streptococcus*	**100**	**100**	**100**	**97.4**	81.6	**100**	**100**	**100**	**100**	**100**	**97.4**	**97.7**
*Propionibacterium*	75.0	82.4	74.4	68.4	31.6	**90.5**	79.1	82.5	87.5	78.9	84.6	75.5
*Corynebacterium 1*	**95.0**	**94.1**	82.1	84.2	21.1	83.3	81.4	52.5	75.0	68.4	71.8	73.7
*Dyella*	7.5	64.7	84.6	84.2	34.2	83.3	86.0	80.0	62.5	42.1	**94.9**	67.4
*Acinetobacter*	**92.5**	61.8	56.4	47.4	7.9	73.8	44.2	55.0	29.2	36.8	30.8	50.3
*Kocuria*	**92.5**	76.5	43.6	65.8	10.5	69.0	18.6	17.5	12.5	0	12.8	40.7
*Rhizobium*	**100**	11.8	30.8	28.9	31.1	4.8	14.0	25.0	4.2	21.1	20.5	25.5
*Brevundimonas*	**100**	11.8	2.6	13.2	2.6	16.7	7.0	15.0	8.3	15.8	15.4	19.7
*Achromobacter*	**100**	2.9	5.1	2.6	2.6	0	2.3	0	0	5.3	5.1	12.4

*Bolded values are genera considered part of the cohort or overall “core” bacteria*.

### Infant Fecal Microbiome: Diversity Measures

Microbial diversity of infant feces also varied by cohort (Table 4). Richness and Fisher diversity of KE feces were higher than those of feces from ETU, GN, PE, SP, and USC (*P* ≤ 0.0127 and ≤ 0.0111, respectively). Conversely, richness and Fisher's diversity score of fecal samples collected in USC were the lowest of all cohorts (*P* ≤ 0.0126, *P* ≤ 0.0164, respectively). This finding, however, may be due to the small sample size in the USC cohort and should therefore be interpreted cautiously. Feces from infants in KE had higher Shannon diversity than those collected in GN and USC (*P* ≤ 0.0448). Feces from GN had a lower inverse Shannon diversity score than those from ETR, ETU, GBR, GBU, GN, KE, and PE (*P* ≤ 0.0354).

**Table 4:** Effect of cohort on microbial diversity indices (rarefied to 1000 reads) of infant feces and milk. Values are model estimates and, except for Shannon diversity which was normally distributed, were back transformed from estimated log values.

**Index**	**Ethiopia rural**	**Ethiopia urban**	**The Gambia rural**	**The Gambia urban**	**Ghana**	**Kenya**	**Peru**	**Spain**	**Sweden**	**US California**	**US Washington**
Infant Feces	*n* = 40	*n* = 32	*n* = 38	*n* = 38	*n* = 32	*n* = 42	*n* = 42	*n* = 37	*n* = 23	*n* = 12	*n* = 41
Richness^1^	36 ± 1^abc^	31 ± 1^bc^	37 ± 1^abc^	38 ± 1^ab^	32 ± 1^bc^	42 ± 1^a^	32 ± 1^bc^	30 ± 1^c^	35 ± 1^abc^	20 ± 1^d^	34 ± 1^abc^
Shannon diversity^1^	2.51 ± 1.04^abc^	2.55 ± 1.04^ac^	2.62 ± 1.04^ac^	2.60 ± 1.04^ac^	2.08 ± 1.04^b^	2.76 ± 1.04^a^	2.48 ± 1.04^abc^	2.36 ± 1.04^abc^	2.51 ± 1.05^abc^	1.95 ± 1.07^bc^	2.37 ± 1.04^abc^
Inverse Simpson diversity^1^	7.94 ± 1.09^a^	8.64 ± 1.10^a^	8.88 ± 1.09^a^	8.38 ± 1.09^a^	5.04 ± 1.10^b^	10.14 ± 1.09^a^	7.82 ± 1.09^a^	7.18 ± 1.09^ab^	8.22 ± 1.12^ab^	5.67 ± 1.17^ab^	7.25 ± 1.09^ab^
Fisher diversity^1^	7.42 ± 1.06^abc^	6.20 ± 1.07^bc^	7.52 ± 1.06^abc^	7.97 ± 1.06^ab^	6.42 ± 1.07^bc^	8.96 ± 1.06^a^	6.35 ± 1.06^bc^	5.78 ± 1.06^c^	7.02 ± 1.08^abc^	3.65 ± 1.12^d^	6.83 ± 1.06^abc^
Milk	*n* = 40	*n* = 34	*n* = 39	*n* = 38	*n* = 37	*n* = 42	*n* = 43	*n* = 40	*n* = 23	*n* = 19	*n* = 39
Richness^1^	85 ± 1^a^	52 ± 1^b^	57 ± 1^ab^	56 ± 1^ab^	21 ± 1^d^	59 ± 1^ab^	42 ± 1^bc^	39 ± 1^bc^	36 ± 1^bc^	28 ± 1^cd^	44 ± 1^bc^
Shannon diversity^1^	3.16 ± 0.13^a^	2.90 ± 0.14^abc^	3.09 ± 0.13^ab^	3.11 ± 0.13^ab^	2.05 ± 0.13^d^	2.96 ± 0.12^abc^	2.50 ± 0.12^cd^	2.52 ± 0.13^bcd^	2.34 ± 0.16^cd^	2.33 ± 0.18^cd^	2.67 ± 0.13^abc^
Inverse Simpson diversity^1^	10.32 ± 1.13^ab^	9.70 ± 1.14^abc^	11.44 ± 1.13^ad^	12.08 ± 1.13^a^	5.44 ± 1.13^c^	9.36 ± 1.12^abc^	6.49 ± 1.12^bcd^	7.08 ± 1.13a^abc^	5.46 ± 1.17^bc^	6.22 ± 1.19^abc^	7.95 ± 1.13^abc^
Fisher diversity^1^	22.71 ± 1.12^a^	11.84 ± 1.14^bc^	13.47 ± 1.13^abc^	12.98 ± 1.13^abc^	3.93 ± 1.13^e^	14.00 ± 1.12^ab^	8.87 ± 1.12^bcd^	8.07 ± 1.12^cd^	7.40 ± 1.16^cde^	5.30 ± 1.19^de^	9.47 ± 1.13^bcd^

*Values represent means ± SEM*.

1 *Effect of cohort (P ≤ 0.05) as determined by one-way ANOVA; values sharing a letter are not different from each other (P > 0.05)*.

### Milk Microbiome: Individual Phyla Analysis

**Figure 4:**
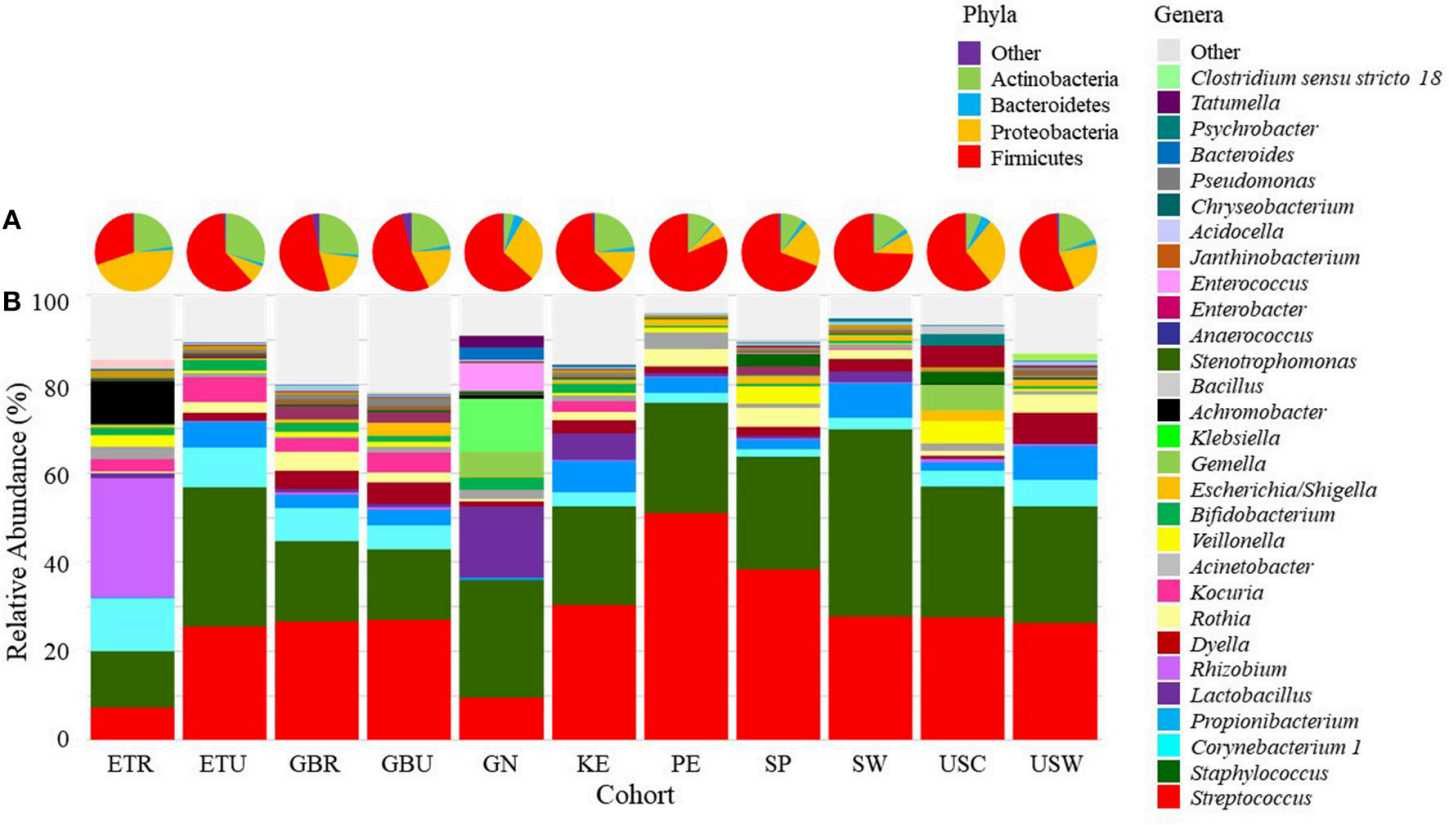
Mean relative abundances of bacterial **(A)** phyla and **(B)** an aggregation of the 10 most-abundant genera in milk in each cohort. ETR, rural Ethiopia; ETU, urban Ethiopia; GBR, rural Gambia; GBU, urban Gambia; GN, Ghana; KE, Kenya; SP, Spain; SW, Sweden; PE, Peru; USC, California (United States); USW, Washington (United States).

A total of 15 phyla were identified in milk, with Firmicutes, Proteobacteria, Actinobacteria, and Bacteroidetes collectively representing 97.7% of those identified (Figure 4A; Supplementary Table 3). There was an effect of cohort on Firmicutes, Proteobacteria, Actinobacteria, Bacteroidetes, and “other.” ANOVA analysis indicated that the relative abundance of Firmicutes was lower in ETR than in all cohorts besides GBR and USC (*P* ≤ 0.0139). Proteobacteria was relatively more abundant in milk collected in ETR than all other cohorts (*P* ≤ 0.0077). Actinobacteria in milk was more abundant in ETR, ETU, GBR, and GBU than in GN, USC, SP, and PE (*P* ≤ 0.0014). Relative abundance of Bacteroidetes was higher in KE than GN (*P* = 0.0060). There was also an effect of cohort on the “other” category; relative abundance in GBR was higher than in ETR, ETU, GN, PE, SP, SW, and USC (*P* ≤ 0.0036).

### Milk Microbiome: Individual Genera Analysis

There was also variation in milk microbiome at the genus level both within and among cohorts (Supplementary Figures 14–24 and Figure 4B, respectively). Of the 29 genera evaluated, ANOVA indicated that there was an effect of cohort on 19 of them (Table 5). Examples of differences among cohorts include a higher relative abundance of *Rhizobium, Achromobacter*, and *Psychrobacter* in milk collected in ETR than all other cohorts (*P* < 0.0001). Except for ETU, milk collected in ETR also had a higher relative abundance of *Corynebacterium 1* than all other cohorts (*P* ≤ 0.0417). Peruvian milk bacterial communities, on average, were comprised of 50% *Streptococcus*, which was relatively higher than all African (ETR, ETU, GBR, GBU, GN, and KE) and US (USW and USC) samples (*P* ≤ 0.0386). Milk from women in USW had relatively more *Dyella* than all sites except The Gambia (GBR and GBU) (*P* ≤ 0.0040).

**Table 5:** Effect of cohort on relative abundances (%) of the aggregate 29 bacterial genera in milk.

**Genus**	**Ethiopia rural**	**Ethiopia urban**	**The Gambia rural**	**The Gambia urban**	**Ghana**	**Kenya**	**Peru**	**Spain**	**Sweden**	**US California**	**US Washington**

	***n =* 40**	***n =* 34**	***n =* 39**	***n =* 38**	***n =* 37**	***n =* 42**	***n =* 43**	***n =* 40**	***n =* 23**	***n =* 19**	***n =* 39**
*Streptococcus*^1^	15.3 ± 2.2^cd^	25.9 ± 3.5^bc^	29.5 ± 3.5^b^	26.0 ± 3.3^bc^	8.7 ± 1.4^d^	31.5 ± 3.5^b^	49.6 ± 3.9^ae^	36.5 ± 3.8^ab^	29.5 ± 4.5^bce^	25.7 ± 4.6^bc^	26.5 ± 3.3^bc^
*Staphylococcus*^1^	21.5 ± 3.0^abc^	34.2 ± 4.3^ab^	17.5 ± 2.6^c^	20.5 ± 3.0^bc^	20.6 ± 3.0^abc^	27.7 ± 3.4^abc^	29.6 ± 3.5^abc^	27.3 ± 3.5^abc^	40.7 ± 5.6^a^	26.0 ± 4.9^abc^	30.4 ± 3.8^abc^
*Corynebacterium 1*^1^	11.0 ± 1.5^a^	8.8 ± 1.4^abf^	4.8 ± 0.8^bcd^	5.5 ± 0.9^bc^	1.8 ± 0.3^e^	4.8 ± 0.8^bcd^	4.2 ± 0.7^cd^	2.5 ± 0.4^de^	3.9 ± 0.8^cdef^	3.2 ± 0.8^cde^	4.1 ± 0.7^cd^
*Propionibacterium*^1^	3.0 ± 0.5^ab^	4.4 ± 0.8^a^	3.8 ± 0.7^ab^	3.6 ± 0.7^ab^	2.0 ± 0.4^b^	5.8 ± 0.9^a^	4.1 ± 0.7^ab^	4.3 ± 0.7^a^	6.6 ± 1.3^a^	3.7 ± 2.3^ab^	5.6 ± 0.9^a^
*Rhizobium*^1^	22.5 ± 2.2^a^	1.3 ± 0.3^b^	1.5 ± 0.3^b^	1.5 ± 0.3^b^	1.2 ± 0.2^b^	1.2 ± 0.2^b^	1.3 ± 0.2^b^	1.4 ± 0.3^b^	1.2 ± 0.3^b^	1.4 ± 0.4^b^	1.4 ± 0.3^b^
*Lactobacillus*	7.0 ± 1.3	4.7 ± 0.9	4.3 ± 0.8	4.3 ± 0.8	8.3 ± 1.5	6.7 ± 1.2	4.1 ± 0.8	4.1 ± 0.8	4.3 ± 1.0	3.7 ± 0.9	3.8 ± 0.7
*Dyella*^1^	0.9 ± 0.2^d^	1.8 ± 0.3^bcd^	3.6 ± 0.6^ab^	3.6 ± 0.6^ab^	1.2 ± 0.2^d^	2.9 ± 0.5^bc^	2.7 ± 0.4^bc^	2.6 ± 0.4^bc^	2.0 ± 0.4^bcd^	1.3 ± 0.3^cd^	6.4 ± 0.9^a^
*Rothia*^1^	1.7 ± 0.3^ab^	3.0 ± 0.6^a^	3.5 ± 0.6^a^	3.1 ± 0.6^a^	1.3 ± 0.3^b^	2.4 ± 0.4^ab^	3.3 ± 0.6^a^	3.1 ± 0.5^a^	2.0 ± 0.5^ab^	2.1 ± 0.5^ab^	2.9 ± 0.5^a^
*Kocuria*^1^	4.4 ± 0.7^a^	3.3 ± 0.6^ab^	1.6 ± 0.3^bc^	2.3 ± 0.4^abc^	1.1 ± 0.2^c^	2.2 ± 0.4^abc^	1.2 ± 0.2^c^	1.2 ± 0.2^c^	1.1 ± 0.3^c^	1.0 ± 0.3^c^	1.1 ± 0.2^c^
*Veillonella*^1^	2.0 ± 0.4^ab^	1.2 ± 0.2^b^	1.1 ± 0.2^b^	1.1 ± 0.2^b^	1.3 ± 0.2^b^	1.6 ± 0.3^ab^	2.8 ± 0.5^a^	1.4 ± 0.3^ab^	1.7 ± 0.4^ab^	1.8 ± 0.4^ab^	1.3 ± 0.2^b^
*Bifidobacterium*^1^	2.5 ± 0.4^a^	2.4 ± 0.4^ac^	1.6 ± 0.3^ab^	1.3 ± 0.2^ab^	1.4 ± 0.3^ab^	1.3 ± 0.2^ab^	1.0 ± 0.2^b^	1.0 ± 0.2^b^	1.0 ± 0.2^bc^	0.8 ± 0.2^b^	0.9 ± 0.2^b^
*Acinetobacter*^1^	3.3 ± 0.6^ac^	1.7 ± 0.3^ab^	1.6 ± 0.3^ab^	1.5 ± 0.3^b^	1.0 ± 0.2^b^	2.0 ± 0.4^ab^	1.4 ± 0.3^b^	1.6 ± 0.3^ab^	1.2 ± 0.3^b^	1.3 ± 0.3^bc^	1.2 ± 0.2^b^
*Klebsiella*^1^	2.2 ± 0.4^b^	2.1 ± 0.5^b^	2.1 ± 0.4^b^	2.2 ± 0.4^b^	7.0 ± 1.3^a^	2.3 ± 0.5^b^	2.1 ± 0.4^b^	2.1 ± 0.4^b^	2.2 ± 0.5^b^	2.3 ± 0.6^b^	2.2 ± 0.5^b^
*Gemella*^1^	1.5 ± 0.3^ab^	1.4 ± 0.3^ab^	1.4 ± 0.3^ab^	1.4 ± 0.3^ab^	1.1 ± 0.2^b^	1.6 ± 0.3^ab^	2.8 ± 0.5^a^	2.0 ± 0.4^ab^	2.9 ± 0.7^a^	1.7 ± 0.4^ab^	1.6 ± 0.3^ab^
*Achromobacter*^1^	8.1 ± 0.7^a^	0.3 ± 0.1^b^	0.3 ± 0.1^b^	0.3 ± 0.1^b^	0.3 ± 0.1^b^	0.3 ± 0.1^b^	0.3 ± 0.1^b^	0.3 ± 0.1^b^	0.3 ± 0.1^b^	0.3 ± 0.1^b^	0.3 ± 0.1^b^
*Escherichia/Shigella*	1.2 ± 0.2	0.9 ± 0.2	0.9 ± 0.2	0.9 ± 0.2	1.8 ± 0.4	1.0 ± 0.2	0.9 ± 0.2	1.0 ± 0.2	1.0 ± 0.2	1.3 ± 0.3	1.0 ± 0.2
*Bacillus*^1^	0.9 ± 0.2^b^	1.2 ± 0.2^ab^	2.4 ± 0.4^a^	1.5 ± 0.3^ab^	0.8 ± 0.2^b^	1.0 ± 0.2^b^	0.8 ± 0.2^b^	0.8 ± 0.2^b^	0.9 ± 0.2^b^	0.8 ± 0.2^b^	0.8 ± 0.2^b^
*Stenotrophomonas*	0.9 ± 0.2	0.6 ± 0.1	0.7 ± 0.1	0.8 ± 0.2	0.6 ± 0.1	0.6 ± 0.1	0.7 ± 0.1	0.9 ± 0.2	0.7 ± 0.2	0.8 ± 0.2	1.1 ± 0.2
*Enterococcus*	1.1 ± 0.2	0.8 ± 0.2	0.8 ± 0.2	0.9 ± 0.2	1.7 ± 0.3	0.8 ± 0.2	0.8 ± 0.2	0.9 ± 0.2	0.9 ± 0.2	0.8 ± 0.2	0.9 ± 0.2
*Janthinobacterium*^1^	0.3 ± 0.1^c^	0.5 ± 0.1^bc^	0.6 ± 0.1^abc^	0.8 ± 0.1^ab^	0.4 ± 0.1^bc^	0.6 ± 0.1^abc^	0.5 ± 0.1^bc^	0.6 ± 0.1^abc^	0.7 ± 0.1^abc^	0.5 ± 0.1^abcd^	1.1 ± 0.2^ad^
*Anaerococcus*^1^	1.1 ± 0.2^a^	0.7 ± 0.1^ab^	0.5 ± 0.1^b^	0.5 ± 0.1^b^	0.4 ± 0.1^b^	0.6 ± 0.1^ab^	0.6 ± 0.1^b^	0.4 ± 0.1^b^	0.5 ± 0.1^b^	0.4 ± 0.1^b^	0.4 ± 0.1^b^
*Acidocella*^1^	0.2 ± 0^d^	0.3 ± 0.1^bcd^	0.6 ± 0.1^abc^	0.4 ± 0.1^bcd^	0.3 ± 0.1^cd^	0.5 ± 0.1^abcd^	0.4 ± 0.1^bcd^	0.5 ± 0.1^abcd^	0.7 ± 0.2^ab^	0.3 ± 0.1^cd^	1.0 ± 0.2^a^
*Enterobacter*	0.4 ± 0.1	0.4 ± 0.1	0.4 ± 0.1	0.4 ± 0.1	0.4 ± 0.1	0.5 ± 0.1	0.4 ± 0.1	0.5 ± 0.1	0.4 ± 0.1	0.5 ± 0.1	0.6 ± 0.1
*Bacteroides*	0.3 ± 0.1	0.3 ± 0.1	0.3 ± 0.1	0.3 ± 0.1	0.6 ± 0.1	0.4 ± 0.1	0.3 ± 0.1	0.3 ± 0.1	0.4 ± 0.1	0.3 ± 0.1	0.3 ± 0.1
*Pseudomonas*	0.3 ± 0.1	0.3 ± 0.1	0.3 ± 0.1	0.3 ± 0.1	0.2 ± 0	0.3 ± 0.1	0.4 ± 0.1	0.3 ± 0.1	0.3 ± 0.1	0.4 ± 0.1	0.4 ± 0.1
*Chryseobacterium*	0.5 ± 0.1	0.3 ± 0.1	0.3 ± 0.1	0.3 ± 0.1	0.3 ± 0.1	0.4 ± 0.1	0.3 ± 0.1	0.3 ± 0.1	0.3 ± 0.1	0.3 ± 0.1	0.3 ± 0.1
*Tatumella*	0.7 ± 0.2	0.7 ± 0.2	0.7 ± 0.2	0.7 ± 0.2	0.8 ± 0.2	0.7 ± 0.2	0.8 ± 0.2	0.7 ± 0.2	0.7 ± 0.2	0.7 ± 0.2	0.7 ± 0.2
*Psychrobacter*^1^	0.8 ± 0.1^a^	0.2 ± 0.0^b^	0.2 ± 0.0^b^	0.2 ± 0.0^b^	0.2 ± 0.0^b^	0.2 ± 0.0^b^	0.2 ± 0.0^b^	0.2 ± 0.0^b^	0.2 ± 0.0^b^	0.2 ± 0.1^b^	0.2 ± 0.0^b^
*Clostridium sensu stricto 18*	0.1 ± 0.0	0.1 ± 0.0	0.1 ± 0.0	0.2 ± 0.0	0.2 ± 0.0	0.1 ± 0.0	0.1 ± 0.0	0.1 ± 0.0	0.1 ± 0.0	0.1 ± 0.0	0.2 ± 0.0

*Values are model estimates based on a beta distribution and represent means ± SEM*.

1 *Effect of cohort (P ≤ 0.05) as determined by one-way ANOVA; values sharing a letter are not different from each other (P > 0.05)*.

### Milk Microbiome: Community Analysis

Hierarchical clustering of the complex bacterial community structures in milk (Figure 3B) suggested that samples collected in GBR and GBU milk clustered together with high abundance of *Streptococcus*, and intermediate abundances of *Staphylococcus, Corynebacterium 1, Dyella*, and *Bacillus*. PE and SP were relatively similar, characterized by high relative abundance of *Streptococcus, Staphylococcus*, and *Rothia*, and low abundance of *Corynebacterium 1*. It is noteworthy that ETR formed an outgroup characterized by relatively high abundance of *Rhizobium* and *Achromobacter*; intermediate abundances of *Streptococcus* and *Staphylococcus*; and very little *Propionibacterium, Dyella*, and *Rothia*. Samples from GN also formed a unique clade with relatively high levels of *Lactobacillus, Klebsiella*, and *Enterococcus*.

Like feces, there was more similarity in the milk bacterial composition within a cohort than across cohorts (ANOSIM R = 0.2244, *P* = 0.001). NMDS plots were created to evaluate the similarity of cohorts, but no clear clustering was observed (Supplementary Figure 12B).

### Milk Microbiome: Core and Unique Bacteria

For milk, *Staphylococcus* and *Streptococcus* were identified as core genera, being present in 98.7 and 97.7% of all samples, respectively (Table 3; Supplementary Figure 13). However, as with feces, there were sometimes different sets of core taxa within each cohort: *Propionibacterium* was also present in 90.5% of milk from KE, *Dyella* in 94.9% of milk collected in USW, and *Corynebacterium 1* in 95.0 and 94.1% of milk samples collected in ETR and ETU, respectively. In addition, it is noteworthy that milk collected in ETR contained 8 core genera, including *Rhizobium, Brevundimonas*, and *Achromobacter*, which were present in every sample from this location. There were also several unique bacteria identified in some cohorts in milk: only milk collected in ETR contained *Acidothermus, Demequina, Flaviflexus*, and *Pediococcus*; milk from GBU uniquely contained *Chroococcidiopsis* and *Isoptericola*; and milk from GN uniquely contained *Akkermansia* and *Butyricicoccus*.

### Milk Microbiome: Diversity Measures

There was an effect of cohort on all indices of milk microbial diversity considered (*P* < 0.0001; Table 4). Milk collected in ETR had a higher richness and a higher Fisher diversity scores than milk from ETU, GN, PE, SP, SW, USC, and USW (*P* ≤ 0.0181; *P* ≤ 0.0106, respectively). The mean Shannon diversity score of milk from ETR was higher than those of milk from GN, PE, SP, SW, and USC (*P* ≤ 0.0182). Inverse Simpson diversity scores followed similar trends as Shannon diversity.

### Relationships Between Milk and Fecal Microbiomes

**Figure 5:**
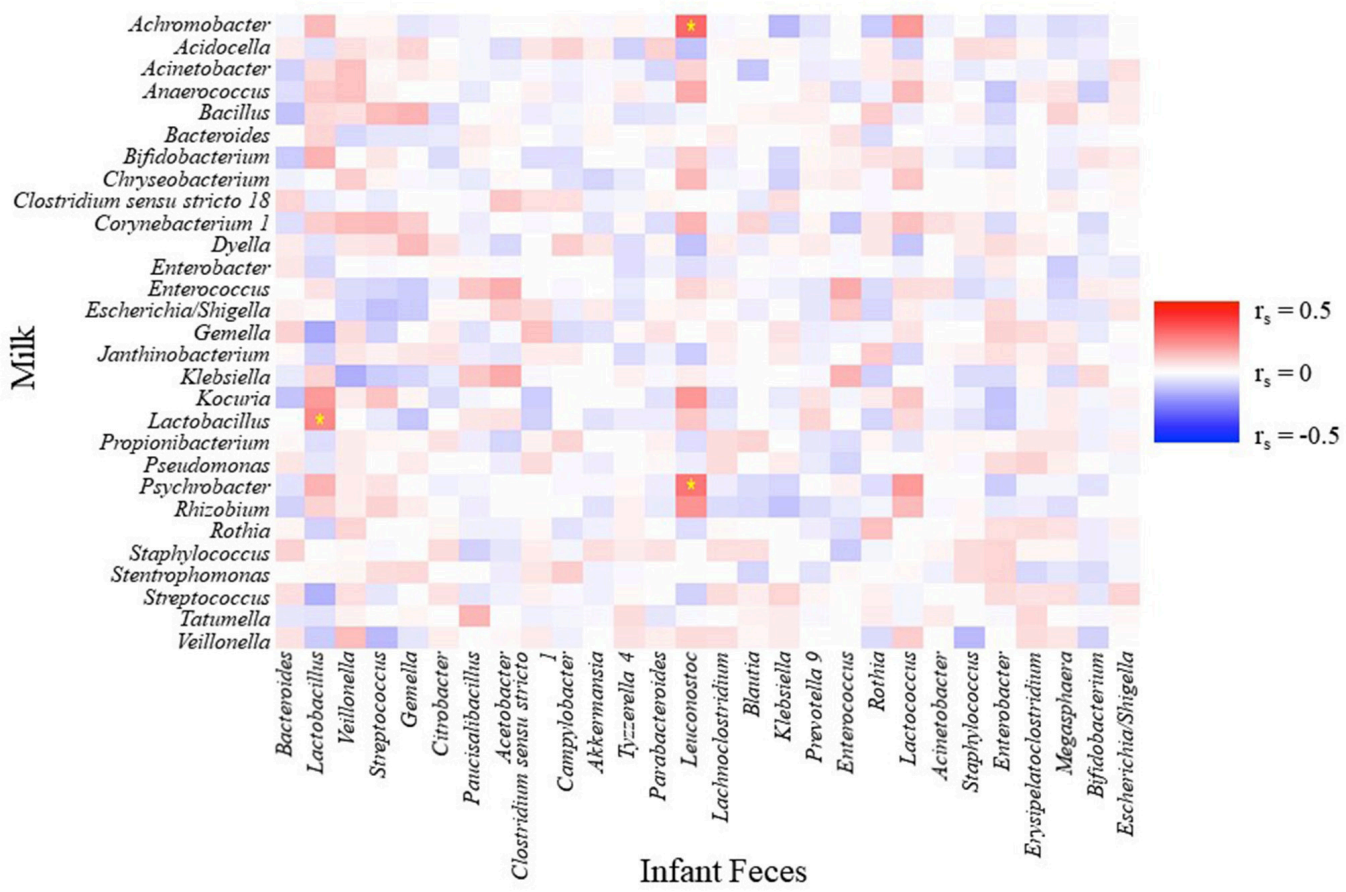
Spearman rank correlations between an aggregation of the 10 most-abundant bacterial genera in infant feces and an aggregation of the 10 most-abundant bacterial genera in milk. Stars indicate *P* < 0.01 and r_s_ < −0.3 or r_s_ > 0.3.

A few simple relationships (*P* ≤ 0.01, −0.3 ≥ r_s_ ≥ 0.3) were observed between relative abundances of the 28 aggregated genera in infant fecal and 29 aggregated genera in milk samples (Figure 5). Relative abundances of *Psychrobacter* and *Achromobacter* in milk were positively correlated with *Leuconostoc* in feces, and the relative abundance of *Lactobacillus* in milk positively correlated with *Lactobacillus* in feces.

**Figure 6:**
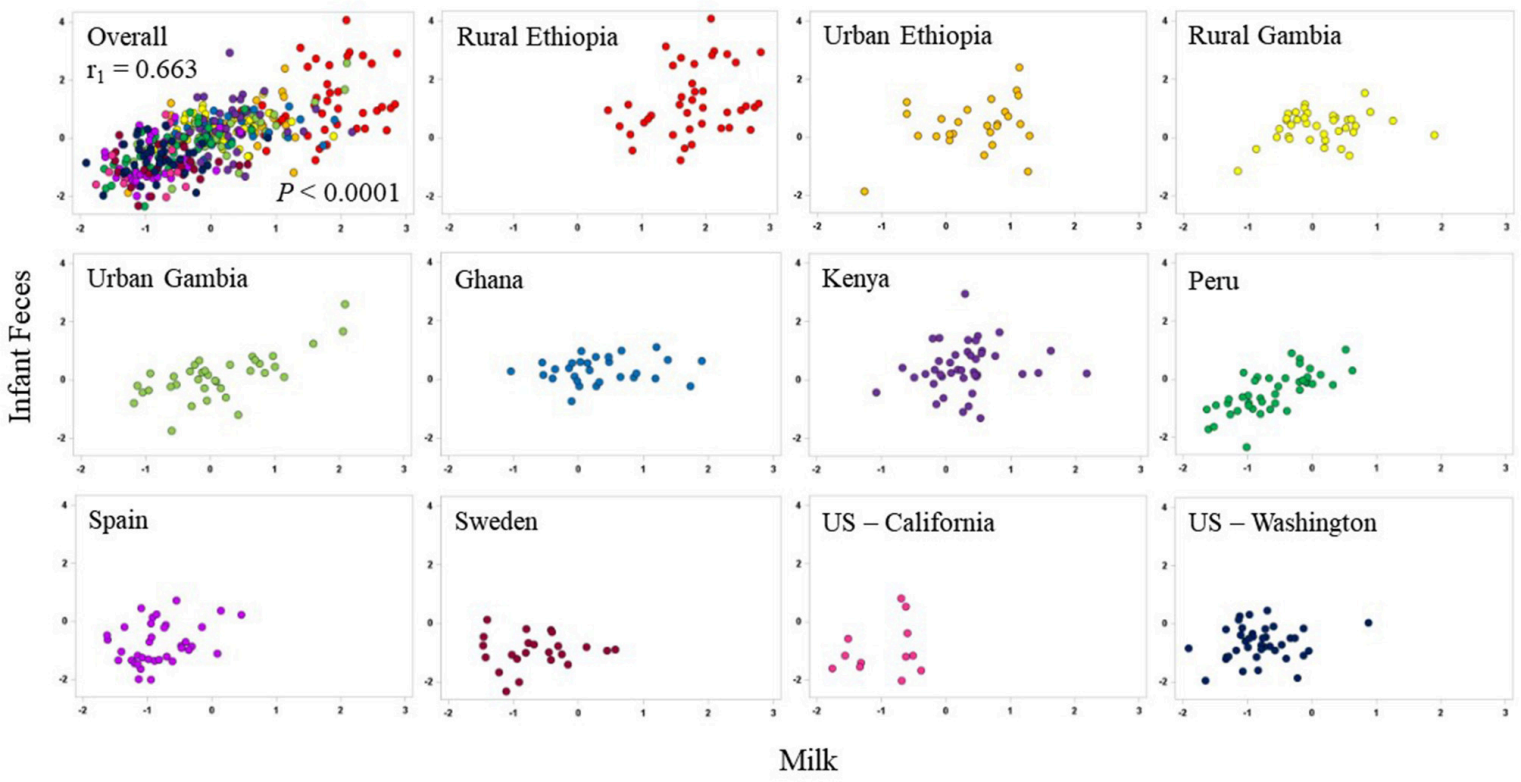
Canonical correlations between aggregations of the 10 most-abundant bacterial genera in milk (x-axes) and aggregations of the 10 most-abundant bacterial genera in infant feces (y-axes) in each cohort. The overall correlation is plotted in the upper-left panel; individual cohorts are illustrated in subsequent plots.

On a multivariate basis, canonical correlations between the aggregated milk genera and the aggregated infant fecal genera support a strong relationship between these varied and complex communities (r_1_ = 0.663 *P* < 0.0001; Figure 6). In this analysis, the first canonical component for infant feces were largely driven by *Lactobacillus* (r_s_ = 0.508) and *Leuconostoc* (r_s_ = 0.537), and the first canonical component in milk was driven by *Lactobacillus* (r_s_ = −0.498). When plotted by cohort, the relationships among the infant fecal and milk taxa appear to be specific to cohort; for example, the relationship (using the first canonical component) between milk and infant fecal microbiomes was strong in PE but diminished in SW. Correlations between milk and infant fecal diversity indices did not reveal significant correlations in any cohort except for KE where richness and Fisher diversity of infant feces and milk were positively correlated with each other (r = 0.31, *P* = 0.0453; Supplementary Table 4).

### Microbial Relationships Between Women and Their Infants

Upon evaluating the relationship between a mother's milk microbiome and her infant's fecal microbiome using both the Jaccard and Bray-Curtis distance metrics, the mother/infant dyads' samples were found to be more similar or tended to be more similar to each other than to all other combinations of mothers and infants (with Jaccard index: *P* = 0.0258; with Bray-Curtis dissimilarity: *P* = 0.0936). When evaluated within each cohort, microbial communities of a mother's milk and her infant's feces were not more similar to each other than to all other combinations of mother/infant dyads (all *P* > 0.05 for both matrices), except for in mother/infant samples from ETU in which the Bray-Curtis distance metric showed that bacterial communities between mothers and their infants were more similar to each other (*P* = 0.0185) than to other random combinations within the cohort.

## Discussion

Data from this study support our hypotheses that: (1) the human milk and infant fecal microbiomes vary among global cohorts; (2) there exists a small core group of bacteria common to milk across all cohorts (although some cohorts had additional taxa which composed their own unique core); (3) there exists a small core group of bacteria common to infant feces across all cohorts (although some cohorts had their own cores more different from the overall core); and (4) variation in the milk microbiome is related to variation in the infant fecal microbiome (although this was more apparent at the community level than the individual taxa level). Our hypothesis that fecal microbiomes of infants and the milk microbiomes of their mothers would be more similar within a cohort than to other cohorts was also supported, though the variation among both women and infants within a cohort was only slightly less than the variation among cohorts. This individual variation should be noted, because it is possible that milk and infant fecal microbiomes are tailored to a given environment. Additionally, the possibility exists that both the membership and structure of milk and fecal microbial communities may also be tailored to lifestyle and behavioral factors associated with individual maternal/infant dyads (16, 46, 47). For this reason, these results highlight the need for additional work comparing populations of women and infants globally if we are ever to understand whether a “healthy” or “normal” human milk or infant fecal microbiome exists.

Based on the summary statistics calculated from anthropometric and health data evaluated, we believe our data are representative of generally healthy women and their infants in the locations studied. However, due to the high level of individual microbial variation present among these samples, even within a cohort, these data may not be representative of neighboring populations or countries. Indeed, Meehan et al. (16) determined that among foragers and horticulturalist women in the Central African Republic who spend considerable time in in proximity to each other, milk microbial communities vary significantly both within populations and between ethnic groups. In the present study, even though Kenya and Ethiopia are geographical neighbors, substantial differences in microbial communities existed. For example, *Veillonella* and *Lactobacillus* were members of the core fecal genera in KE and ETR, but not ETU. Bacterial richness in feces from KE was greater than that from ETU, while diversity of milk from ETR and ETU were generally similar to that of KE. Additionally, milk collected from women in ETR contained relatively more *Achromobacter* and *Rhizobium* than all other sites. While these differences are not comprehensive among these cohorts, these examples illustrate that even in close geographical proximity (KE to ETR and ETU) and with genetic similarity (ETR and ETU, and GBR and GBU), there are substantial differences between milk and infant fecal microbial community structures that cannot be ignored, particularly in the framework of recommendations for healthy breastfeeding women/breastfed infants.

Our results are generally congruent with the limited number of other studies of the infant fecal microbiome among similarly-aged infants. For example, *Bifidobacterium, Streptococcus*, and the family *Enterobacteriacae*, which includes both *Enterobacter* and *Escherichia/Shigella*, were previously reported to be the most abundant taxa in feces from Gambian infants (24). Our data show that infant feces from Gambian infants also contain substantial amounts of *Lactobacillus* and *Veillonella*. Consistent with our results, Backhëd et al. (20) found *Bacteroides, Bifidobacteria, Prevotella, Streptococcus, Veillonella, and Enterobacteriacae* to be abundant in feces of Swedish infants.

It is important to note that feces collected in ETR were preserved with RNAlater, a method previously used for this purpose (48–50). However, RNAlater may introduce biases, such as an increased observation of members of the Bacteroidetes phylum, and a decrease in the members of Actinobacteria relative to unpreserved control samples; this bias has been demonstrated in soil samples and fecal samples (51–55). Despite this methodological difference, feces collected in ETR—at least with respect to phyla and the most abundant genera—were similar to those collected at its closest neighboring site, ETU. However, there were differences with respect to the core taxa, and samples from ETU formed a cluster with feces from KE and PE, while ETR was an outgroup in this clade. Further work will be needed to determine if the use of RNAlater does or does not influence bacterial communities identified in infant fecal samples collected across various cohorts.

Milk microbiomes characterized here were also relatively similar to those described in the literature previously. For instance, Kumar et al. (17) reported that relative abundance of Proteobacteria was highest in milk produced by women in South Africa; that produced by Finnish women was highest in Firmicutes; that of Chinese women had the highest *Streptococcus*; and that produced by Spanish women had the highest *Propionibacterium* and *Pseudomonas*. In our study, milk produced in ETR had the most Proteobacteria and *Rhizobium*, whereas that produced in PE had the highest level of Firmicutes, and that produced in SP the highest *Propionibacterium*. Despite these differences, most of the dominant taxa present in the study of Kumar and colleagues were also well represented in our study. Nonetheless, it remains unclear as to whether the differences in relative abundance between our present study and that of Kumar are genuine or due to methodologic differences. For example, Kumar and colleagues used the same sequencing platform, but chose primers targeting the V4 hypervariable region of the 16S rRNA gene, whereas our primers targeted the V1–V3 region. In this case, we also were only able to use the forward read, which could have been a source of bias in elucidating the microbial genera present.

Of note are the compositional differences in the ETR milk as compared to all other cohorts. Unlike all the others which were frozen upon collection, these samples were chemically preserved. The use of chemical preservatives at this site was necessary because freezing the samples was logistically difficult. While Milk Preservation Solution performed well (as compared to other preservatives) in a test of utility in preserving bacterial DNA for microbial analysis (28), the methods used for extracting the ETR samples are different than those employed on all other milk samples in this study. For this reason, results from ETR should be interpreted cautiously. For example, one notable difference we found was the high richness and diversity in the ETR milk samples as compared to the other sites. While this could be biologically relevant, this also could be an artifact of the method used for these samples.

Important for further interpretation of the findings in this study is the fact that, in addition to its microbial communities, milk's micronutrient and macronutrient compositions vary globally. Whether milk's microbiome and nutrient compositions are related has not been studied, although data from our group clearly suggests that maternal nutrient intake (which can be related to milk's nutrient composition) is related to milk microbiome (56). One example particularly relevant to the data presented here is related to human milk oligosaccharides (HMO), which vary across populations—including those reported here (26, 57). Because HMO can act as prebiotics, it is possible that variation in HMO profiles might drive variation in both milk and infant fecal microbiomes. This possibility will be explored thoroughly in subsequent publications. We have also analyzed the milk samples described in this report for their complex immune factor profiles, which also vary (25); as with HMO, subsequent publications will evaluate potential relationships among milk's immune factors, the milk microbiome, and the infant fecal microbiome. While the etiology of these population-level differences has not been completely elucidated, the potential importance of these multi-faceted differences in shaping milk's microbial communities should continue to be evaluated.

Importantly, our data provide evidence of relationships between the milk microbiome and the fecal microbiome of breastfed infants. For example, the relative abundance of *Lactobacillus* in milk was positively correlated with the relative abundance of *Lactobacillus* in infant feces. Given the fact that this genus was also determined to be the primary factor that distinguished the first and second canonical axes in our multivariate correlation analyses, it is plausible that human milk may be an important source of *Lactobacillus* for the developing infant's GI tract, as has been demonstrated through culture-dependent analyses (3–5). Future work focusing on the species and strains of *Lactobacillus* at both body sites is needed to understand these complex communities. Our data support a handful of studies published previously on this topic. For example, Murphy et al. (12) collected milk and infant fecal samples from 10 mother-infant pairs at 1, 3, 6, and 12 wk postpartum. Approximately 70–88% of the genera identified in infant feces were also identified in human milk. The most abundant genera in infant feces were *Streptococcus, Escherichia/Shigella, Bifidobacterium*, and *Veillonella*, which were the 4 most abundant genera in infant fecal samples in our study. Murphy and colleagues also observed that human milk bacterial communities exhibited greater diversity than infant fecal bacterial communities collected at the same time. With the exception of GN, more taxa were identified in milk than infant feces here as well. However, except for KE where bacterial richness of infant feces was positively correlated with that of milk, we found no correlations between the richness of milk and richness of infant feces in the populations we studied.

There are several important limitations that should be considered when interpreting the findings reported here. As previously discussed, we chemically preserved samples collected only in ETR. In addition, for practical and logistic reasons, we also used a combination of methods for collecting the milk (electric pump vs. hand expression). The impact of these methodological differences on milk's microbial composition is unknown. Additionally, limited cohort sample sizes (particularly with respect to USC), restricts the ability to conduct some statistical analyses. Furthermore, because this study is the first of its kind to this scale, we do not know if our results are comparable to other studies on milk and fecal microbial compositions, particularly as it relates to methodological differences. For example, some genera, such as *Bifidobacterium, Ruminococcus*, and *Coprococcus*, are present in higher relative abundances when mechanical lysis (via bead-beating) is used (58) due to the difficulty in cell membrane disruption for these taxa. It is important to note that characterization and analyses of the bacteria at a level lower than their genera here were not performed. Future studies should evaluate these communities at the species (and perhaps sub-species) level. The selection of a hypervariable region to target for 16S rRNA analysis is also a source of potential bias in our study (59). We chose V1–V3 because it has been previously used in our laboratory to categorize and classify the bacterial community structure of both milk and infant feces (16, 30, 56). However, future studies should be designed to determine if this hypervariable region is optimal for this application. Additionally, one important limitation to 16S-based sequencing techniques is the inability to distinguish live bacteria from dead bacteria or residual bacterial DNA that may be present in a sample. As more is understood about the microbial communities in various populations globally and at various body sites, more targeted, culture- or RNA-based approaches can be used in conjunction with 16S sequencing to more thoroughly address these questions of live contributors to the milk and infant fecal microbiomes.

Nonetheless, this research represents the largest, cross-cultural, international study to date using standardized methods of collection and analysis for characterizing the microbial compositions of human milk and infant feces. Data from this study clearly indicate that what is “normal” in terms of milk and fecal microbiomes of healthy breastfeeding mothers and their infants, respectively, varies around the world. In addition, we provide compelling evidence that the milk microbiome might, at least in part, play a role in shaping the infant's GI microbiome. It is likely that there are many additional factors that play into these findings, such as antibiotic usage, infant age, parity, infant sex, and exclusive breastfeeding status. These factors are likely important to understanding what are normal for milk and infant fecal microbial community structures. The goal of the current study was to present the microbial communities across a broad range of populations, and subsequent studies will focus on parsing the relationships among these factors and the milk and infant fecal microbiomes. For these findings to have impact, however, researchers must strive to better understand the genesis of microbial differences within and among cohorts, and if this variation is related to maternal and infant health.

In conclusion, this study is the largest of its kind to use standardized methodologies to characterize and compare the milk and infant fecal microbiomes of maternal/infant dyads worldwide. Substantial differences in both the composition and relative abundance of specific taxa were present among cohorts. We also found substantial variation among women/infants within a cohort, suggesting that environment alone does not drive variation in milk and fecal microbial community structure. Additionally, relationships present among the genera in milk and the genera in infant feces which vary among cohorts suggest that milk may be tailored not only to infants in a given environment, but also specifically to the needs of individuals. As such, we conclude that what is “normal” in terms of the fecal microbiome of healthy infants and milk microbiome of healthy lactating women varies by culture and/or location. Further, we posit that bacterial compositions of human milk and feces of breastfed infants are likely specific to a dyad within a culture and location. Future studies should evaluate the bacterial species and sub-species encompassed in these genera, their functionality, and whether or not variation is related to maternal and infant health.

## Ethics Statement

This study was carried out in accordance with the recommendations of the Washington State University Institutional Review Board and the ethics approval committees at each participating location with informed consent from all subjects. All subjects gave informed consent in accordance with the Declaration of Helsinki. The protocol was approved by the Washington State University Institutional Review Board (approval #13264).

## Author Contributions

MKM, CM, MAM, JW, JF, DS, LK, JR, LB, RP, and AP designed the study. KL, JZ, EB, CM, EK-M, EK, DK, SM, SEM, GO, CG-C, EJ, and LR collected the samples, KL, EB, and JZ performed the laboratory analyses, KL, WP, and JW performed the data analyses, KL and MKM wrote the manuscript. All authors read, contributed to, and approved the final manuscript.

### Conflict of Interest Statement

CG-C and EJ are employed by company Probisearch. The remaining authors declare that the research was conducted in the absence of any commercial or financial relationships that could be construed as a potential conflict of interest.

## Abbreviations

ANOSIM: analysis of similarity
ANOVA: analysis of variance
GI: gastrointestinal
GLIMMIX: generalized linear mixed model
HMO: human milk oligosaccharide
ASV: amplicon sequence variants
ETR, Ethiopia: rural site
ETU, Ethiopia: urban site
GBR, The Gambia: rural site
GBU, The Gambia: urban site
GN: Ghana
KE: Kenya
PE: Peru
SP: Spain
SW: Sweden
USC, United States: California site
USW, United States: Washington site.
