# Corrigendum: What's Normal? Microbiomes in Human Milk and Infant Feces Are Related to Each Other but Vary Geographically: The INSPIRE Study

**DOI:** 10.3389/fnut.2020.00012

**Published:** 2020-02-19

**Authors:** Kimberly A. Lackey, Janet E. Williams, Courtney L. Meehan, Jessica A. Zachek, Elizabeth D. Benda, William J. Price, James A. Foster, Daniel W. Sellen, Elizabeth W. Kamau-Mbuthia, Egidioh W. Kamundia, Samwel Mbugua, Sophie E. Moore, Andrew M. Prentice, Debela Gindola K., Linda J. Kvist, Gloria E. Otoo, Cristina García-Carral, Esther Jiménez, Lorena Ruiz, Juan M. Rodríguez, Rossina G. Pareja, Lars Bode, Mark A. McGuire, Michelle K. McGuire

**Affiliations:** ^1^Margaret Ritchie School of Family and Consumer Sciences, University of Idaho, Moscow, ID, United States; ^2^Department of Animal and Veterinary Science, University of Idaho, Moscow, ID, United States; ^3^Department of Anthropology, Washington State University, Pullman, WA, United States; ^4^Statistical Programs, College of Agricultural and Life Sciences, University of Idaho, Moscow, ID, United States; ^5^Department of Biological Sciences, University of Idaho, Moscow, ID, United States; ^6^Dalla Lana School of Public Health, University of Toronto, Toronto, ON, Canada; ^7^Department of Human Nutrition, Egerton University, Nakuru, Kenya; ^8^Department of Women and Children's Health, King's College London, London, United Kingdom; ^9^MRC Unit The Gambia at the London School of Hygiene and Tropical Medicine, Fajara, Gambia; ^10^MRC International Nutrition Group, London School of Hygiene and Tropical Medicine, London, United Kingdom; ^11^Department of Anthropology, Hawassa University, Hawassa, Ethiopia; ^12^Faculty of Medicine, Lund University, Lund, Sweden; ^13^Department of Nutrition and Food Science, University of Ghana, Accra, Ghana; ^14^Probisearch, Tres Cantos, Spain; ^15^Department of Microbiology and Biochemistry of Dairy Products, Instituto de Productos Lácteos de Asturias (IPLA-CSIC), Villaviciosa, Spain; ^16^Department of Nutrition, Food Science, and Food Technology, Complutense University of Madrid, Madrid, Spain; ^17^Nutrition Research Institute, Lima, Peru; ^18^Larsson-Rosenquist Foundation Mother-Milk-Infant Center of Research Excellence, University of California, San Diego, La Jolla, CA, United States; ^19^Department of Pediatrics, University of California, San Diego, La Jolla, CA, United States

**Keywords:** human milk, breastmilk, feces, microbiome, international, infant, breastfeeding, maternal

In the original article, there was an error. The volume of milk subjected to DNA extraction was reported as 2.5 mL, which is an error in the decimal placement. The actual volume of milk used was 0.25 mL.

A correction has been made to the **Materials and Methods** section, subsection **Extraction of DNA from Milk, paragraph 2**:

“DNA in **0.25 mL** of each milk sample collected in ETR was extracted using the kit accompanying the Milk Preservation Solution (Norgen Biotek, Thorold, Ontario) as per manufacturer's instructions, including the 2 h enzymatic lysis (20 mg/mL lysozyme). DNA was eluted in 100 μL elution buffer (included with the kit) and stored at −20°C until amplification. Nuclease-free water (500 μL; Ambion) was extracted as a negative control.”

Additionally, the reports of the sequencing reads in the results section were incorrect. A correction has been made to the **Results** section, subsection **Sequencing Summary**:

“The sequencing run for the 398 infant fecal samples generated **4,385,982** reads, with a mean (± standard deviation, SD) of **11,020** ± **6,632** reads and a range of **10** to 40,267 reads following initial processing using the DADA2 workflow. After additional filtering of any read that could not be classified to the genus level, and omitting any sample with <1,000 reads, the infant fecal dataset analyzed here contained **4,314,551** reads across 377 samples, with a mean (± SD) of **11,444** ± **6,198**; and a range of **1,662**–**40,255** reads. For the 409 milk samples, sequencing generated **7,528,193** reads, with a mean (± SD) of **18,406** ± **18,389** reads and a range of **12**−141,620 reads following initial processing using the DADA2 workflow. Using the same filtering criteria as the infant fecal dataset, the milk dataset used here contained 6,709,277 reads across 394 samples, with mean (± SD) of 17,029 ± 16,783, and a range of 1,302–130,700 reads. These curated datasets were used for all further analyses.”

The authors apologize for this error and state that this does not change the scientific conclusions of the article in any way. The original article has been updated.

