# Supplementary material for: What's Normal? Microbiomes in Human Milk and Infant Feces Are Related to Each Other but Vary Geographically: The INSPIRE Study
Source: Front Nutr. 2019 Apr 17;6:45. doi: 10.3389/fnut.2019.00045 (PMC6479015; doi:10.3389/fnut.2019.00045)
Supplement: Supplementary file 1 [file Table_1.docx]

| Supplementary Table 1. Chronological summary of previously published research characterizing microbial communities in human milk using high-throughput sequencing. | | | | |
| --- | --- | --- | --- | --- |
| Reference | **Location and Population** | **Most Abundant Taxa** | **Sequencing Methods** | **Additional Notes** |
| Hunt et al., 2011 | United States (Pacific Northwest); 22-26 wk postpartum; healthy women; *n* = 16 | *Streptococcus*, *Staphylococcus*, *Serratia*, *Pseudomonas* | 454-pyrosequencing, V1-V2 | Repeated samples at 3 times. Identified 9 “core” genera. |
| Cabrera-Rubio et al., 2012 | Finland; 0-2 d, 1 mo, 6 mo postpartum; healthy women; *n* = 18 | Colostrum: *Weisella, Leuconostoc, Staphylococcus, Streptococcus,* and *Lactococcus*  1 and 6 mo: *Veillonella*, *Leptotrichia*, and *Prevotella* | 454-pyrosequencing, V1-V3 | Differences found in microbiomes related to other factors, such as delivery method. |
| Jost et al., 2013 | Switzerland; 3-6 d, 9-14 d, 25-30 d postpartum; healthy women; *n* = 7 | *Staphylococcus*, *Streptococcus*, *Propionibacterium, Bifidobacterium,* and *Veillonella* | Sanger sequencing and 454-pyrosequencing, V5-V6 |  |
| González et al., 2013 | Mozambique; ≤ 2 wk-12 mo postpartum; women with HIV (*n* = 29) and women without HIV (*n* = 92) | *Streptococcus, Staphylococci, Enterococci, Bifidobacterium,* and lactic acid bacteria groups | PCR sequencing of a 470 pb fragment (unspecified) of the 16S RNA gene | Milk microbiomes of women with HIV RNA in their milk were different than milk microbiomes of women without HIV RNA in their milk. |
| Urbaniak et al., 2014 | Canada; time postpartum not reported; healthy women (*n* = 8) and a woman with non-Hodgkin’s lymphoma undergoing chemotherapy | Healthy women: *Acinetobacter*, *Staphylococcus*, *Cloacabacterium*, and *Gammaproteobacteria*  Non-Hodgkin’s woman: *Acinetobacter*, *Xanthomonadaceae*, *Stenotrophomonas* | Ion torrent sequencing, V6 | Milk microbiomes less diverse in woman on chemotherapy than in healthy controls. |
| Jiménez et al., 2015 | Spain; time postpartum not reported; healthy women (*n* = 10) and women with mastitis (*n* = 10) | Healthy women: *Staphylococcus,*  *Streptococcus, Bacteroides, Faecalibacterium, Ruminococcus, Lactobacillus*, and *Propionibacterium*  Women with mastitis: *Staphylococcus* | 454-pyrosequencing  metagenome analysis (archaea, viral, fungal and protozoan sequences were also detected and analyzed) | Compared to healthy women, milk microbiomes in women with mastitis had lower diversity. |
| Davé et al., 2016 | United States (California); 2-4 d postpartum; healthy (albeit obese) Mexican-American women; *n* = 10 | *Staphylococcus*, *Streptococcus*, *Xanthomonadaceae, Sediminibacterium* | Illumina sequencing, V4 | Also analyzed children’s saliva 5 yr after milk was collected. |
| Urbaniak et al., 2016 | Canada; 6+ d postpartum; healthy women; *n* = 39 | *Streptococcus*, *Pseudomonas*, *Staphylococcus*, and *Lactobacillus* | Illumina sequencing, V6 region | No differences found in milk microbiomes between cesarean and vaginal delivery or pre- and term births. |
| Sakwinska et al., 2016 | Beijing (China); 0-4 d, 5-11 d, or 1-2 mo postpartum; women not cleaning breast (*n* = 30) and women cleaning breast (*n* = 60) | Cleaned breast: *Streptococci, Staphylococci* Uncleaned breast*: Streptococci, Staphylococci,* and *Acinetobacter* | qPCR targeting 16S rRNA gene, hypervariable region unspecified | Higher bacterial counts found in the milk collected from uncleaned breasts. |
| Cabrera-Rubio et al., 2016 | Spain; 1 mo postpartum; healthy women; vaginal delivery (*n* = 6) and cesarean delivery (*n* = 4) | *Streptococcus, Staphylococcus, Leuconostocaceae, Lactobacillaceae, Veillonellaceae, Flavobacteriaceae* | 16S pyrosequencing, V1-V3 region | Findings suggest that delivery mode does not impact milk microbiome. |
| Boix-Amorós et al., 2016 | Spain; 5 d, 6-15 d, 15+ d postpartum; healthy women; *n* = 21 | *Staphylococcus*, *Pseudomonas*, *Streptococcus*, *Acinetobacter* | 16S rRNA pyrosequencing, 8F and 785R | Milk bacterial communities personalized to an individual. |
| Kumar et al., 2016 | Spain, Finland, South Africa, China; 1 mo postpartum; healthy women; *N* = 80, 20 from each country | *Staphylococcus*, *Streptococcus*, *Pseudomonas*, *Ralstonia*, *Acinetobacter* | 16S rRNA sequencing, V4 | Milk microbial profiles differed by location and delivery mode. |
| Patel et al., 2017 | India; time postpartum not reported; healthy women (*n* = 18) and women with mastitis (*n* = 32) | Milk from healthy controls had relatively more *Acinetobacter*, *Ruminococcus*, *Clostridium*, and *Eubacterium* than that from women with mastitis. | Ion torrent sequencing, V2-V3 | Compared to healthy women, those with mastitis produced milk with lower bacterial diversity. |
| Murphy et al., 2017 | Ireland; 1, 3, 6, and 12 wk postpartum; healthy women; *n* = 10 | *Streptococcus*, *Pseudomonas*, *Staphylococcus*, *Elizabethkingia* | Illumina sequencing, V3-V4 |  |
| Li et al., 2017 | China (*n* = 102); Taiwan (*n* = 31); 0.1-21.7 mo postpartum; healthy women | *Streptococcaceae*, *Pseudomonadaceae*, *Staphylococcaceae*, *Lactobacillaceae*, *Oxalobacteraceae* | Pyrotag sequencing, V1-V2 | A greater number of unique OTUs was found in milk produced by women who delivered by cesarean section. |
| Pannaraj et al., 2017 | California, Florida (United States); 1-331 d postpartum; healthy women; *n* = 107 | *Streptococcaceae*, *Staphylococcaceae*, *Pseudomonadaceae* | Illumina sequencing, V4 | Also collected maternal skin (areolar) swabs and infant feces. |
| Vaidya et al., 2017 | India; 15-90 d postpartum; healthy women; urban (*n* = 15) and rural (*n* = 15) | *Pseudomonas, Enterobacter, Ralstonia* | Ion torrent sequencing, V2-V3 |  |
| Williams et al., 2017 | United States (Pacific Northwest); 9 time points from 0 d-6 mo postpartum; healthy women; *n* = 21 | *Streptococcus, Staphylococcus, Gemella, Veillonella, Ralstonia* | Illumina sequencing, V1-V3 | Also collected maternal feces (Carrothers et al., 2015), infant feces, maternal saliva, and infant saliva (Williams et al., in press). |
| Toscano 2017 | Italy; 0-3 d postpartum; vaginal deliveries (*n* = 15) and cesarean deliveries (*n* = 14) | *Streptococcus, Staphylococcus* | Ion torrent sequencing, V2–4–8 and V3–6, 7–9 | Delivery mode associated with differences in microbiomes of colostrum. |
| Biagi et al., 2017 | Italy; 20 d postpartum; relatively healthy women; *n* = 36 | *Carnobacteriaceae, Paenibacillaceae, Bifidobacteriaceae,* and *Lachnospiraceae* | Illumina sequencing, V3-V4 | Also collected infant oral samples and feces. |
| Drago et al., 2017 | Italy (Verona, *n* = 20) and Burundi (*n* = 30); 0-3 d, 1 mo postpartum; healthy women | Most abundant taxa not interpretable. | Ion torrent sequencing, V2–4–8 and V3–6, 7–9 |  |
| Drell et al., 2017 | Estonia; 48–72 h, 6–8 wk, and 6 mo postpartum; healthy women; *n* = 7 | *Streptococcus, Staphylococcus, Propionibacterium, Gemella, Acinetobacter,* and *Enterococcus* | Illumina sequencing, V1-V2 | Also looked at maternal rectal, vaginal, oral cavity, and mammary areolar and infant oral and fecal microbiomes. |
| Chen et al., 2018 | Taiwan; 1-33 d postpartum; healthy women; *n* = 33 | *Staphylococcus, Streptococcus, Enhydrobacter, Enterococcus, Rothia* | Illumina sequencing, V3-V4 | Colostrum and transitional milk had different microbial profiles. |
| Meehan et al., 2018 | Central African Republic; time postpartum range not reported; hunter-gatherer mothers (*n* = 27) and horticulturalist mothers (*n* = 14) | *Streptococcus, Staphylococcus, Veillonella, Corynebacterium,* and *Rhodococcus* | Illumina sequencing, V1-V3 | Differences found between ethnic groups; potential influence of season and childcare practices. |
| Biagi et al., 2018 | Italy; 0, 4, 7, 14, 21, and 30 d postpartum; healthy women with moderately preterm infants; *n* = 16 | *Streptococcaceae, Staphylococcacae, Corynebacteriaceae,* and *Bifidobacteraceae* | Illumina sequencing, V3-V4 | Infants were singletons (*n* = 11) and twins (*n* = 5). Also collected infant saliva and stool. 3 “milk types” of microbiomes identified. |
| Tuominen et al., 2018 | Finland; 0 d (*n* = 31) and 2 mo postpartum (*n* = 4); mothers with (*n* = 3) and without human papilloma virus (*n* = 32) | *Streptococcaceae, Gemellaceae,* and *Staphylococcaeae* | Illumina sequencing, V3-V4 | Also collected infant saliva. HPV infection associated with differences in oral microbial communities but not milk microbial communities. |

| \| **Supplementary Table 2**. Effect of cohort on relative abundances (%) of bacterial phyla in infant feces. Values are model estimates based on a beta distribution and represent means ± SEM. \| \| --- \| | | | | | | | | | | | |
| --- | --- | --- | --- | --- | --- | --- | --- | --- | --- | --- | --- | --- |
| **Phylum** | **Ethiopia Rural** | **Ethiopia Urban** | **The Gambia Rural** | **The Gambia Urban** | **Ghana** | **Kenya** | **Peru** | **Spain** | **Sweden** | **US**  **California** | **US**  **Washington** |
|  | *n* = 40 | *n* = 32 | *n* = 38 | *n* = 38 | *n* = 32 | *n* = 42 | *n* = 42 | *n* = 37 | *n* = 23 | *n* = 12 | *n* = 41 |
| Firmicutes^1^ | 66.7 ± 3.8^a^ | 51.5 ± 4.7^abc^ | 58.3 ± 4.2^ab^ | 58.3 ± 4.2^ab^ | 45.7 ± 4.7^bc^ | 55.4 ± 4.0^abc^ | 52.9 ± 4.1^abc^ | 36.9 ± 4.1^c^ | 44.6 ± 5.5^abc^ | 52.0 ± 7.6^abc^ | 42.8 ± 4.1^bc^ |
| Proteobacteria | 19.1 ± 2.6 | 21.3 ± 3.1 | 16.8 ± 2.4 | 19.0 ± 2.7 | 24.2 ± 3.4 | 22.4 ± 2.8 | 25.1 ± 3.0 | 29.8 ± 3.5 | 27.0 ± 4.3 | 14.4 ± 3.8 | 22.2 ± 2.8 |
| Bacteroidetes | 11.2 ± 1.9 | 11.7 ± 2.2 | 10.1 ± 1.7 | 12.5 ± 2.1 | 10.8 ± 2.0 | 13.9 ± 2.2 | 12.2 ± 2.0 | 14.6 ± 2.5 | 18.4 ± 3.7 | 18.2 ± 5.0 | 20.4 ± 3.1 |
| Actinobacteria^1^ | 8.7 ± 1.4^c^ | 11.3 ± 2.0^abc^ | 16.6 ± 2.5^abc^ | 17.8 ± 2.7^ab^ | 22.0 ± 3.4^a^ | 10.6 ± 1.7^bc^ | 9.6 ± 1.5^bc^ | 12.6 ± 2.1^abc^ | 10.9 ± 2.3^abc^ | 6.1 ± 1.8^c^ | 11.8 ± 1.9^abc^ |
| Verrucomicrobia | 0.5 ± 0.1 | 0.5 ± 0.1 | 0.5 ± 0.1 | 0.5 ± 0.1 | 0.5 ± 0.1 | 0.5 ± 0.1 | 0.5 ± 0.1 | 0.5 ± 0.1 | 0.5 ± 0.1 | 0.5 ± 0.1 | 0.5 ± 0.1 |
| ^1^Effect of cohort (*P* ≤ 0.05) as determined by one-way ANOVA; values sharing a letter are not different from each other (*P* > 0.05). | | | | | | | | | | | |

| \| **Supplementary Table 3**. Effect of cohort on relative abundances (%) of bacterial phyla in milk. Values are model-based estimates based on a beta distribution and represent means ± SEM. \| \| --- \| | | | | | | | | | | | |
| --- | --- | --- | --- | --- | --- | --- | --- | --- | --- | --- | --- | --- |
| **Phylum** | **Ethiopia Rural** | **Ethiopia Urban** | **The Gambia Rural** | **The Gambia Urban** | **Ghana** | **Kenya** | **Peru** | **Spain** | **Sweden** | **US**  **California** | **US**  **Washington** |
|  | *n* = 40 | *n* = 34 | *n* = 39 | *n* = 38 | *n* = 38 | *n* = 42 | *n* = 43 | *n* = 40 | *n* = 24 | *n* = 19 | *n* = 39 |
| Firmicutes^1^ | 32.6 ± 3.7^c^ | 60.4 ± 4.4^ab^ | 51.9 ± 4.3^bc^ | 54.3 ± 4.3^b^ | 66.2 ± 4.0^ab^ | 60.0 ± 4.0^ab^ | 73.8 ± 3.2^a^ | 65.8 ± 3.8^ab^ | 68.7 ± 4.8^ab^ | 55.9 ± 6.0^abc^ | 54.3 ± 4.2^b^ |
| Proteobacteria^1^ | 46.9 ± 4.0^a^ | 14.7 ± 2.3^b^ | 23.3 ± 3.0^b^ | 23.6 ± 3.1^b^ | 23.1 ± 3.1^b^ | 19.3 ± 2.6^b^ | 14.2 ± 2.0^b^ | 21.3 ± 2.8^b^ | 15.4 ± 3.0^b^ | 22.6 ± 4.2^b^ | 25.1 ± 3.2^b^ |
| Actinobacteria^1^ | 24.1 ± 2.6^ab^ | 27.8 ± 3.1^a^ | 24.7 ± 2.7^ab^ | 23.6 ± 2.7^ab^ | 5.1 ± 0.8^d^ | 17.4 ± 2.1^abc^ | 11.2 ± 1.5^c^ | 10.4 ± 1.5^c^ | 13.2 ± 2.4^bc^ | 8.1 ± 1.8^cd^ | 20.9 ± 2.5^ab^ |
| Bacteroidetes^1^ | 2.4 ± 0.4^ab^ | 1.5 ± 0.3^ab^ | 2.0 ± 0.3^ab^ | 1.7 ± 0.3^ab^ | 1.2 ± 0.2^b^ | 2.8 ± 0.4^a^ | 1.8 ± 0.3^ab^ | 1.8 ± 0.3^ab^ | 2.1 ± 0.4^ab^ | 1.2 ± 0.3^ab^ | 2.4 ± 0.4^ab^ |
| Other^1^ | 1.3 ± 0.2^cd^ | 1.2 ± 0.2^cd^ | 2.7 ± 0.4^a^ | 2.4 ± 0.3^ab^ | 0.6 ± 0.1^d^ | 1.4 ± 0.2^bce^ | 0.7 ± 0.1^d^ | 0.9 ± 0.1^cd^ | 0.8 ± 0.2^cd^ | 0.7 ± 0.2^de^ | 1.5 ± 0.2^abce^ |
| ^1^Effect of cohort (*P* ≤ 0.05) as determined by one-way ANOVA; values sharing a letter are not different from each other (*P* > 0.05). | | | | | | | | | | | |

| Supplementary Table 4. Spearman rank correlations between milk and infant fecal microbial diversity indices (rarefied to 1000 reads). | | | |
| --- | --- | --- | --- |
| Cohort | **Richness/Fisher Diversity^1^** | **Shannon Diversity** | **Inverse Simpson Diversity** |
| Ethiopia Rural | r = 0.16 | r = 0.08 | r = 0.01 |
|  | *P* = 0.3129 | *P* = 0.6305 | *P* = 0.9597 |
| Ethiopia Urban | r = -0.01 | r = -0.08 | r = -0.11 |
|  | *P* = 0.9616 | *P* = 0.7137 | *P* = 0.5912 |
| The Gambia Rural | r = -0.10 | r = -0.06 | r = 0.06 |
|  | *P* = 0.5471 | *P* = 0.7464 | *P* = 0.7380 |
| The Gambia Urban | r = 0.06 | r = -0.08 | r = -0.13 |
|  | *P* = 0.7326 | *P* = 0.6234 | *P* = 0.4673 |
| Ghana | r = 0.16 | r = -0.11 | r = -0.15 |
|  | *P* = 0.4310 | *P* = 0.5811 | *P* = 0.4581 |
| Kenya | **r = 0.32** | r = 0.05 | r = -0.08 |
|  | ***P* = 0.0359** | *P* = 0.7485 | *P* = 0.6060 |
| Peru | r = -0.09 | r = -0.00 | r = -0.02 |
|  | *P* = 0.5563 | *P* = 0.9825 | *P* = 0.8830 |
| Spain | r = 0.29 | r = 0.30 | r = 0.30 |
|  | *P* = 0.0880 | *P* = 0.0799 | *P* = 0.0748 |
| Sweden | r = -0.19 | r = -0.42 | r = -0.31 |
|  | *P* = 0.3959 | *P* = 0.0541 | *P* = 0.1619 |
| US California | r = -0.15 | r = -0.41 | r = -0.45 |
|  | *P* = 0.6476 | *P* = 0.1826 | *P* = 0.1377 |
| US Washington | r = 0.23 | r = 0.17 | r = 0.13 |
|  | *P* = 0.1618 | *P* = 0.2966 | *P* = 0.4304 |
| ^1^Due to computational similarities and the use of Spearman’s rank correlation statistic, the correlations and associated *P*-values for richness and Fisher diversity are identical and are therefore reported here in a single column. | | | |
