# Supplementary material for: What's Normal? Microbiomes in Human Milk and Infant Feces Are Related to Each Other but Vary Geographically: The INSPIRE Study
Source: Front Nutr. 2019 Apr 17;6:45. doi: 10.3389/fnut.2019.00045 (PMC6479015; doi:10.3389/fnut.2019.00045)

Supplementary Figure 1. Relative abundance of the 30 most-abundant genera in infant fecal samples (*n* = 40) collected in rural Ethiopia. Each bar represents a single sample.

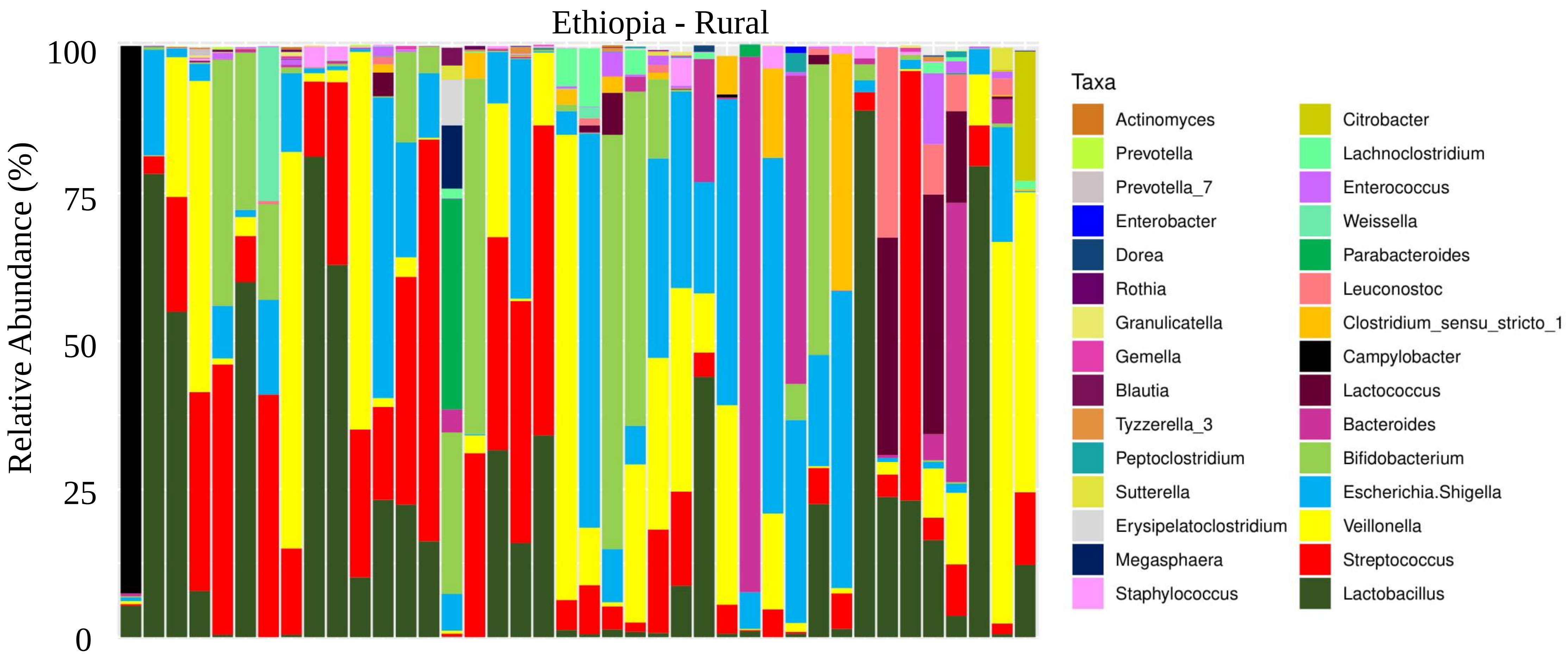

Supplementary Figure 2. Relative abundance of the 30 most-abundant genera in infant fecal samples (*n* = 32) collected in urban Ethiopia. Each bar represents a single sample.

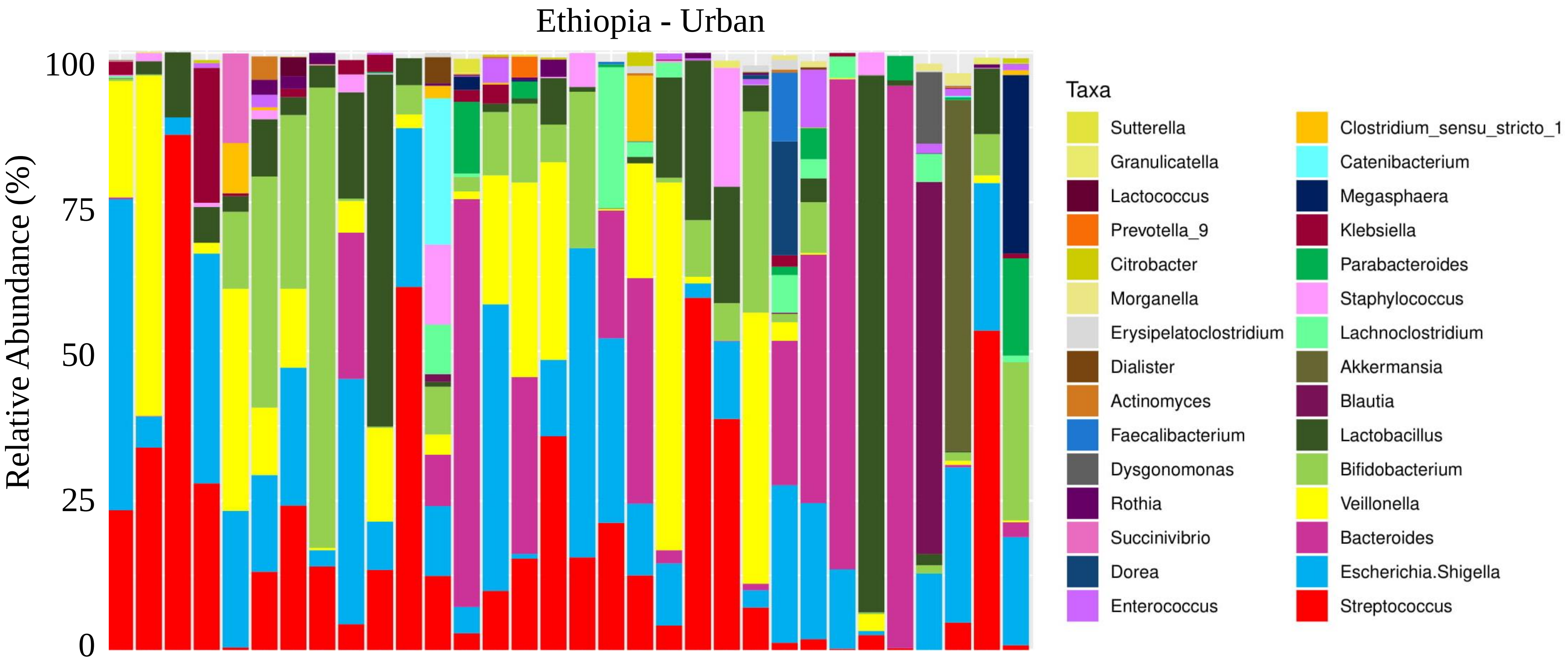

Supplementary Figure 3. Relative abundance of the 30 most-abundant genera in infant fecal samples (*n* = 38) collected in rural Gambia. Each bar represents a single sample.

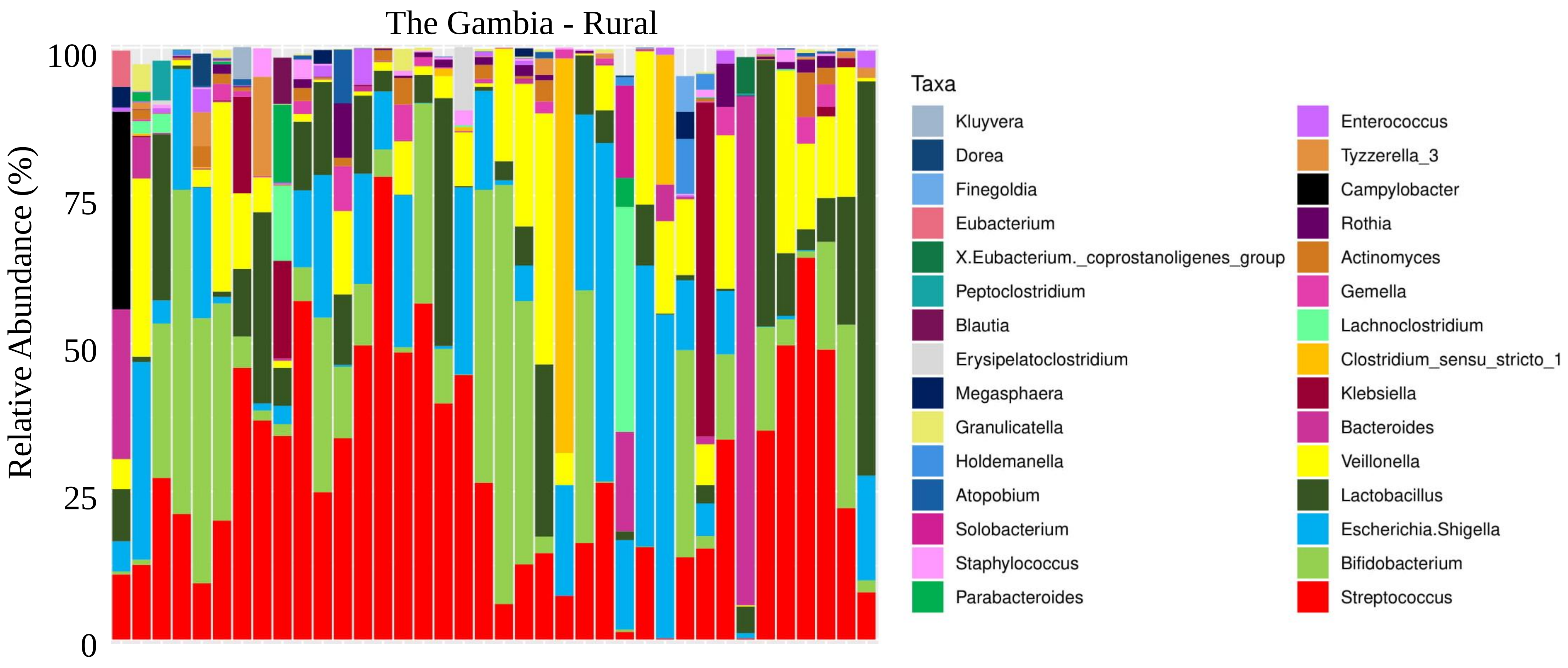

Supplementary Figure 4. Relative abundance of the 30 most-abundant genera in infant fecal samples (n = 38) collected in urban Gambia. Each bar represents a single sample.

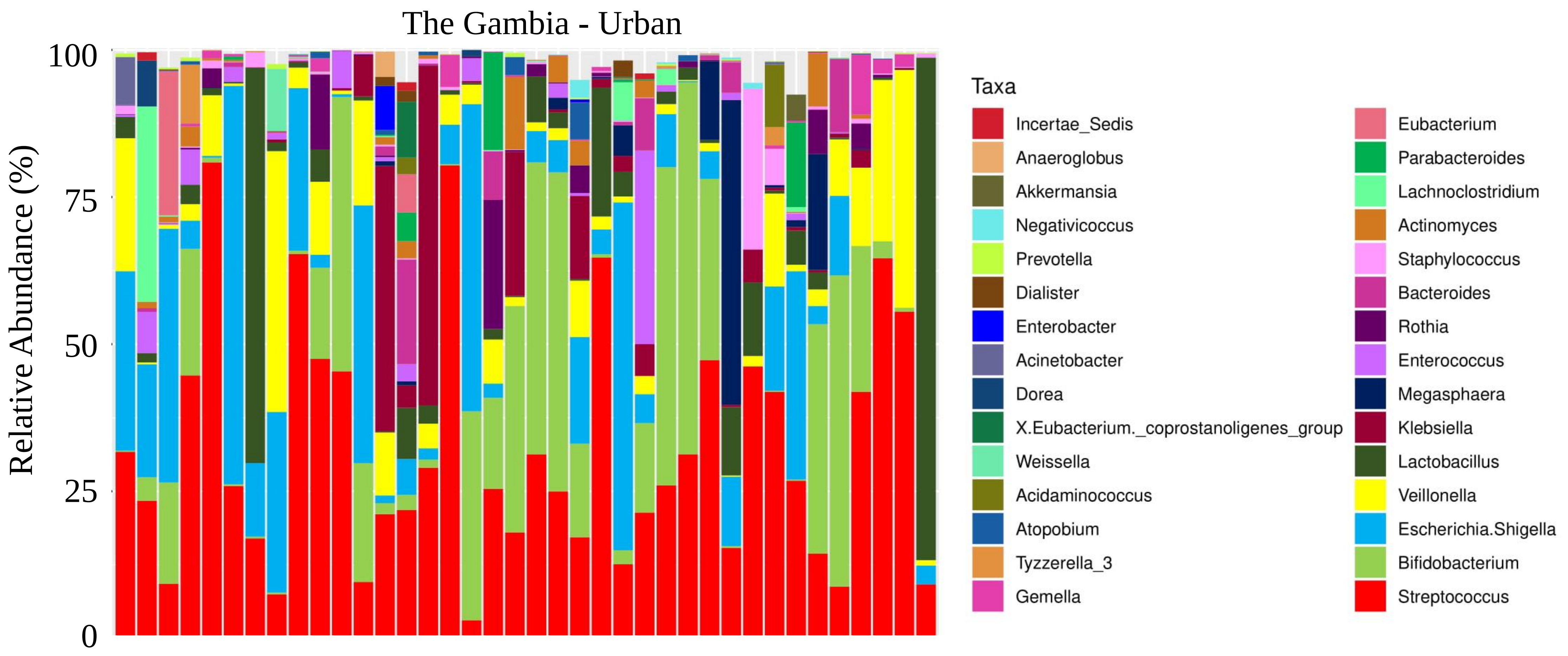

Supplementary Figure 5. Relative abundance of the 30 most-abundant genera in infant fecal samples (n = 32) collected in Ghana. Each bar represents a single sample.

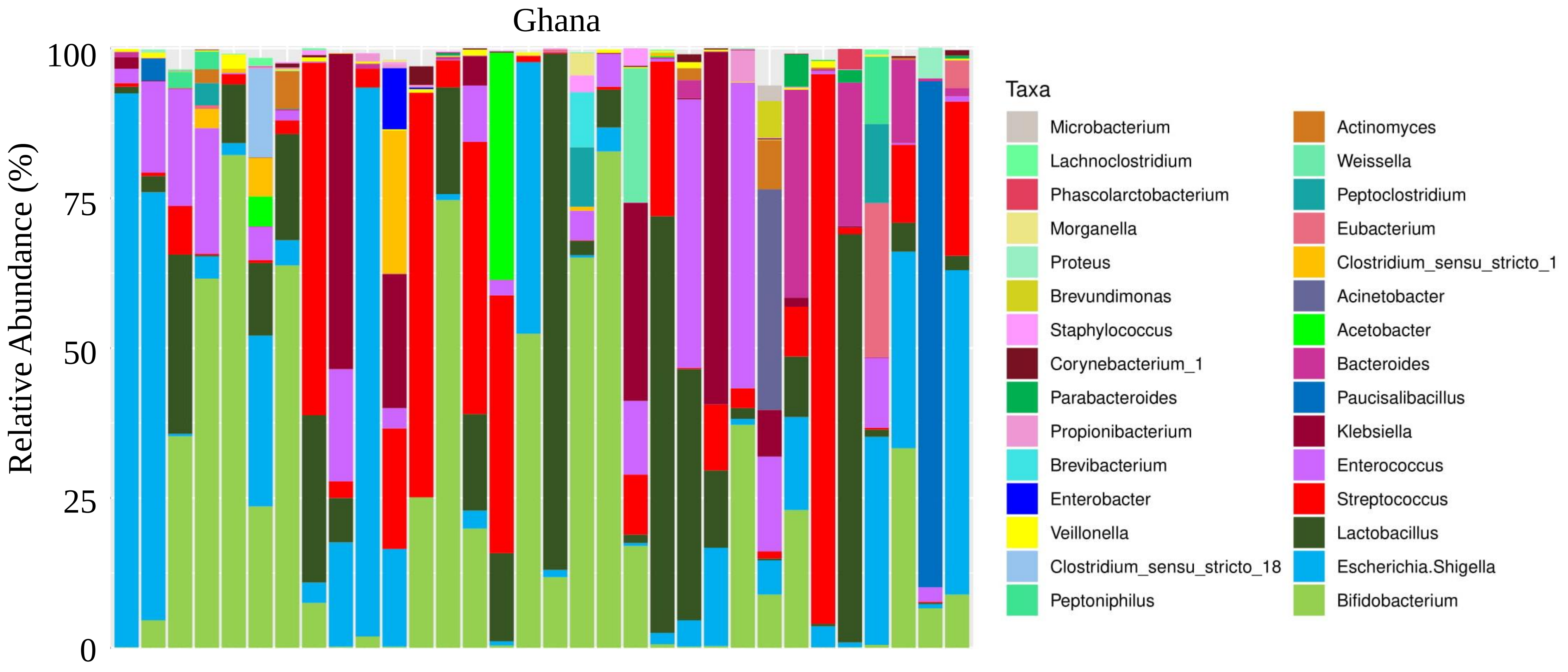

Supplementary Figure 6. Relative abundance of the 30 most-abundant genera in infant fecal samples (*n* = 42) collected in Peru. Each bar represents a single sample.

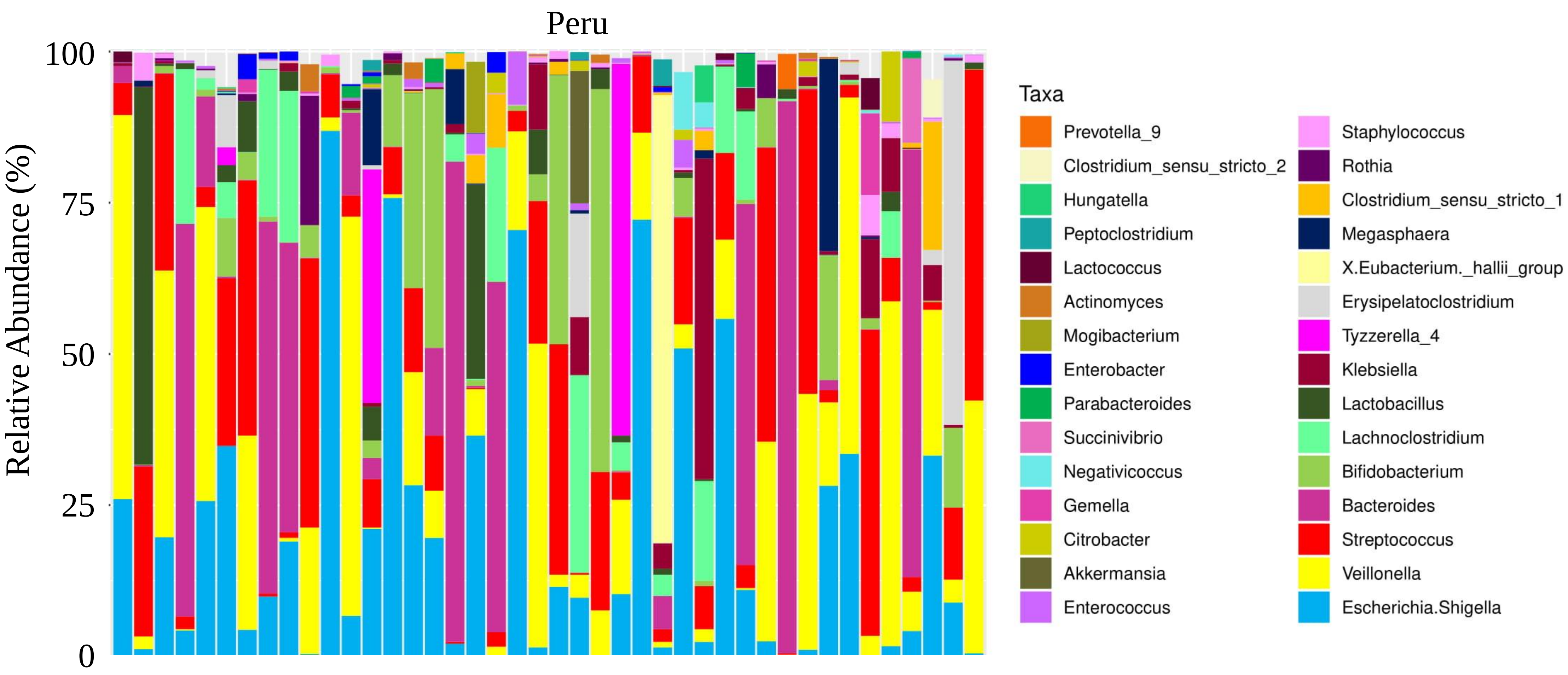

Supplementary Figure 7. Relative abundance of the 30 most-abundant genera in infant fecal samples (*n* = 42) collected in Kenya. Each bar represents a single sample.

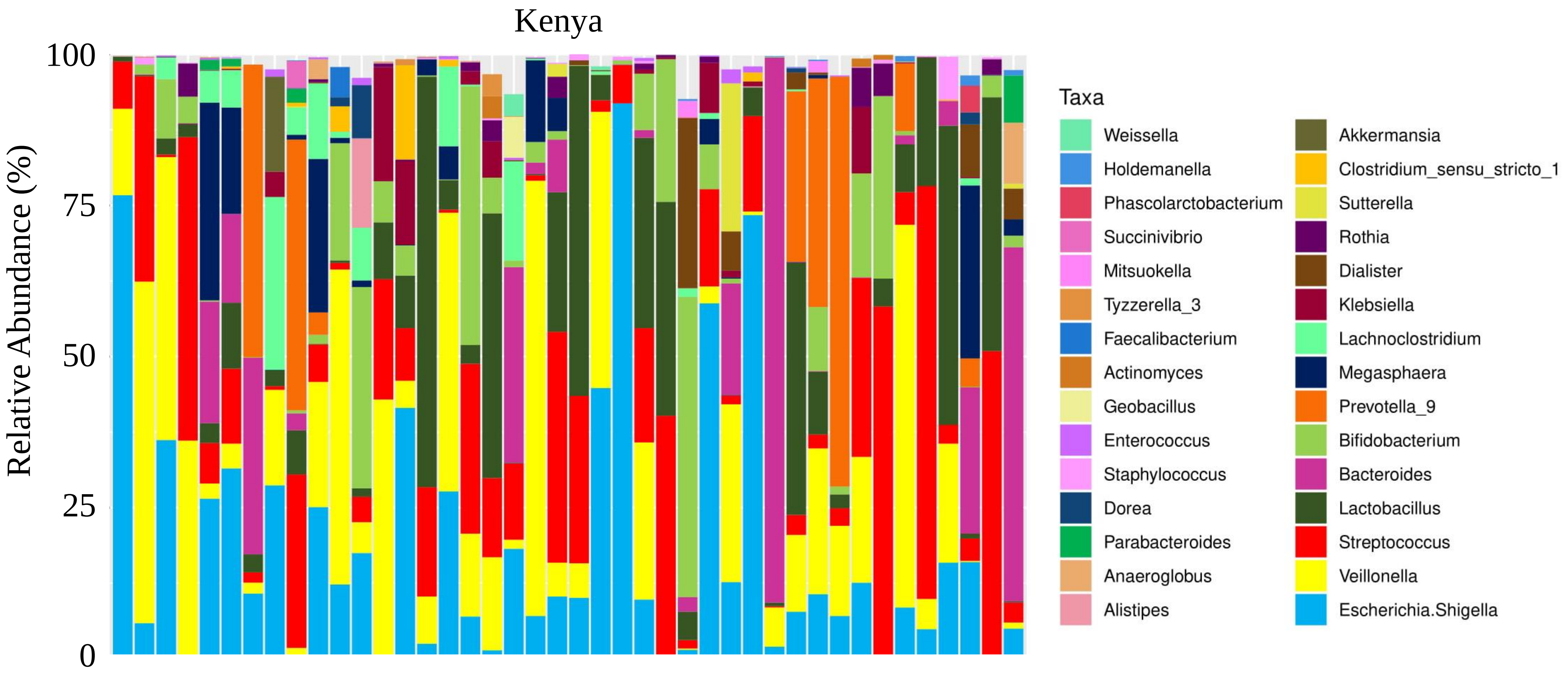

Supplementary Figure 8. Relative abundance of the 30 most-abundant genera in infant fecal samples (*n* = 37) collected in Spain. Each bar represents a single sample.

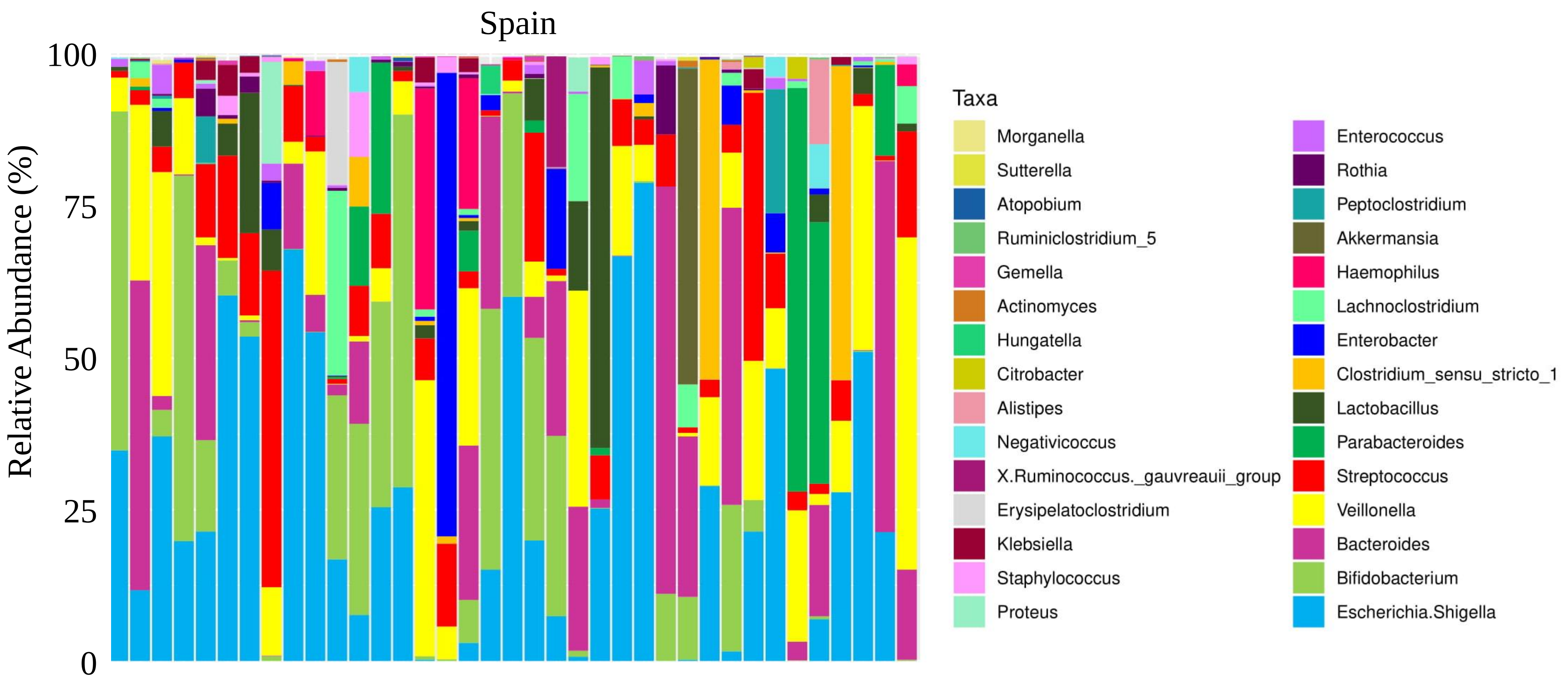

Supplementary Figure 9. Relative abundance of the 30 most-abundant genera in infant fecal samples (*n* =23) collected in Sweden. Each bar represents a single sample.

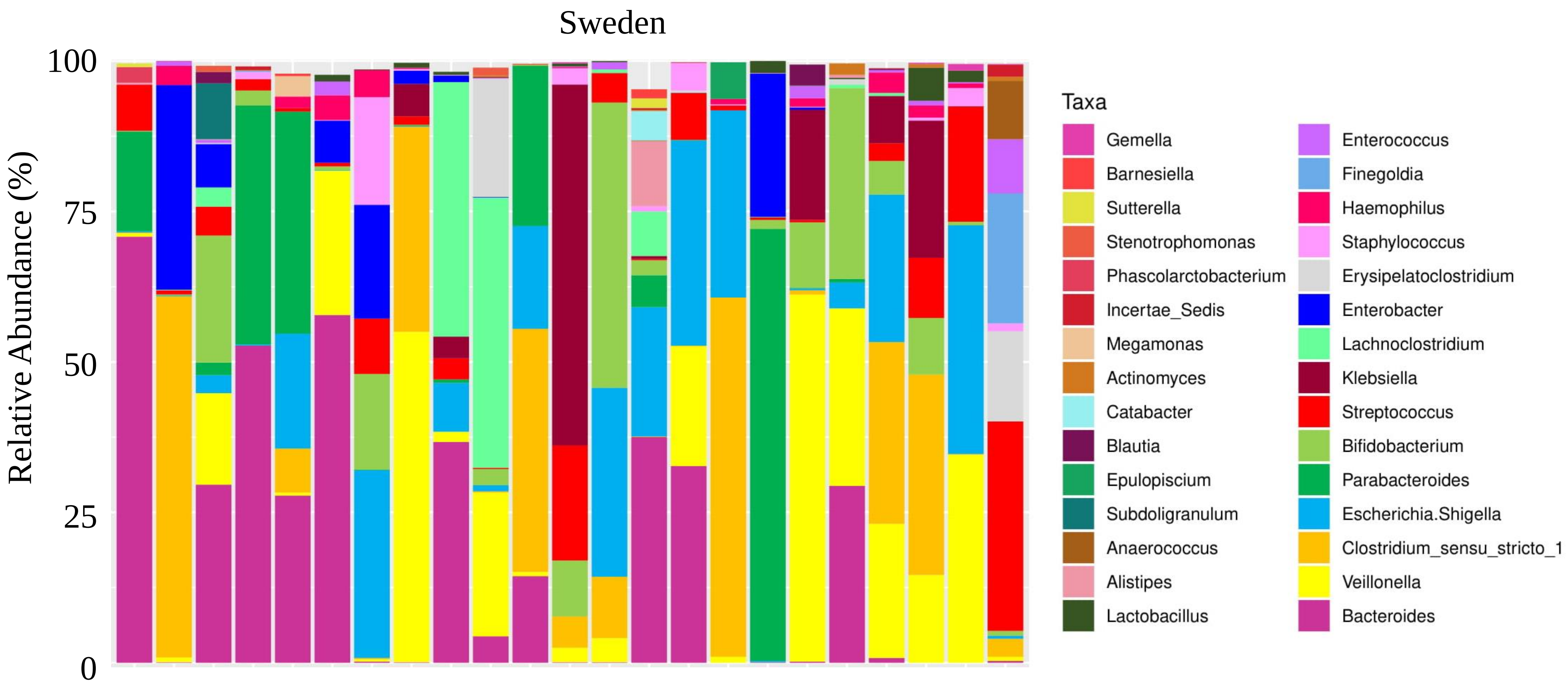

Supplementary Figure 10. Relative abundance of the 30 most-abundant genera in infant fecal samples (*n* = 12) collected in US California. Each bar represents a single sample.

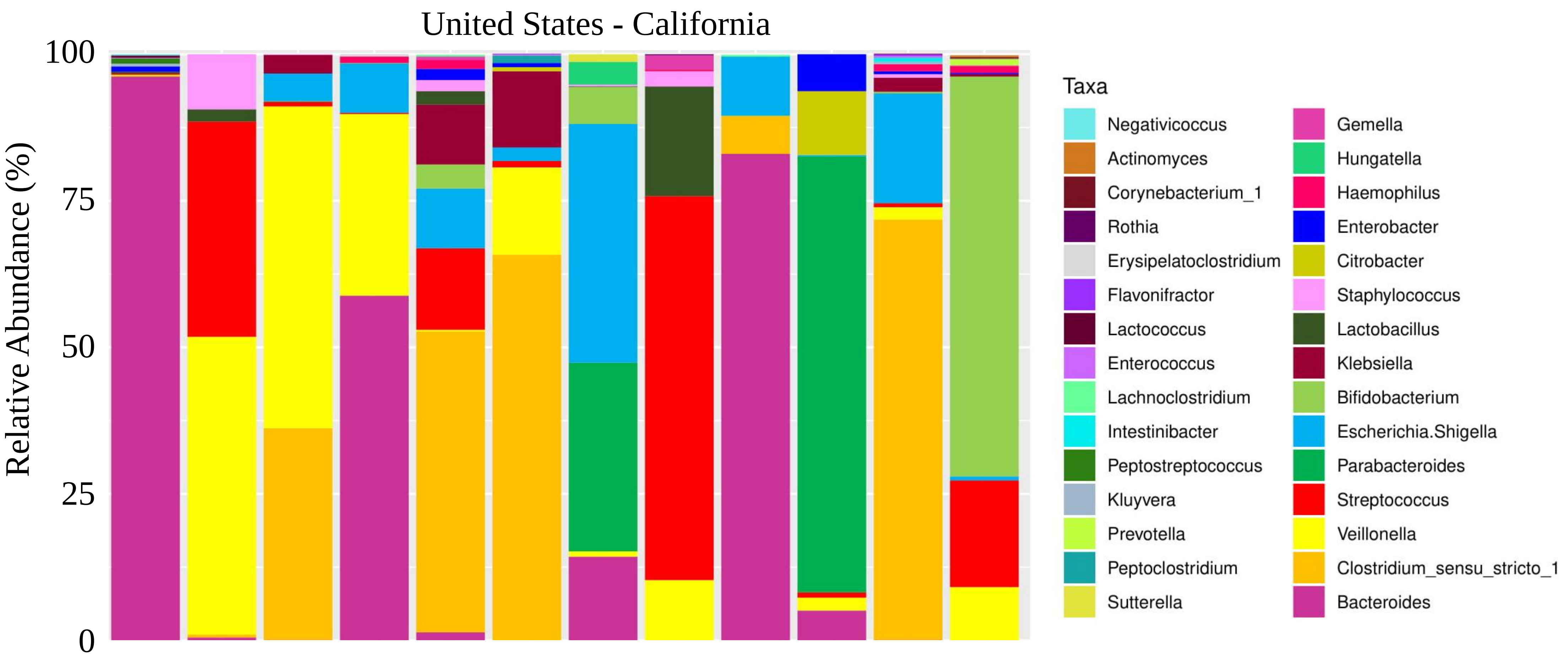

Supplementary Figure 11. Relative abundance of the 30 most-abundant genera in infant fecal samples (*n* = 41) collected in US Washington. Each bar represents a single sample.

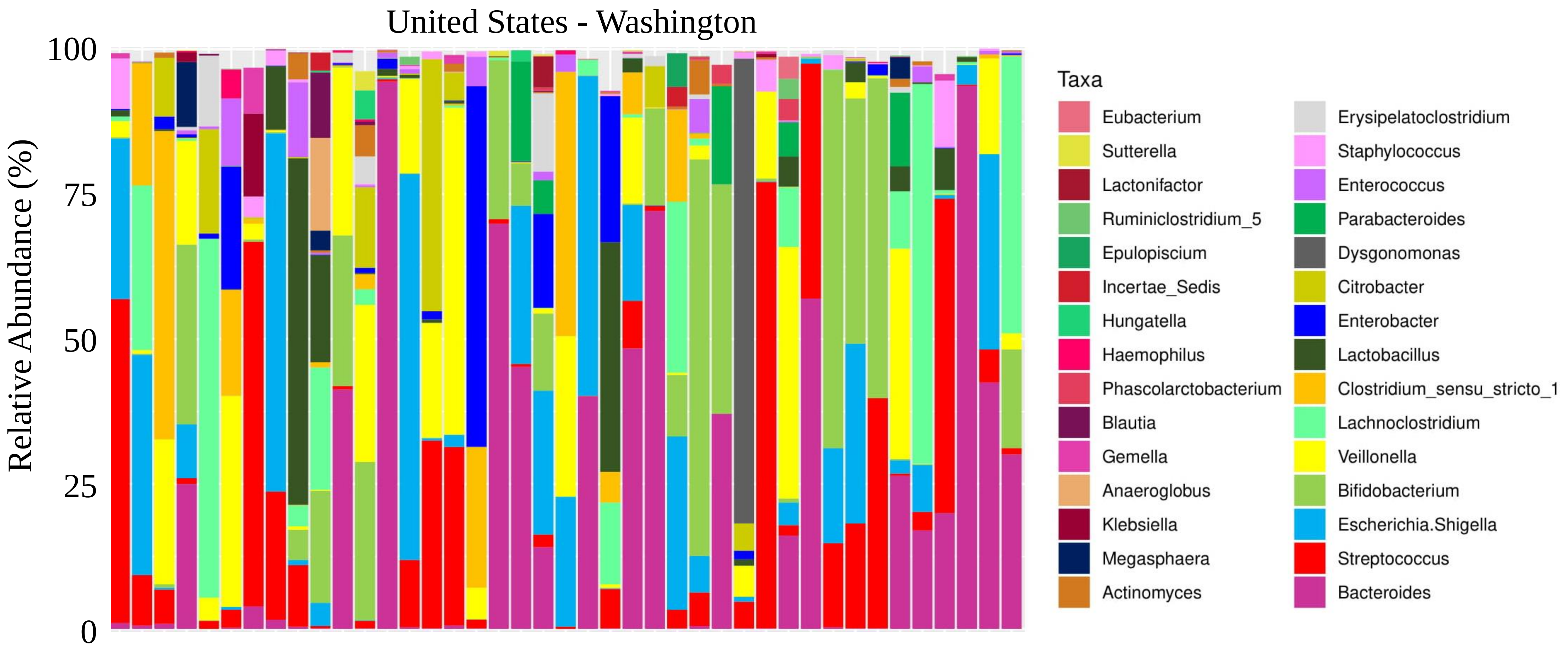

Supplementary Figure 12. Non-metric multidimensional scaling (NMDS) plot visualizing the Bray-Curtis distance by cohort for infant feces (A) and milk (B). Ellipses represent 95% confidence intervals for each cohort.

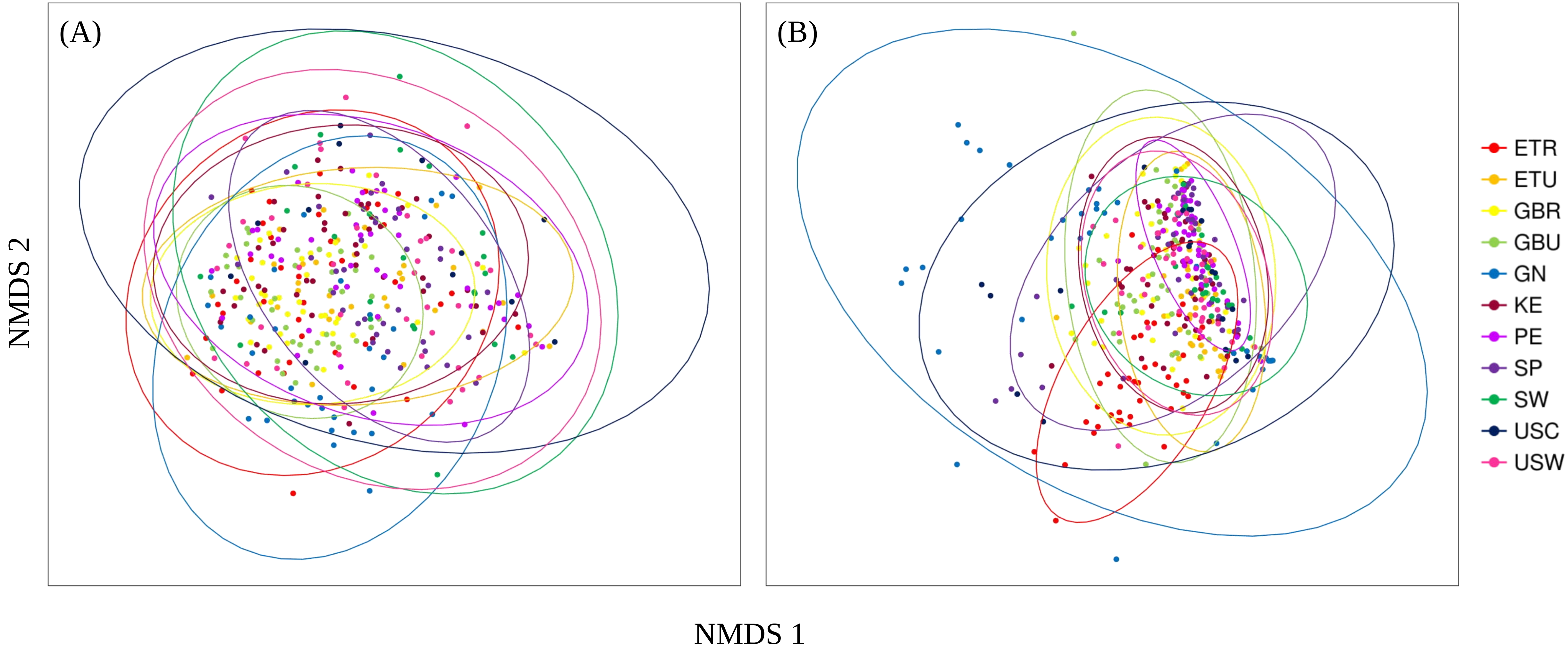

Supplementary Figure 13. Venn diagram showing genera present in at least 90% of samples (core taxa) for each cohort and the overall dataset ("overall"; *n* = 377 for infant feces, *n* = 394 for milk). Abbreviations: ETU, urban Ethiopia; ETR, rural Ethiopia; GBU, urban Gambia; GBR, rural Gambia; GN, Ghana; KE, Kenya; SP, Spain; SW, Sweden; PE, Peru; California (United States), USC; Washington (United States), USW.

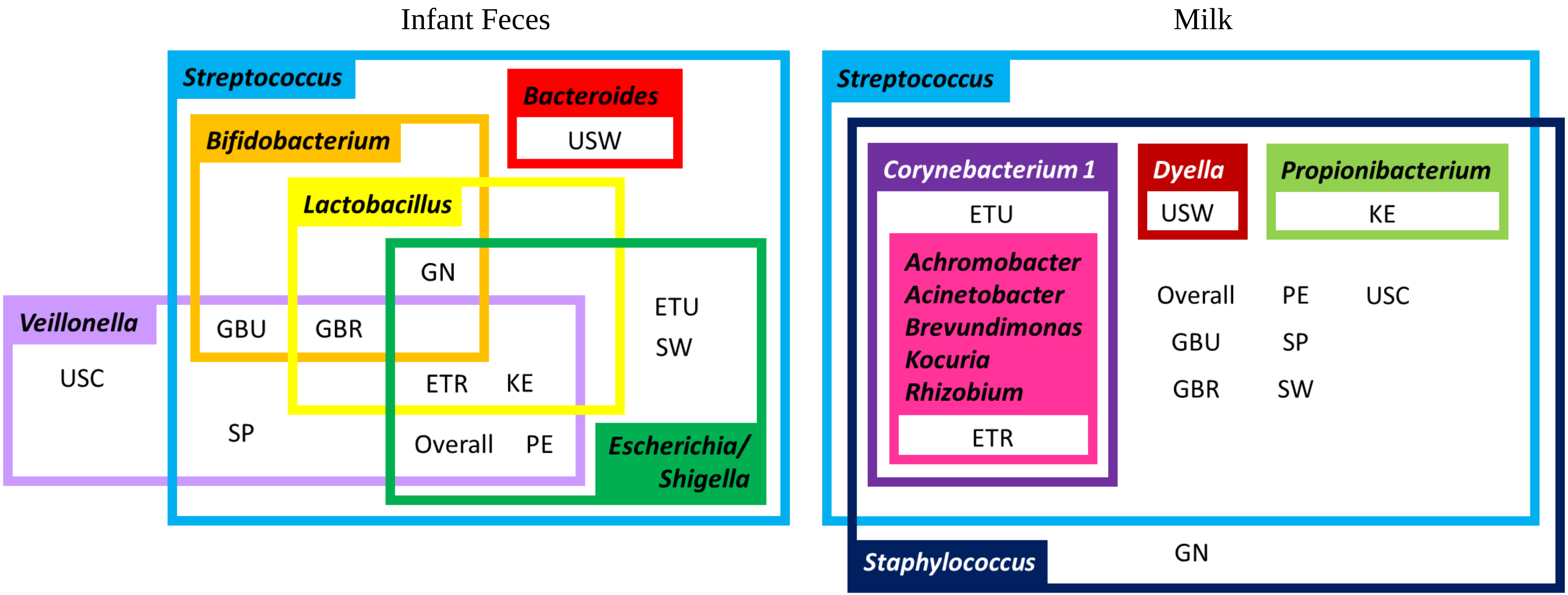

Supplementary Figure 14. Relative abundance of the 30 most-abundant genera in milk samples (*n* = 40) collected in rural Ethiopia. Each bar represents a single sample.

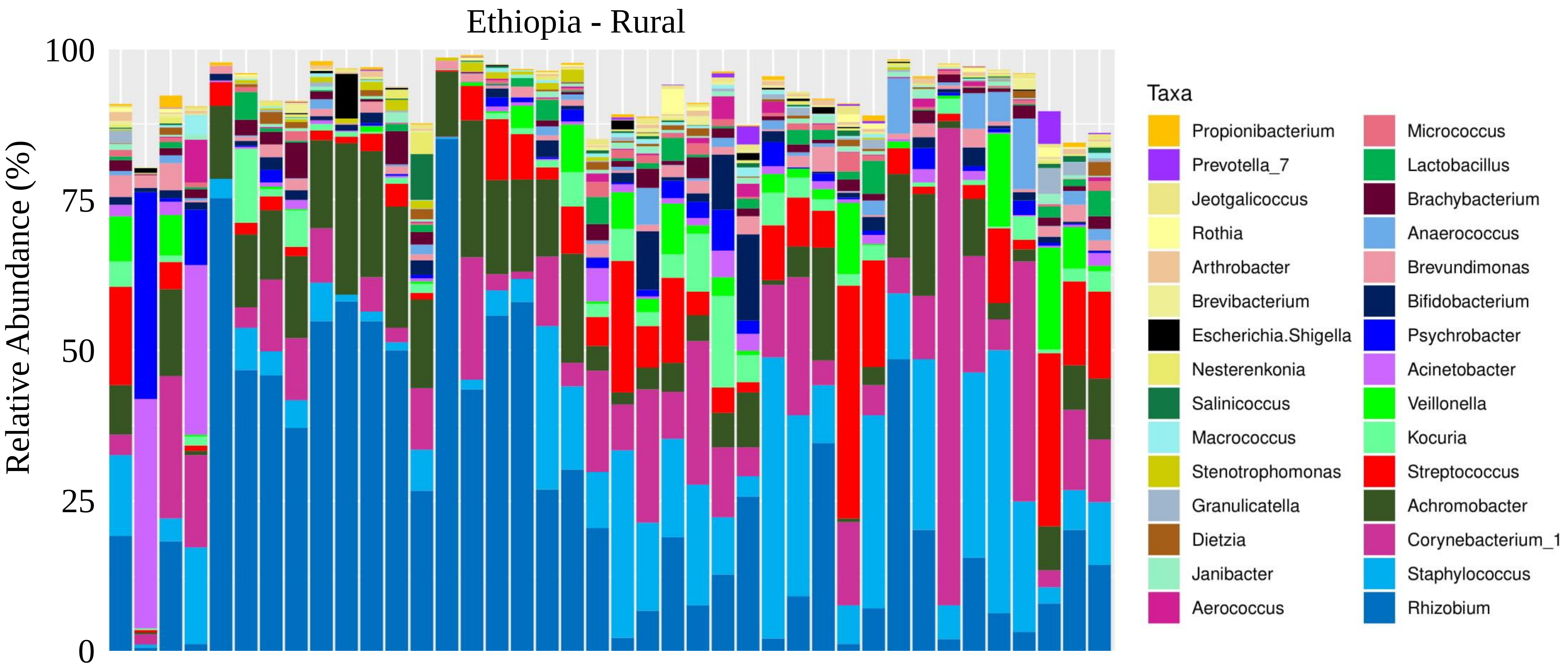

Supplementary Figure 15. Relative abundance of the 30 most-abundant genera in milk samples (n = 34) collected in urban Ethiopia. Each bar represents a single sample.

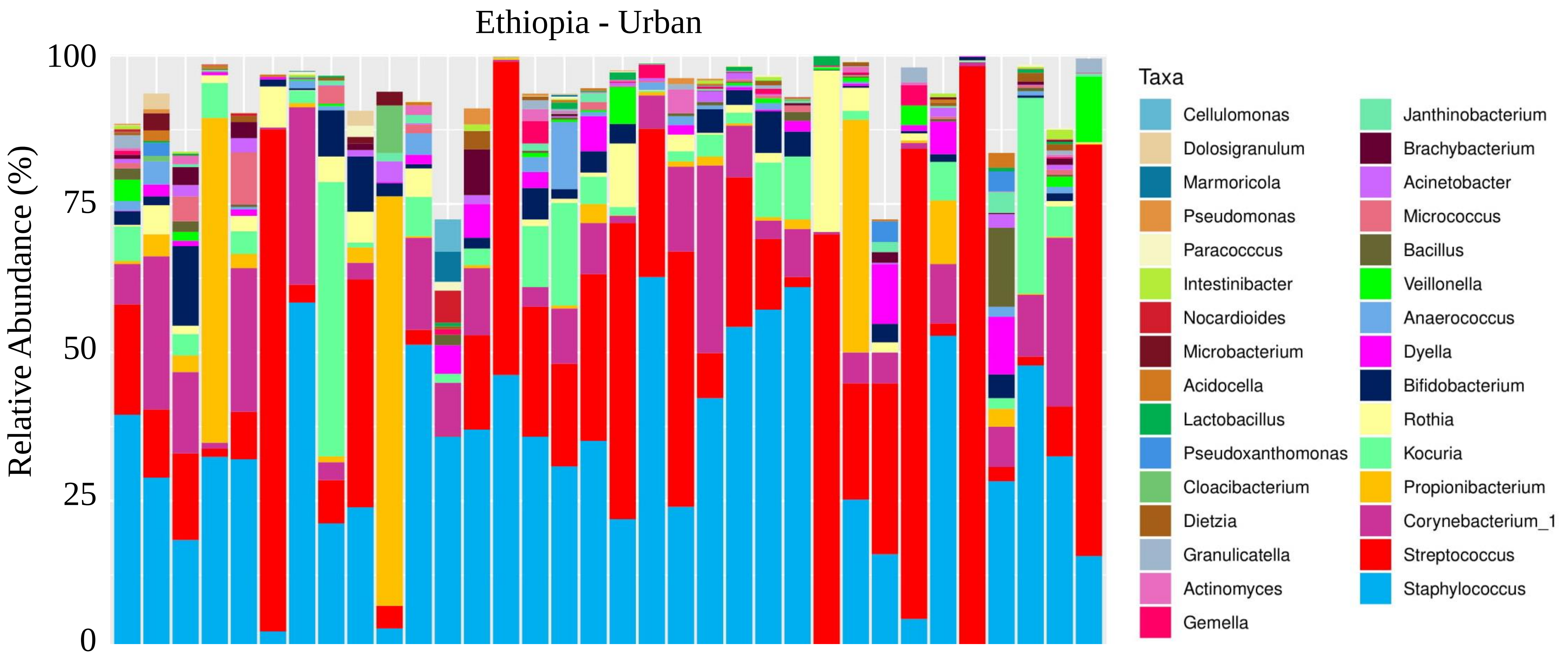

Supplementary Figure 16. Relative abundance of the 30 most-abundant genera in milk samples (*n* = 39) collected in rural Gambia. Each bar represents a single sample.

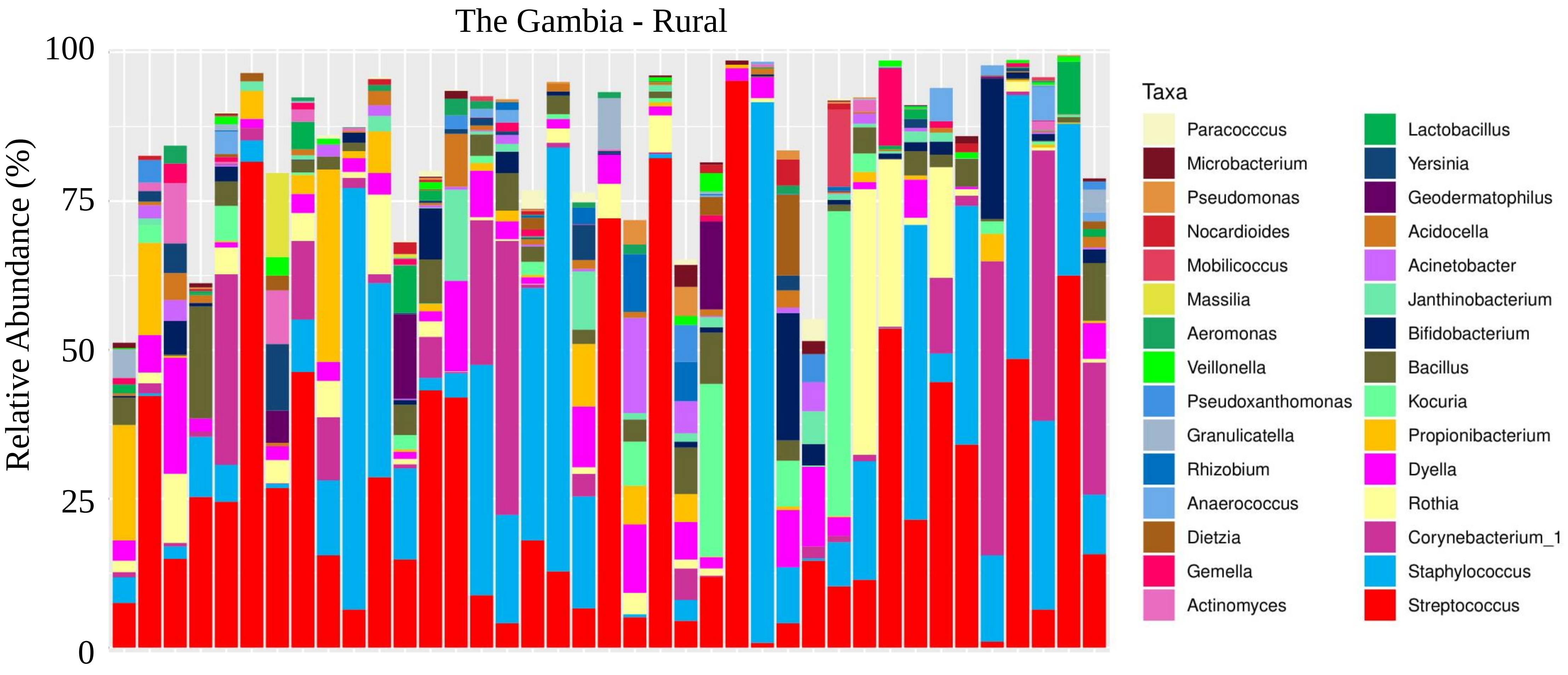

Supplementary Figure 17. Relative abundance of the 30 most-abundant genera in milk samples (*n* = 38) collected in urban Gambia. Each bar represents a single sample.

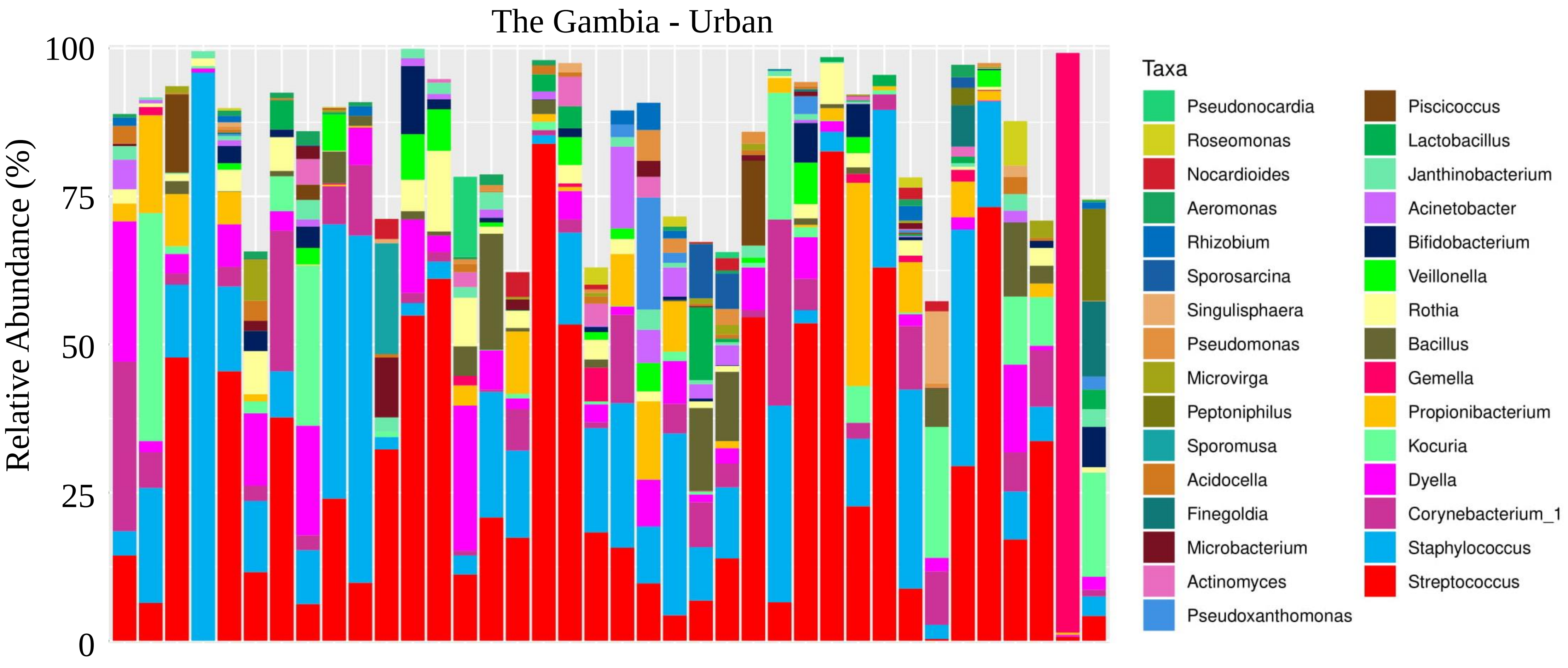

Supplementary Figure 18. Relative abundance of the 30 most-abundant genera in milk samples (*n* = 37) collected in Ghana. Each bar represents a single sample.

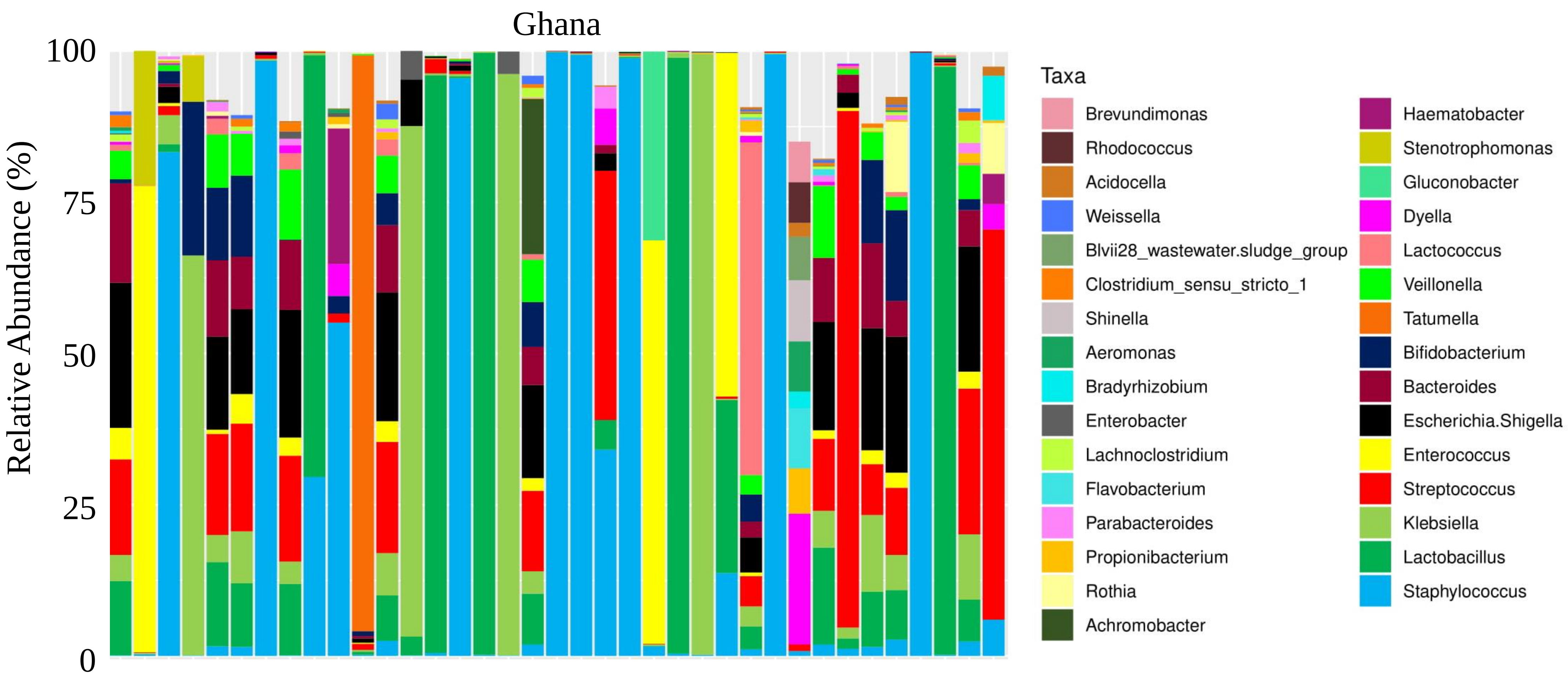

Supplementary Figure 19. Relative abundance of the 30 most-abundant genera in milk samples (n = 43) collected in Peru. Each bar represents a single sample.

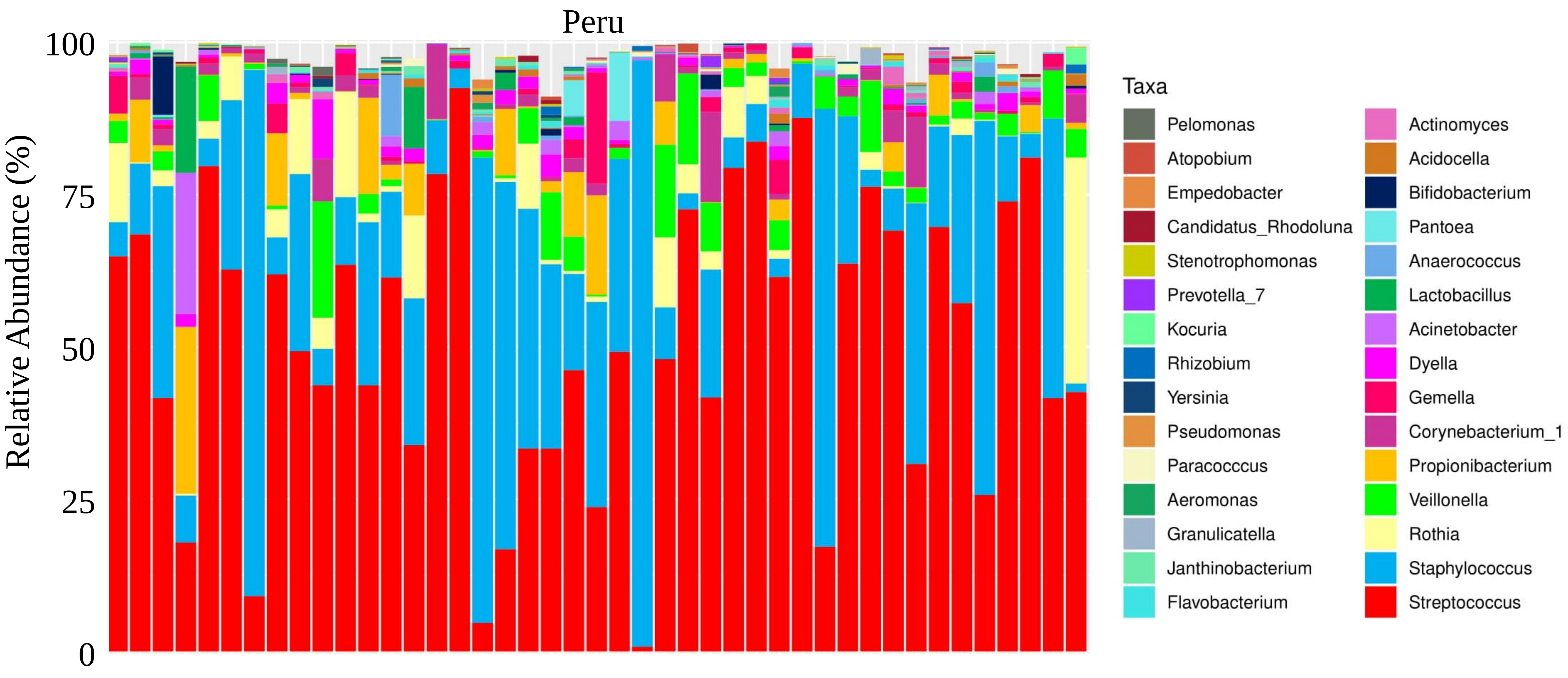

Supplementary Figure 20. Relative abundance of the 30 most-abundant genera in milk samples (n = 42) collected in Kenya. Each bar represents a single sample.

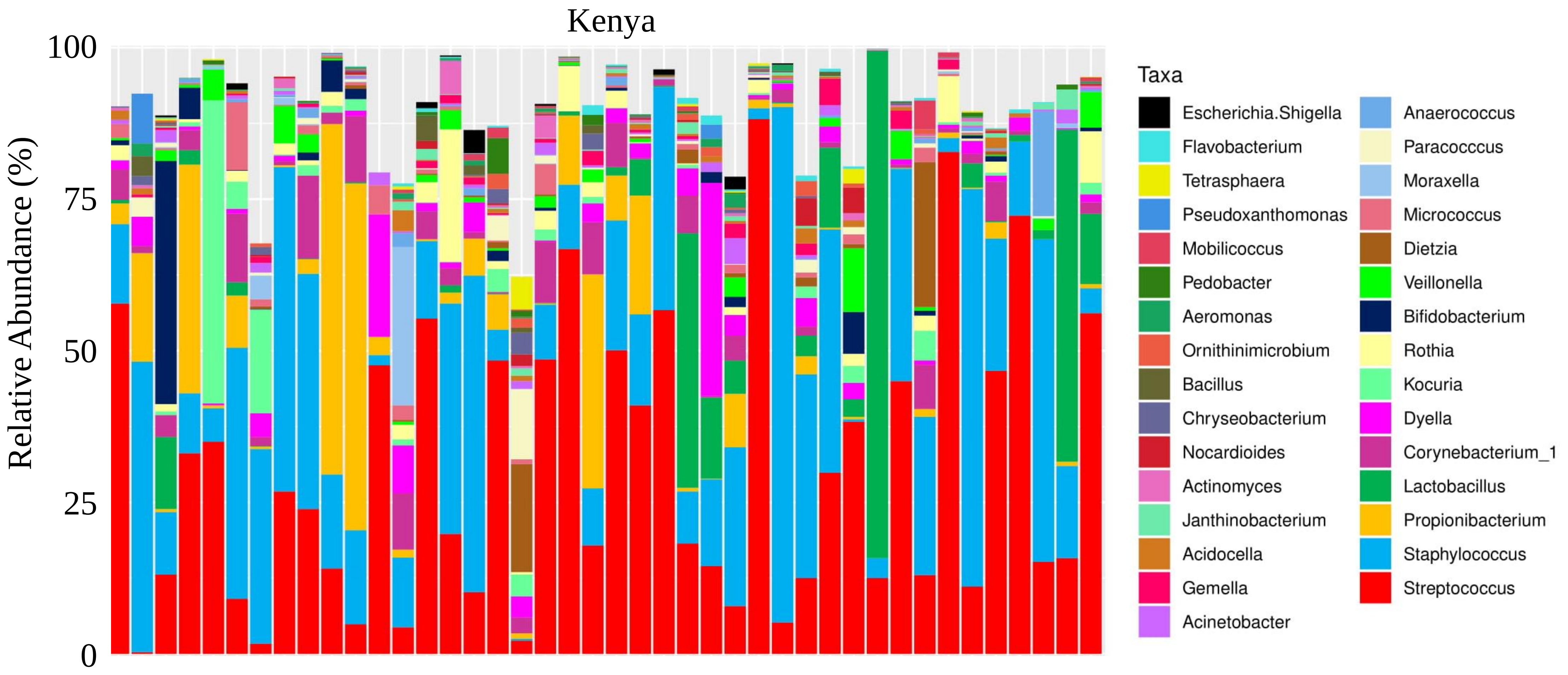

Supplementary Figure 21. Relative abundance of the 30 most-abundant genera in milk samples (*n* = 40) collected in Spain. Each bar represents a single sample.

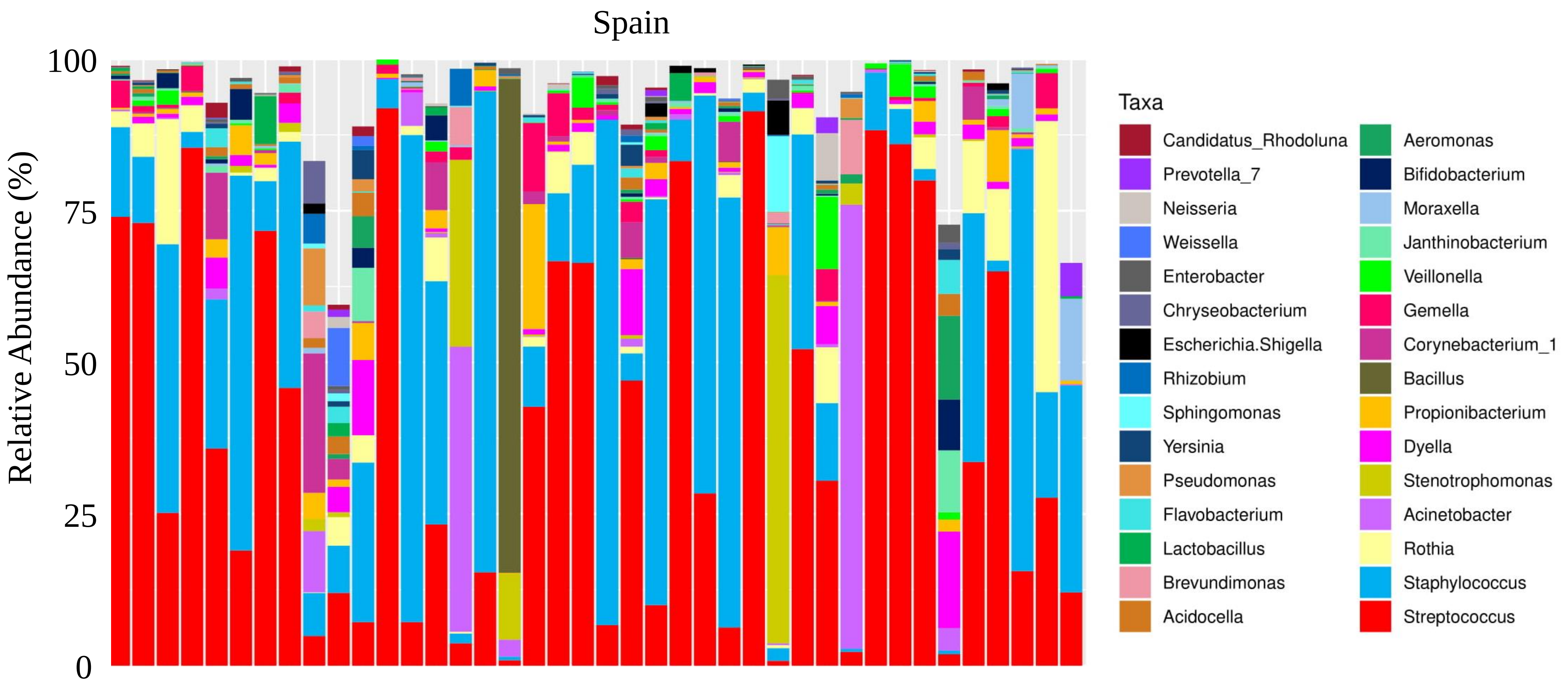

Supplementary Figure 22. Relative abundance of the 30 most-abundant genera in milk samples (*n* = 23) collected in Sweden. Each bar represents a single sample.

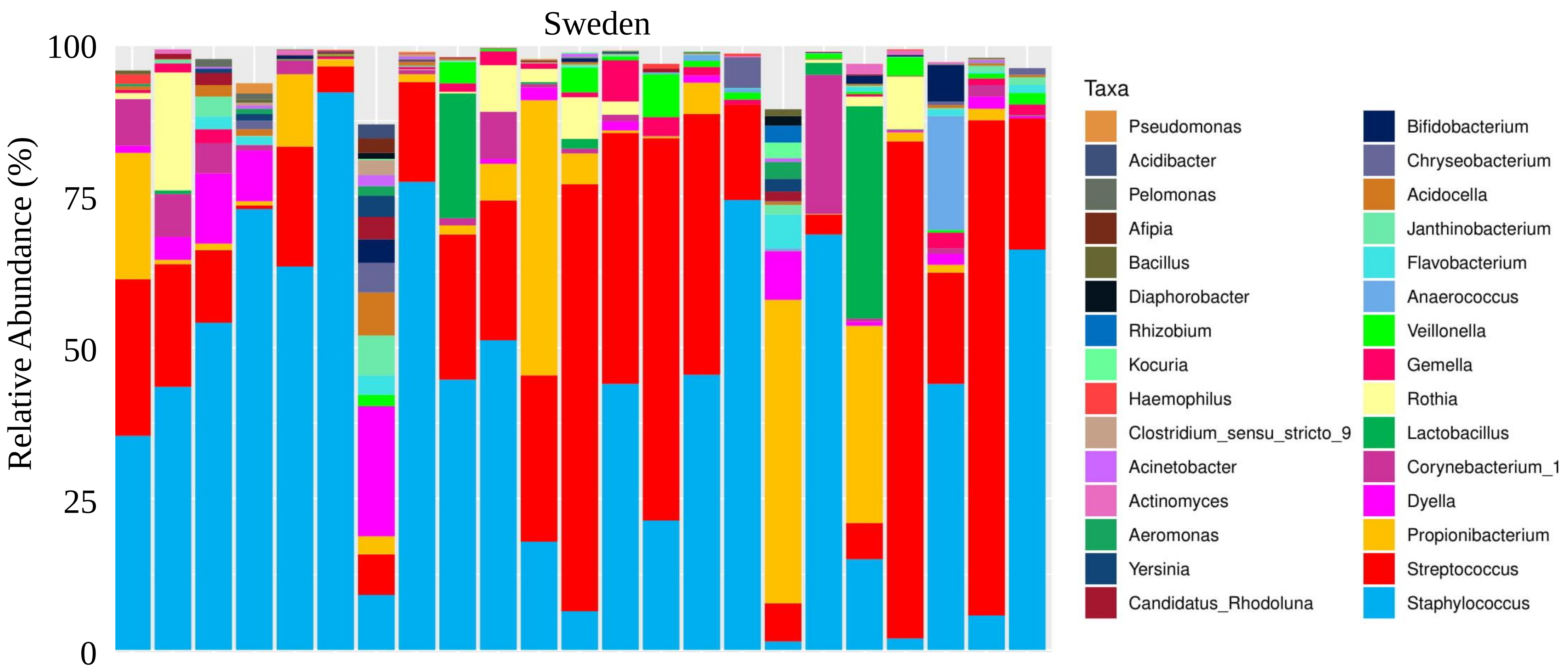

Supplementary Figure 23. Relative abundance of the 30 most-abundant genera in milk samples (n = 19) collected in US California. Each bar represents a single sample.

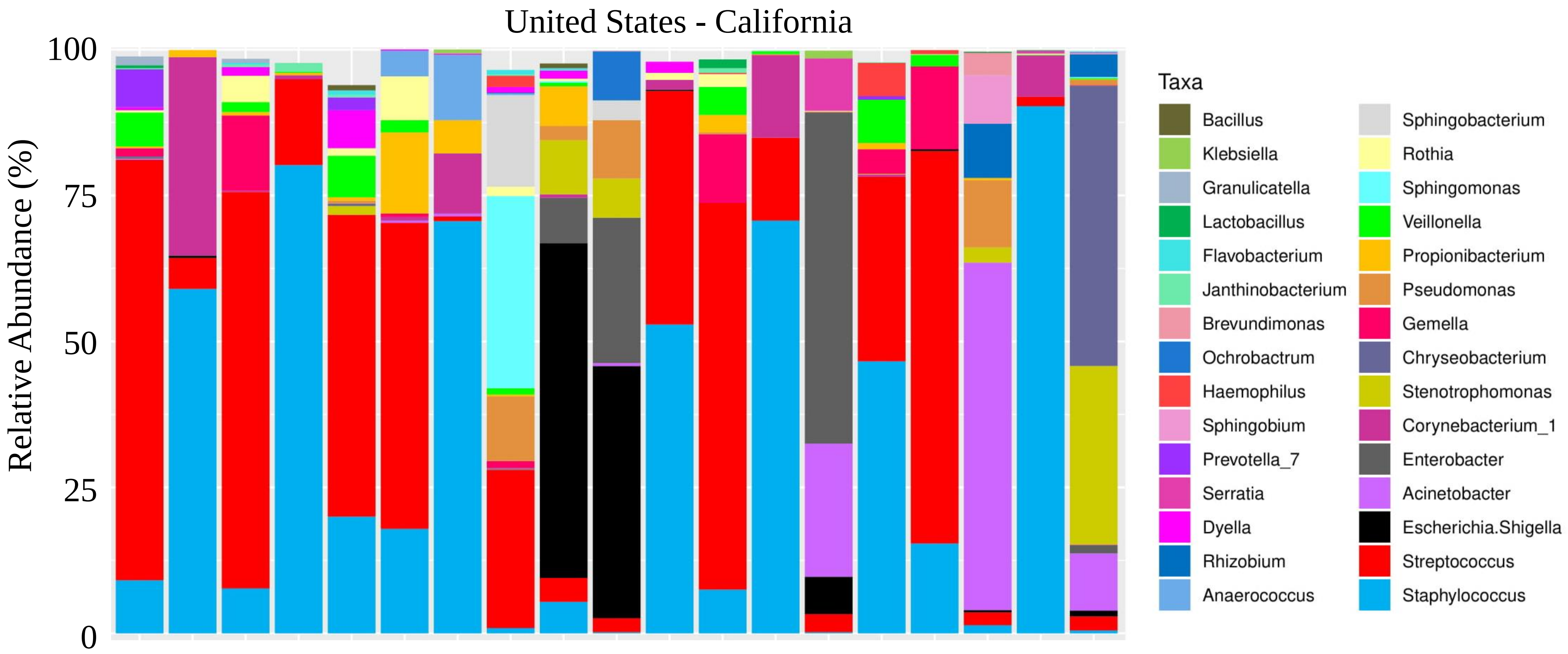

Supplementary Figure 24. Relative abundance of the 30 most-abundant genera in milk samples (*n* = 39) collected in US Washington. Each bar represents a single sample.

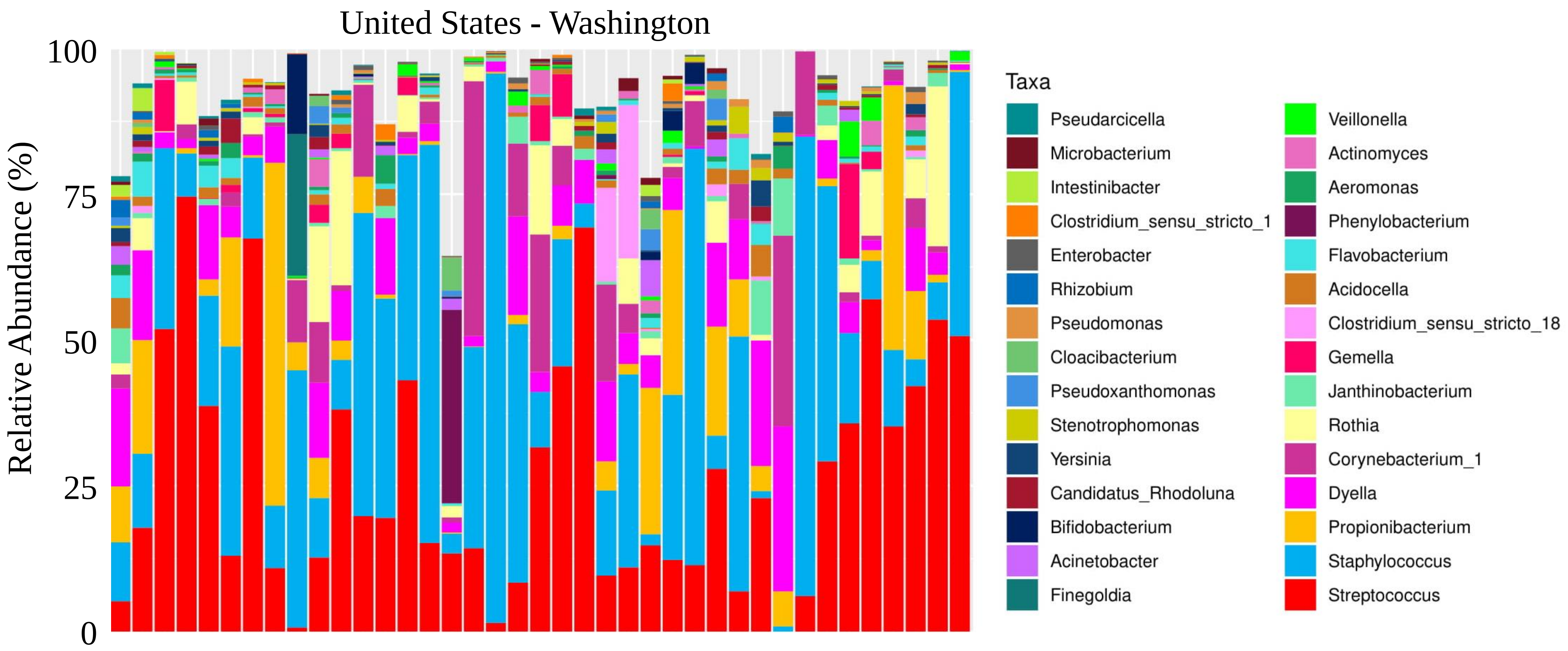

Supplement: Supplementary file 2 [file Data_Sheet_1.PDF]
